# Aldehyde-catalyzed epoxidation of unactivated alkenes with aqueous hydrogen peroxide†

**DOI:** 10.1039/d1sc02360h

**Published:** 2021-06-21

**Authors:** Ierasia Triandafillidi, Maroula G. Kokotou, Dominik Lotter, Christof Sparr, Christoforos G. Kokotos

**Affiliations:** Laboratory of Organic Chemistry, Department of Chemistry, National and Kapodistrian University of Athens Panepistimiopolis 15771 Athens Greece ckokotos@chem.uoa.gr; Department of Chemistry, University of Basel St. Johanns-Ring 19 Basel 4056 Switzerland christof.sparr@unibas.ch

## Abstract

The organocatalytic epoxidation of unactivated alkenes using aqueous hydrogen peroxide provides various indispensable products and intermediates in a sustainable manner. While formyl functionalities typically undergo irreversible oxidations when activating an oxidant, an atropisomeric two-axis aldehyde capable of catalytic turnover was identified for high-yielding epoxidations of cyclic and acyclic alkenes. The relative configuration of the stereogenic axes of the catalyst and the resulting proximity of the aldehyde and backbone residues resulted in high catalytic efficiencies. Mechanistic studies support a non-radical alkene oxidation by an aldehyde-derived dioxirane intermediate generated from hydrogen peroxide through the Payne and Criegee intermediates.

## Abstract 2

An atropisomeric two-axis aldehyde is capable of catalysing the organocatalytic epoxidation of unactivated alkenes using hydrogen peroxide as the oxidant.

## Graphical abstract

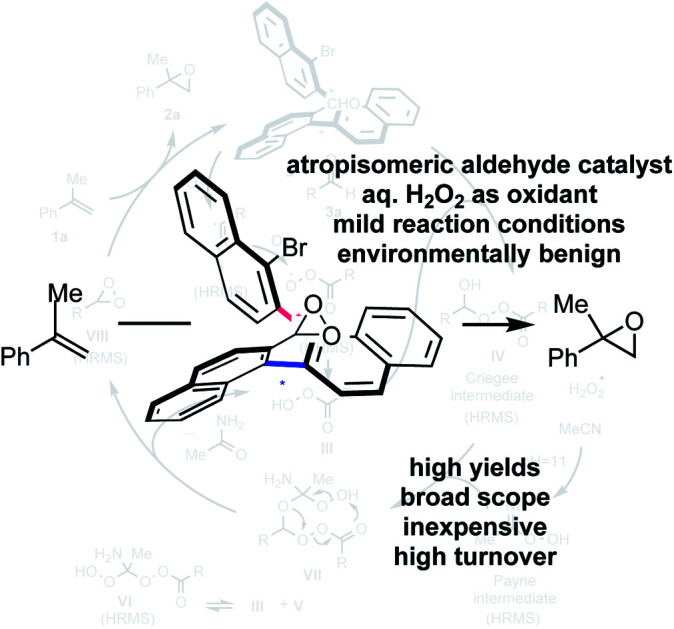

## Introduction

**Scheme 1:**
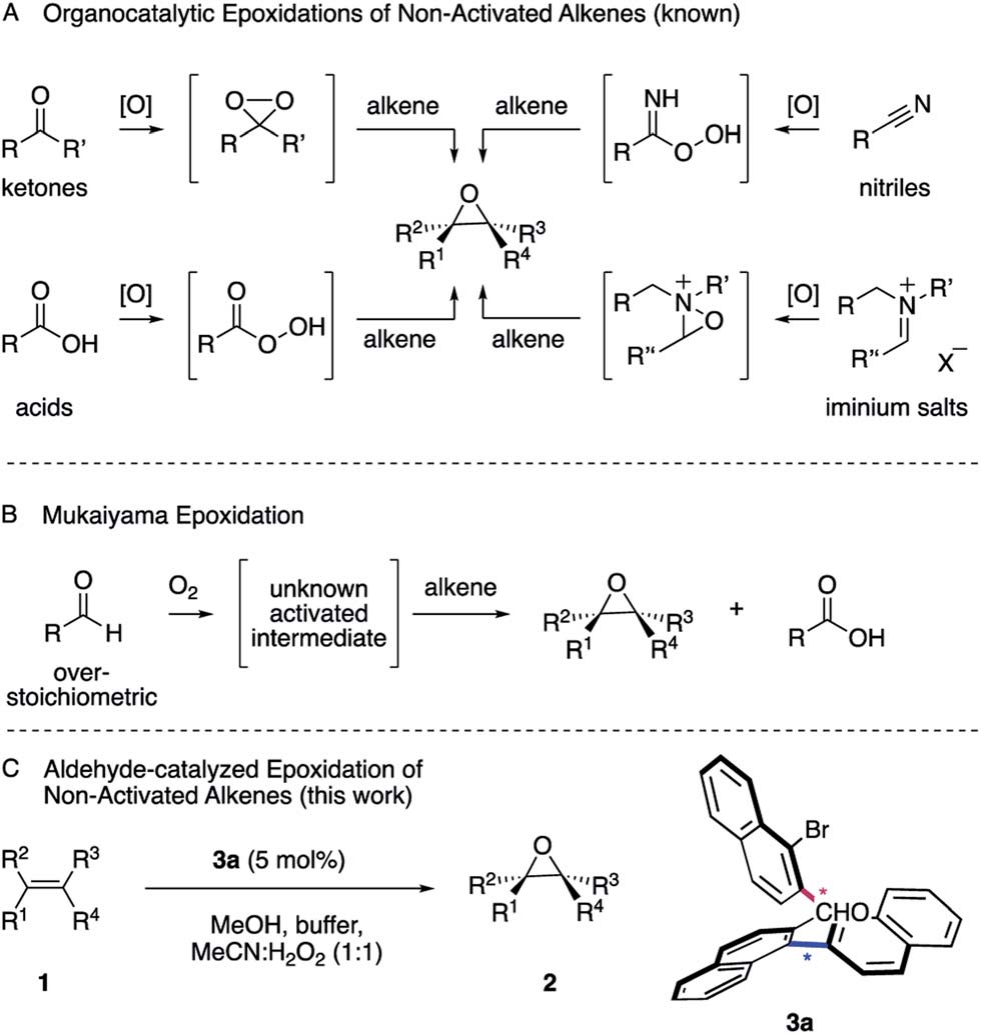
Established epoxidations and conceptualization.

Epoxides are versatile intermediates for the synthesis of countless irreplaceable compounds.^1^ Since the introduction of various versatile metal-catalyzed epoxidations pioneered by Sharpless, Jacobsen, Katsuki and others,^2^ alkene epoxidation has become one of the most studied reactions in organic synthesis, both in industry and academia.^2^ After the successful developments employing metal catalysts, much effort has been devoted to the development of protocols where a more sustainable metal-free catalyst is employed. The organocatalytic epoxidation has flourished^1*a*,1*b*,2^ in particular with ketone,^2^ acid,^3^ nitrile^4^ or iminium salt^2^ catalysts (Scheme 1, **A**). Pioneered by Adam,^5^ Curci,^6^ Yang,^7^ Denmark,^8^ Shi,^9^ Miller,^10^ Page^11^ and others, a variety of oxidants, such as Oxone, *tert*-butyl hydroperoxide (TBHP) or less commonly hydrogen peroxide (H_2_O_2_) were utilized to *in situ* generate the key dioxirane, peracid, imidoperoxoic acid, or oxaziridine intermediates.

Over the last few years, the epoxidation of alkenes with environmentally friendly aqueous H_2_O_2_ ^12^ as the oxidant is drawing increasing attention, since it is inexpensive, relatively safe and affords water as byproduct. However, hydrogen peroxide by itself is a poor oxidant for organic oxidations^2,13^ and thus has to be activated by a catalyst to form a reactive intermediate in order to enable the epoxidation of unactivated alkenes. As one of the most common functionalities in organic chemistry, a wide range of aldehydes are readily available and have been employed as catalysts.^14^ However, one of the most interesting characteristics is its auto-oxidation, where the aldehyde reacts with molecular oxygen, leading to a variety of activated intermediates.^15^ For instance, in the versatile Mukaiyama epoxidation, overstoichiometric amounts of aldehydes have been employed as the mediators of the reaction (Scheme 1, **B**).^16–18^ As a result, a particularly mild and selective process allowed the epoxidation of an exceptionally broad range of alkenes at room temperature employing molecular oxygen as the terminal oxidant. The process can thereby be performed with^16^ or without transition metal catalysts^17^ or in the presence of *N*-hydroxyphthalimide as the catalyst.^18^ The mechanisms of these epoxidations were studied in great detail. Nonetheless, the nature of the active oxygen species remains a subject of debate.^16^ Considering the broad utility of the Mukaiyama epoxidation and the ideal attributes of aqueous H_2_O_2_ as oxidant for sustainable synthesis, we anticipated the development of an aldehyde-catalyzed epoxidation of unactivated alkenes by accommodating the formyl functionality within a spatially well-defined site of an atropisomeric multiaxis system. We describe herein, that the atropisomeric two-axis aldehyde catalyst **3a** that was identified as an efficient catalyst for the activation of aqueous hydrogen peroxide, allows the synthesis of a broad range of epoxides **2** from a variety of unactivated alkenes **1** with notable turnover (Scheme 1, **C**).

## Results and discussion

**Scheme 2:**
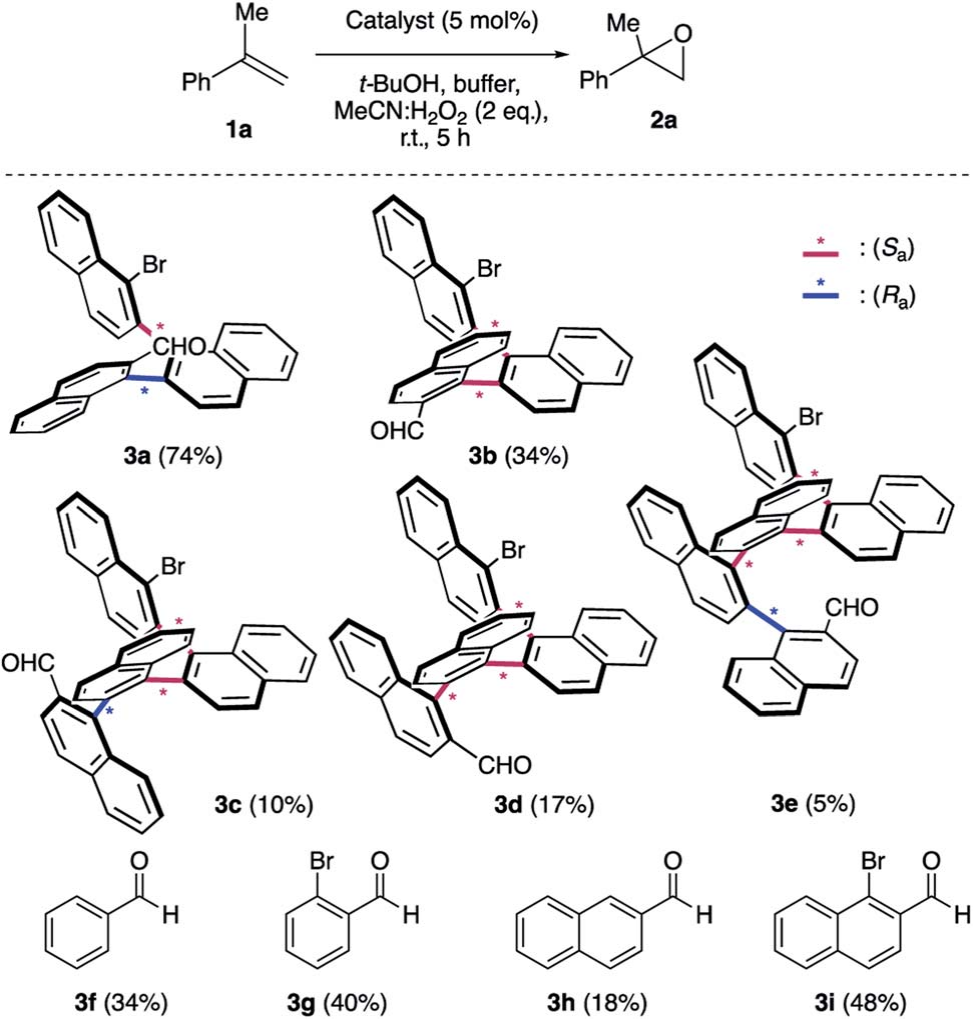
Survey of aldehyde catalysts. ^*a*^K_2_CO_3_/EDTA buffer (pH = 11). The yields were determined by ^1^H NMR using an internal standard.

We initiated our epoxidation studies using several atropisomeric two-, three- and four-axis systems **3a–3e**, which were recently prepared.^19^ To our delight, 5 mol% of catalyst **3a** provided excellent activity for the epoxidation of α-methyl-styrene (**1a**) using H_2_O_2_ as the oxidant (Scheme 2), which is in stark contrast to literature precedents where overstoichiometric amounts of aldehyde were required.^16–18^ We next compared this remarkable finding for the epoxidation of unactivated alkenes with the other atropisomeric aldehydes comprising two to four stereogenic axes, as well as commercially available aldehydes under otherwise identical conditions (2 eq. H_2_O_2_ : MeCN in *t*-BuOH/aq. 0.6 M K_2_CO_3_; 4 × 10^−5^ M EDTA tetrasodium salt, pH = 11). When aldehyde **3a** was employed as the catalyst, the desired epoxide **2a** was formed in 74% yield. Interestingly, the diastereomeric aldehyde **3b** provided epoxide **2a** in significantly lower yield (34%), underscoring the prerequisite spatial positioning of aryl and bromide moieties to augment catalyst turnover. Furthermore, the diastereomeric catalysts **3c** and **3d** with three stereogenic axes as well as the atropisomeric four-axis system **3e** also gave considerably lower yields. While the relative configuration of **3a** notably impacts activity conclusively by governing the conformation of catalytic intermediates, the enantioselectivity remained low for all cases (≤10% ee). The catalytic activity trends were also confirmed with the commercially available aldehydes without rotationally restricted axes (**3f–i**). Interestingly, an enhanced yield was also observed for *ortho*-bromonaphthaldehyde (**3i**) as compared to parent 2-naphthaldehyde (**3h**), supporting the notion that a bromide in proximity to the active site is beneficial for the reaction outcome. Nonetheless, the atropisomeric two-axis aldehyde **3a** outperformed all other catalysts, highlighting the impact of bromine and aryl groups in proximity to the active site to improve the catalytic activity and turnover.

Having identified the unique characteristics of catalyst **3a**, we focused on optimizing the reaction conditions (Table 1). With 5 mol% of **3a** in the presence of 6 eq. H_2_O_2_ and aqueous buffer in *t*-BuOH, the desired α-methyl styrene oxide (**2a**) was formed in 98% yield (entry 2). Furthermore, control experiments without catalyst, oxidant, buffer or MeCN confirmed that all components are necessary for the epoxidation (Table 1, entry 3–6). In agreement with our previous findings,^20*a*,20*b*^ a lower pH provided the desired product in compromised yield, validating that pH = 11 is ideal for the generation of Payne's intermediate^20*b*,21^ (peroxycarboximidic acid **V** from MeCN and H_2_O_2_, Table 1, entry 7 and mechanism in Scheme 5). The importance of the generation of Payne's intermediate for the success of our protocol is also highlighted by the fact that in the absence of MeCN, no epoxidation is taking place (Table 1, entry 6). However, Payne's intermediate alone cannot promote the reaction to completion as can be extracted by the results of Table 1, entry 3. Using different solvents (entries 8–12), methanol was determined as the most effective medium for the reaction, forming the desired α-methyl styrene oxide in quantitative yield (entry 12). On the other hand, a decrease of the catalyst loading to 1 mol% led to a reduced yield of 83% (entry 13). Furthermore, after performing the epoxidation (Table 1, entry 12), the catalyst **3a** was recovered intact (94% recovery by ^1^H-NMR, 82% recovery after column chromatography). To our knowledge, this is the first example, where an aldehyde is employed in substoichiometric amounts as the catalyst for an epoxidation reaction (5 mol% *versus* usually 10–50 mol% of an activated ketone in literature^7–10^ or 5–10 equivalents of pivaldehyde^16–18^).

**Table 1:** Optimization of the reaction conditions^a^

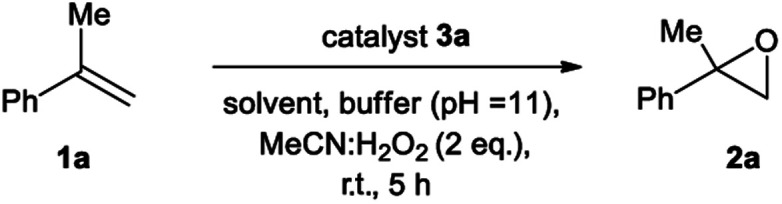				
Entry	**3a** [mol%]	Solvent	Deviation	Yield (%)
1	5.0	*t*-BuOH	—	74%
2	5.0	*t*-BuOH	6 eq. H_2_O_2_	98%
3	0	*t*-BuOH	—	9%
4	5.0	*t*-BuOH	No H_2_O_2_	0%
5	5.0	*t*-BuOH	No buffer	0%
6	5.0	*t*-BuOH	No MeCN	0%
7	5.0	*t*-BuOH	pH = 10	41%
8	5.0	EtOAc	—	67%
9	5.0	MeCN	—	82%
10	5.0	CH_2_Cl_2_	—	15%
11	5.0	CHCl_3_	—	12%
12	5.0	MeOH	—	**quant.**
13	1.0	MeOH	—	83%

a K_2_CO_3_/EDTA buffer (pH = 11). Yield determined by ^1^H NMR using an internal standard.

**Scheme 3:**
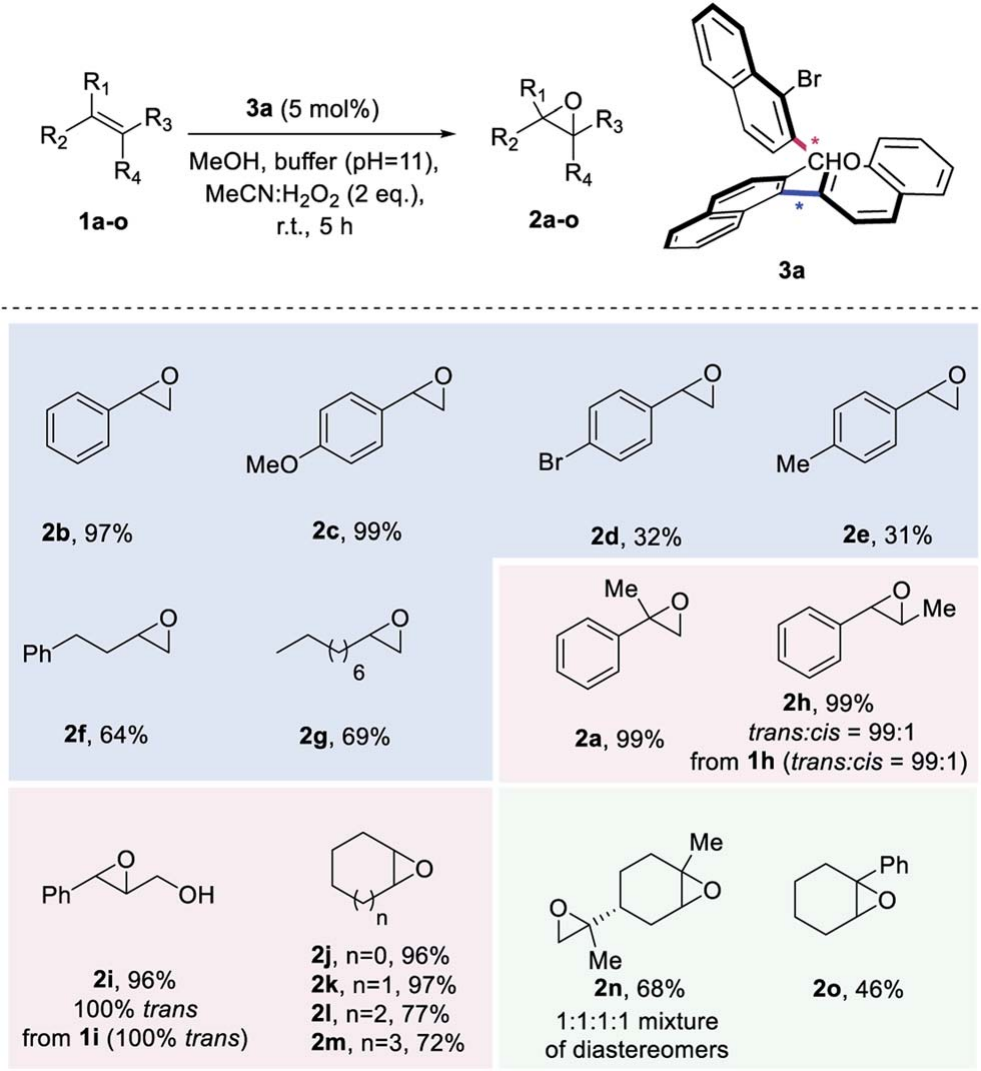
Substrate Scope. ^*a*^K_2_CO_3_/EDTA buffer (pH = 11). Yields of products after isolation by column chromatography.

**Scheme 4:**
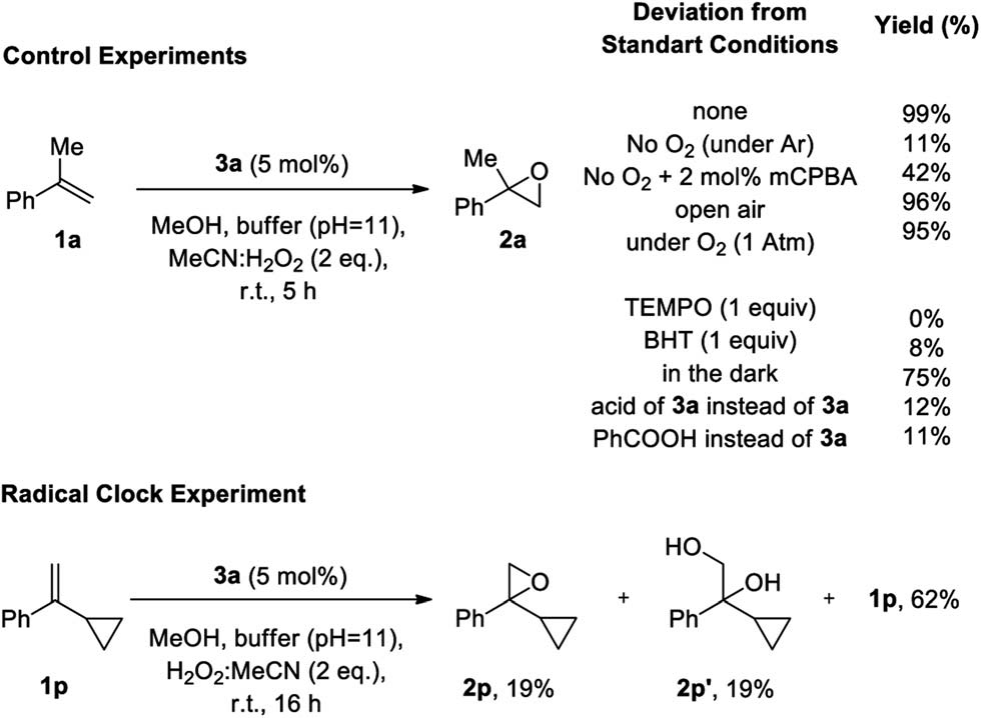
Mechanistic and radical clock experiments.

**Scheme 5:**
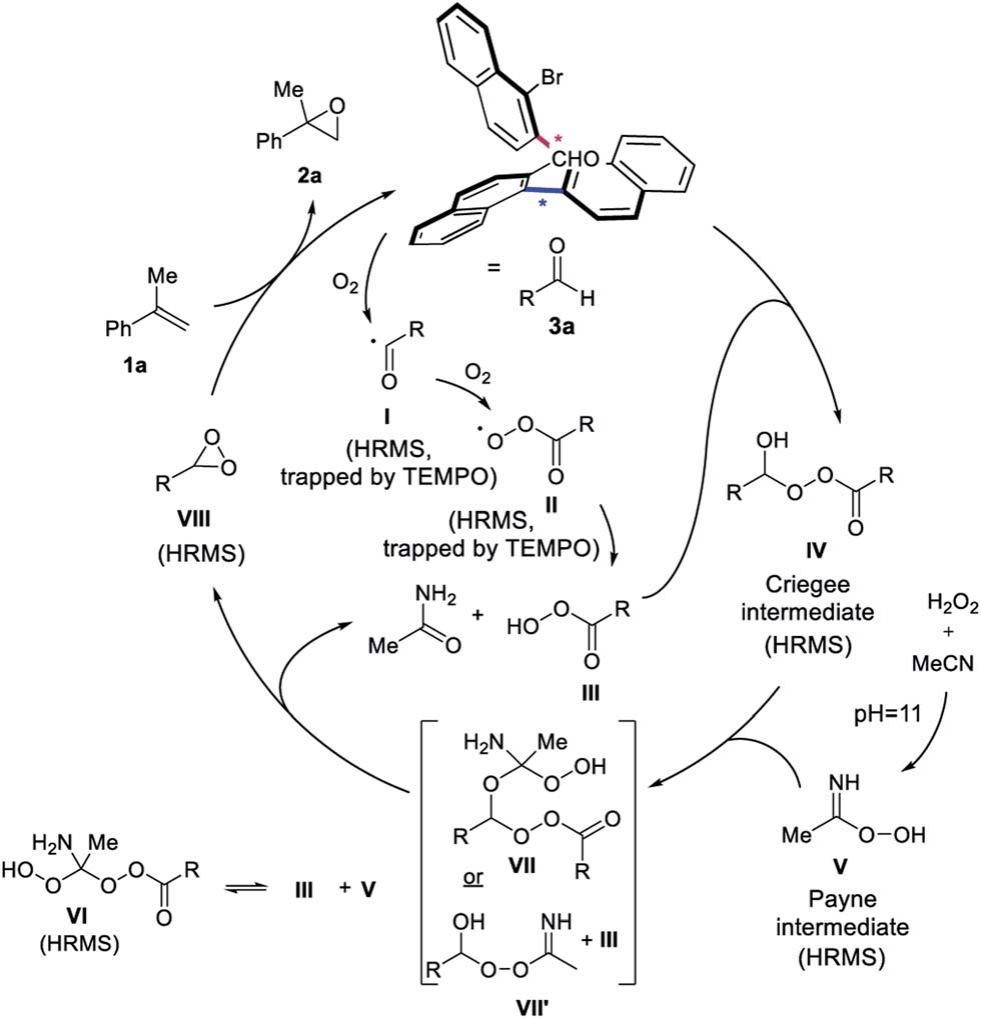
Proposed mechanism.

With the optimized reaction conditions defined, we set out to explore the substrate scope of the aldehyde-catalysed epoxidation of unactivated alkenes (Scheme 3). A variety of monosubstituted styrenes were first tested, providing excellent isolated yields for the unfunctionalized and methoxy-substituted styrenes (**2b** and **2c**), but low yields for **2d** and **2e** (in the case of **2e**, 28% of the corresponding diol was also isolated). In contrast to known procedures which lead to low amounts of epoxide when terminal aliphatic alkenes are employed, synthetically meaningful yields were obtained for corresponding epoxides **2f** and **2g**. Gratifyingly, when studying disubstituted alkenes, geminal and vicinal disubstituted olefins afforded an excellent outcome [**2a**, **2h** (99 : 1 *trans* : *cis* from 99 : 1 *trans* : *cis***1h**) and **2i** (only *trans* from only *trans***2i**)] and also cyclic disubstituted alkenes provided epoxides in up to 97% isolated yield (**2j–2m**). To explore the limitations of the method, also trisubstituted unactivated alkene substrates were investigated, leading to a 68% yield for a double epoxidation to product **2n** and an inferior performance for aryl-substituted **2o**. Unfortunately, tetrasubstituted alkenes or linear *cis* alkenes are not viable substrates for this method, as in the former case, the desired epoxide is not formed, while in the latter case, low yields (around 16%) are observed.

Due to the novelty of aldehyde catalysis for epoxidations with hydrogen peroxide, mechanistic studies to probe the reaction pathway of this epoxidation were carried out (Scheme 4). In agreement with analogous studies on the auto-oxidation of aldehydes,^15*a*,15*b*,15*d*,16*i*,18*b*^ control experiments for the epoxidation of **1a** to **2a** support a mechanism involving an auto-oxidation of aldehyde **3a**. When the reaction was performed under an argon atmosphere (Scheme 4), only 11% product formation was observed, emphasizing the involvement of oxygen in the reaction in agreement with the epoxidation occurring through the Payne intermediate (see Table 1, entry 3). In contrast, almost quantitative yields were obtained by performing the reaction under open air or a balloon of oxygen, verifying the notion that oxygen is a constituent of the mechanism. According to literature,^15*a*,15*b*,15*d*,16*i*,18*b*^ oxygen is required for the generation of the Criegee intermediate **IV**.^22^ Indeed, adding 2 mol% of *m*CPBA in the mechanistic experiment that was performed in the absence of oxygen, the epoxidation reaction can be restored to a good extent (42% yield, see below). Furthermore, if TEMPO or BHT were added to the reaction as a radical trap, no epoxidation product was formed, verifying the generation of radical intermediates in the autooxidation of the catalyst. When the reaction was performed in the dark, the yield of the desired epoxide **2a** remained constant, proving that the reaction mechanism does not follow a photochemical pathway. In order to exclude the possibility that the epoxidation is promoted by an *in situ* generated peracid, we substituted catalyst **3a** by its acid or alternatively by PhCOOH and observed a very low yield, which again can be assigned to the reactivity of the Payne intermediate. This is in agreement with literature,^10^ where an acid in combination with MeCN/H_2_O_2_ cannot promote the epoxidation reaction. These results in combination with the almost quantitative recovery of **3a** from the reaction mixture, verify that a peracid is not responsible for the epoxidation, since in that case, **3a** could not have been recovered intact and should have been oxidized to the corresponding acid,^23^ which is not capable of performing the epoxidation. In order to test the nature of the active intermediate that enables the epoxidation of the unactivated alkenes, we performed the epoxidation with the radical clock **1p**. Epoxide **2p** was observed in 19% yield, along with 19% of diol **2p′** and 62% of unreacted **1p**, without any ring-opened product detected. This finding supports that the active oxidant is of non-radical nature, and that the radical steps during autooxidation of the aldehyde precede the epoxidation step.

A proposed mechanism consistent with the findings consequently involves the initial autooxidation of a fraction of the aldehyde **3a** under aerobic conditions, forming radical intermediate **I** (Scheme 5).^15*a*,15*b*,15*d*,16*i*,18*b*^ The peroxy-radical intermediate **II** and peracid **III** is subsequently formed with oxygen.^15^ The addition of peracid **III** to aldehyde **3a** provides the Criegee intermediate **IV**.^15*b*,22^ At pH = 11, the Payne intermediate **V** is generated,^4,20*b*,21^ which is in equilibrium with **VI** from a reaction with peracid **III**. The Criegee and Payne intermediates (**IV** + **V**) result in a postulated species **VII**, which breaks up to form the key dioxirane intermediate **VIII**, the acetamide byproduct and regenerates peracid **III**. Another plausible pathway is the transformation of intermediate **IV** and **V**, to form **III** and **VII′**, which can also give rise to dioxirane **VIII** and acetamide. The dioxirane intermediate **VIII** as the active oxidant then reacts with unactivated alkene **1a** to form epoxide **2a**, regenerating aldehyde **3a** to close the catalytic cycle. The crucial role of the buffer for the generation of intermediate **V** is highlighted in the control experiments, as well as the inability of **V** alone to push the reaction to completion. Oxygen from air is required for the autooxidation of the aldehyde to form intermediate **IV**. A small amount of peracid is necessary to promote the catalytic cycle *via* the generation of **IV** and not to perform the epoxidation, as highlighted by the recovery of **3a** after reaction completion and the reinstatement of the epoxidation yield when catalytic *m*CPBA was added to the control experiment under argon. A finding consistent with the formation of dioxirane **VIII** is that **3a** under Shi-like conditions with Oxone as the oxidant, simulating the formation of the dioxirane-intermediate, provided the desired epoxide **2a** in good yield (65%).^24^

Considering this mechanistic hypothesis, we examined the possible pathways of the reaction by High Resolution Mass Spectrometry (HRMS, see ESI† for details) and thereby detected several of the intermediates of the proposed pathway. Adding TEMPO as the radical scavenger to the reaction mixture, the adducts of **I** and **II** (trapped with TEMPO) were identified, supporting that the autoxidation step is occurring. The Criegee intermediate **IV** and both intermediates **V** and **VI** were also detected, corroborating the formation of the Payne intermediate, when using the appropriate aqueous buffer (pH = 11). Moreover, also the central alkene oxidant dioxirane **VIII** was detected by HRMS. In order to rule out artifacts, the measurements were also repeated using CD_3_CN or 2-bromobenzaldehyde (**3g**) to detect similar intermediates, such as the corresponding Criegee intermediate and the dioxirane by HRMS, thus further underpinning the proposed mechanism.^24^ Intermediate **VII** was observed in the case of **3g** with CD_3_CN, while **VII′** was detected with **3a** in the presence of TEMPO. In accord with these findings, both short-lived intermediates provide exploratory support of an aldehyde-catalyzed epoxidation by means of dioxirane formation.

## Conclusions

In conclusion, we have developed a sustainable and efficient method for the epoxidation of unactivated alkenes utilizing an atropisomeric multiaxis aldehyde as the catalyst in combination with hydrogen peroxide as the oxidant. This study reveals the feasibility of aldehyde-catalyzed epoxidations and the mechanistic studies shed first light on this unique reactivity. To our knowledge this is the first example of an epoxidation reaction that employs an aldehyde in catalytic amounts. Ongoing studies aim to rationalize the individual requirements for catalyst design to further increase catalytic efficiency and to accomplish stereoselectivity.

## Data availability

Experimental data are available from the authors upon request.

## Author contributions

I. T., C. S. and C. G. K. conceived the study, designed the experiments, and analyzed the data. I. T. performed the experiments and M. G. K. the HRMS studies. D. L. provided catalysts **3b–3e**. The manuscript was written through contributions of all authors.

## Conflicts of interest

There are no conflicts to declare.

## Supplementary Material

SC-012-D1SC02360H-s001
