# Supplementary material for: Aldehyde-catalyzed epoxidation of unactivated alkenes with aqueous hydrogen peroxide
Source: Chem Sci. 2021 Jun 21;12(30):10191–6. doi: 10.1039/d1sc02360h (PMC8336450; doi:10.1039/d1sc02360h)
Supplement: SC-012-D1SC02360H-s001 [file SC-012-D1SC02360H-s001.pdf]

Electronic Supplementary Material (ESI) for Chemical Science.
This journal is © The Royal Society of Chemistry 2021

SUPPORTING INFORMATION

Aldehyde-Catalyzed Epoxidation of Unactivated Alkenes with Aqueous Hydrogen Peroxide

Ierasia Triandafillidi,[a,b] Maroula G. Kokotou,[a] Dominik Lotter,[b] Christof Sparr[b]* and Christoforos G. Kokotos[a]*

[a] Laboratory of Organic Chemistry, Department of Chemistry, National and Kapodistrian University of Athens,
Panepistimiopolis, Athens 15771, Greece

[b] Laboratory of Organic Chemistry, Department of Chemistry, University of Basel,
St. Johanns-Ring 19, Basel 4056, Switzerland

Table of Contents

General Methods **S2**
Synthesis of the Catalysts **S3**
Catalyst Evaluation **S6**
Optimization of the Aldehyde-catalyzed Epoxidation **S7**
General Procedure and Substrate Scope **S8**
Control Experiments and Radical Clock Experiment **S14**
High Resolution Mass Spectrometry Studies **S17**
References **S66**

General Methods

Chromatographic purification of products was accomplished using forced-flow chromatography on Merck® Kieselgel 60 F_{254} 230-400 mesh. Thin-layer chromatography (TLC) was performed on aluminum backed silica plates (0.2 mm, 60 F_{254}). Visualization of the developed chromatogram was performed by fluorescence quenching using phosphomolybdic acid, anisaldehyde or potassium permanganate stains. Mass spectra (ESI) were recorded on a Finningan® Surveyor MSQ LC-MS spectrometer. HRMS spectra were recorded on Bruker® Maxis Impact QTOF spectrometer. ^{1}H, ^{19}F and ^{13}C NMR spectra were recorded on Varian® Mercury (200 MHz, 188 MHz and 50 MHz), a Brucker DPX-400 (400 MHz, 376 MHz and 100 MHz) or on a Bruker Avance III (400 MHz, 376 MHz and 100 MHz) spectrometer in $CDCl_3$ and are internally referenced to residual solvent signals. Data for ^{1}H NMR are reported as follows: chemical shift (*δ* ppm), integration, multiplicity (s = singlet, d = doublet, t = triplet, q = quartet, m = multiplet, br s = broad signal), coupling constant and assignment. Data for ^{13}C NMR are reported in terms of chemical shift (δ ppm). ^{19}F NMR spectra are internally referenced to fluoroform (376 MHz) or trifluoroacetic acid (188 MHz). Mass spectra and conversions of the reactions were recorded on a Shimadzu® GCMS-QP2010 Plus Gas Chromatograph Mass Spectrometer utilizing a MEGA® column (MEGA-5, F.T.: 0.25 μm, I.D.: 0.25 mm, L: 30 m, T_{max}: 350 °C, Column ID# 11475). All reactions were performed with commercially available solvents or compounds, without any previous manipulation or purification and under no special conditions, unless otherwise stated. The aqueous buffer solution consists of aqueous 0.6 M K_2CO_3 and 4 x 10^{-4} M EDTA disodium salt and the pH determined by calibrated pH-meter. Alkene **1p** was prepared according to literature.[1]

Synthesis of the Catalysts

Catalyst **3a** was prepared as previously described.[2] To a stirring solution of 2-bromoiodobenzene (48.3 mL, 375 mmol, 1.00 eq.) in Et_3N (316 mL), copper (I) iodide (0.71 g, 3.75 mmol, 0.01 eq.) was added and the reaction mixture was degassed with argon for 15 minutes. 3-Butyn-1-ol (37.1 mL, 487 mmol, 1.30 eq.) and tetrakis(triphenylphosphine) palladium (4.33 g, 3.75 mmol, 1.00 eq.) were added at r.t. and the reaction mixture was stirred at reflux for 20 h. The reaction mixture was cooled to r.t., the solvent was removed *in vacuo*, the residue was suspended in EtOAc (400 mL) and filtered through a plug of silica gel. The solvent was removed *in vacuo*. The crude mixture was used in next step without further purification.

To a suspension of $Ni(OAc)_2{\cdot}4H_2O$ (24.9 g, 100 mmol, 1.00 eq.) and the above crude mixture in EtOH (300 mL) at r.t., a solution of $NaBH_4$ (90 wt%, 3.80 g, 100 mmol, 1.00 eq.) in EtOH (150 mL) was added dropwise. The black reaction mixture was stirred for 1 h under H_2 atmosphere (balloon) and a solution of ethylenediamine (20.2 mL, 300 mmol, 3.00 eq.) and the above crude mixture in EtOH (250 mL) was added dropwise. The reaction mixture was stirred at r.t. for 60 h under H_2 atmosphere and the solvent was removed *in vacuo*. The residue was suspended in EtOAc (50 mL) and filtered through a plug of silica gel. The solvent was removed *in vacuo* and the desired product (*Z*)-4-(2-bromophenyl)but-3-en-1-ol was used in next step without further purification.

(*Z*)-4-(2-bromophenyl)but-3-en-1-ol (30.0 mmol, 1.50 eq.) was dissolved in dry THF (59 mL) and cooled to 0 °C. *n*Bu_2Mg (0.5 M in heptane, 31.2 mL, 1.04 eq.) was added over 10 min and the resulting reaction mixture was stirred at 0 °C for 30 min. *n*BuLi (1.6 M in hexanes, 2.10 eq.) was added dropwise to the solution and stirred at 0 °C for 45 min. A solution of 1-bromo-2-naphthaldehyde (4.7 g, 20.0 mmol, 1.00 eq.) in dry THF (280 mL) was added dropwise at 0 °C and the reaction mixture was stirred for 25 min at r.t.. The reaction mixture was quenched with aq. sat. NH_4Cl (160 mL) and the organic solvents were removed under reduced pressure. EtOAc (200 mL) was added, the layers were separated and the organic layer was washed with H_2O (100 mL). The organic layer was dried over Na_2SO_4, filtered and the solvent was removed *in vacuo*. (*Z*)-4-(2-((1-

Bromonaphthalen-2-yl)(hydroxy)methyl)phenyl)but-3-en-1-ol was used in next step without further purification.

IBX (10.9 g, 39.1 mmol, 3.00 eq.) was added to a suspension of (*Z*)-4-(2-((1-bromonaphthalen-2-yl)(hydroxy)methyl)phenyl)but-3-en-1-ol (13.0 mmol, 1.00 eq.) in MeCN (650 mL) and the suspension was vigorously stirred at 50 °C for 4 h. The reaction mixture was cooled to r.t., filtered and concentrated *in vacuo*. $CHCl_3$ (2 L) was added to the residue and the reaction mixture was filtered to yield a solution of the keto-aldehyde, which was directly used in the next step.

To the above keto-aldehyde solution, (*S*)-5-(2-pyrrolidinyl)-1*H*-tetrazole (452 mg, 3.25 mmol, 0.25 eq.) in DMF (217 mL) and H_2O (650 mL) was added and the reaction mixture was vigorously stirred at r.t. for 17 h. The layers were separated and the aqueous layer was extracted with CH_2Cl_2 (3 x 300 mL). The combined organic layers were concentrated at 55 °C. To remove the remaining DMF, toluene (500 mL) was added and the solution was concentrated again. The crude aldol addition product was dissolved in $CHCl_3$ (620 mL) and Amberlite® IRA-96 (24.0 g, washed with 2 x MeOH and 2 x CH_2Cl_2) was added. The reaction mixture was stirred for 4 h at r.t., filtered, dried over Na_2SO_4, filtered again and concentrated to give (*S*)-1'-bromo-[1,2'-binaphthalene]-2-carbaldehyde, which was used in next step without further purification.

(*S*)-1'-bromo-[1,2'-binaphthalene]-2-carbaldehyde (3.30 mmol, 1.00 eq.), was treated with (*Z*)-4-(2-bromophenyl)but-3-en-1-ol (1.13 g, 5.00 mmol, 1.50 eq.), *n*-Bu_2Mg (0.5 M, 5.2 mL, 2.60 mmol, 0.78 eq.) and *n*-BuLi (1.6 M, 3.1 mL, 5.00 mmol, 1.5 eq.) following the same procedure as described above. (*Z*,*R*,*S*)-4-(1'-Bromo-([1:2']-binaphthalen-2-yl(hydroxy)methyl)phenyl)but-3-en-1-ol (1.34 mmol, 1.00 eq.) was obtained and the double oxidation procedure was followed as described above utilizing IBX (1.12 g, 4.02 mmol, 3.00 eq.) as the oxidant and MeCN (67 mL) as the solvent. To the keto-aldehyde solution (1.40 mmol, 0.02 M in $CHCl_3$) at r.t., *N*-benzylcinchonidinium chloride (5.89 mg, 0.01 mmol, 0.01 eq.) was added, followed by aq. KOH (1.00 M, 28 mL). The reaction mixture was stirred for 16 h at r.t., aq. sat. NH_4Cl (60 mL) and H_2O (60 mL) were added, the layers were separated and the aqueous layer was extracted with CH_2Cl_2 (3 x 50 mL). The combined organic layers were dried over Na_2SO_4, filtered and concentrated *in vacuo*. Purification by column chromatography (cyclohexane:CH_2Cl_2 4:1,

R_f 0.10) gave the aldol condensation product **3a** as colorless solid. All data are in agreement with literature[1] (640 mg, d.r. = 92:8); m.p. 238-240 °C; $[\alpha]^D$ +476 (*c* 0.25 in $CHCl_3$); **^{1}H NMR** (400 MHz, $CDCl_3$): δ 10.28 (1H, s, CHO), 8.27 (1H, d, *J* = 8.5 Hz, ArH), 8.14 (1H, d, *J* = 8.4 Hz, ArH), 8.10 (1H, d, *J* = 8.2 Hz, ArH), 7.87 (1H, d, *J* = 8.6 Hz, ArH), 7.82 (1H, d, *J* = 8.1 Hz, ArH), 7.72 (1H, d, *J* = 8.7 Hz, ArH), 7.67 (1H, d, *J* = 8.9 Hz, ArH), 7.64-7.45 (9H, m, ArH), 7.40 (1H, d, *J* = 8.5 Hz, ArH), 7.34-7.28 (1H, m, ArH); **^{13}C NMR** (100 MHz, $CDCl_3$): δ = 192.5, 145.5, 139.8, 136.6, 135.7, 133.5, 133.2, 132.9, 132.4, 132.0, 131.9, 131.3, 128.5, 128.3, 128.3, 128.2, 128.1, 128.0, 127.8, 127.7, 127.5, 127.1, 126.8, 126.7, 126.6, 126.6, 126.5, 124.6, 122.0; **HRMS** exact mass calculated for $[M+Na]^+$ ($C_{31}H_{19}BrNaO^+$) requires *m/z* 509.0511, found *m/z* 509.0508.

Catalysts **3b-3e** were available from a previous study.[2] Compounds **3f-3i** are commercially available.

Catalyst Evaluation

a-Methyl-styrene **1a** (24 mg, 0.20 mmol) was placed in a round bottom flask followed by the specified catalyst (0.01 mmol). *tert*-Butanol (0.2 mL), aqueous buffer solution (0.2 mL, 0.6 M K_2CO_3 – 4 x 10^{-4} M EDTA disodium salt), acetonitrile (0.02 mL, 0.40 mmol) and 30% aqueous H_2O_2 (0.04 mL, 0.40 mmol) were added consecutively. The reaction mixture was stirred for 5 h at room temperature. H_2O (0.4 mL) and $CHCl_3$ (2 mL) were added in the reaction mixture, the layers were separated and the aqueous layer was extracted with $CHCl_3$ (3 x 2 mL). The combined organic layers were dried over Na_2SO_4, filtered and concentrated *in vacuo*. The yield was determined by ^{1}H-NMR using an internal standard.

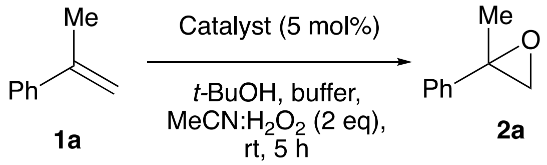

1a **2a**

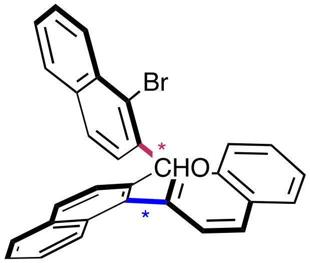

3a (74%)

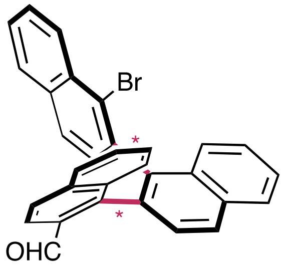

3b (34%)

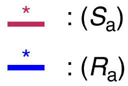

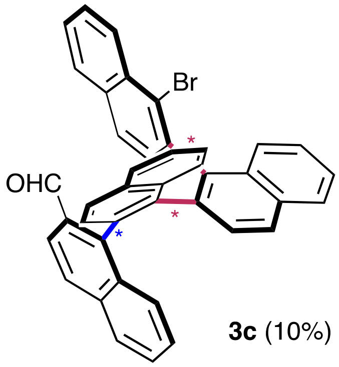

3c (10%)

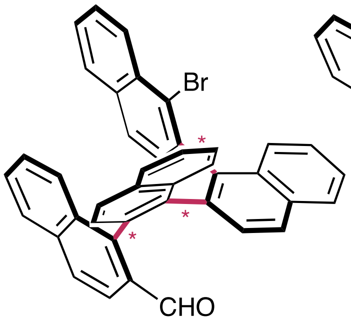

3d (17%)

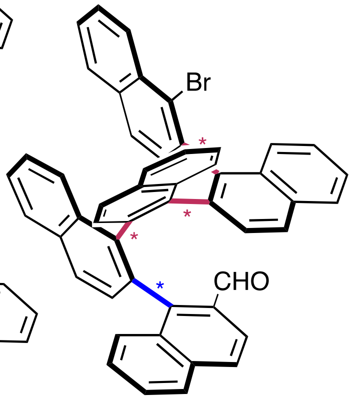

3e (5%)

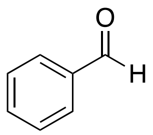

3f (34%)

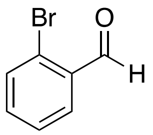

3g (40%)

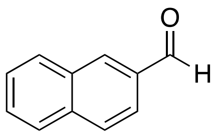

3h (18%)

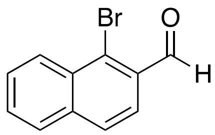

3i (48%)

Optimization of the Aldehyde-Catalyzed Epoxidation

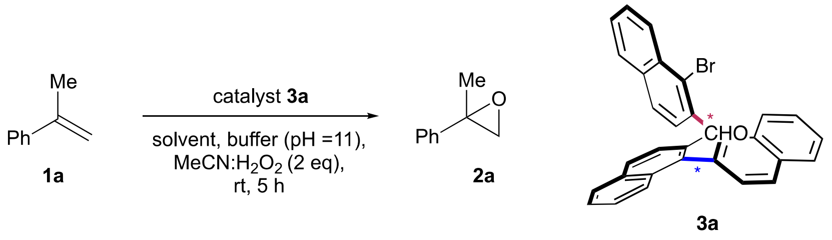

Entry	Catalyst 3a (mol%)	Oxidant (equiv.)	Buffer (pH)	Solvent	Yield (%)[a]	*ee* (%)[b]
1	5	H_2O_2 (6)	11	tBuOH	98	6
2	5	H_2O_2 (2)	11	tBuOH	74	6
3	-	H_2O_2 (6)	11	tBuOH	14	-
4	-	H_2O_2 (2)	11	tBuOH	9	-
5	5	-	11	tBuOH	0	-
6	5	H_2O_2 (2)	-	tBuOH	0	-
7[c]	5	H_2O_2 (2)	11	tBuOH	0	-
8	5	H_2O_2 (2)	10	tBuOH	41	2
9	1	H_2O_2 (2)	11	tBuOH	48	5
**10**	**1**	**H_2O_2 (2)**	**11**	**MeOH**	**83**	**6**
**11**	**5**	**H_2O_2 (2)**	**11**	**MeOH**	**100**	**4**
12	1	H_2O_2 (2)	11	MeCN	82	8
13	1	H_2O_2 (2)	11	EtOAc	67	4
14	1	H_2O_2 (2)	11	CH_2Cl_2	15	4
15	1	H_2O_2 (2)	11	$CHCl_3$	12	10
16	1	H_2O_2 (2)	11	THF	36	4
17	1	H_2O_2 (2)	11	Et_2O	0	-
18[d]	5	Oxone (2)	8	MeCN/DME	65	-

[a] Yield was determined by ^{1}H-NMR using internal standard. The reaction was performed with *a*-methyl-styrene **1a** (24 mg, 0.20 mmol), Catalyst **3a** (0-0.01 mmol), *tert*-butanol (0.2 mL), aqueous buffer solution (0.2 mL, 0.6M K_2CO_3 – 4 x 10^{-4} M EDTA disodium salt), acetonitrile (0.40-1.20 mmol) and 30% aqueous H_2O_2 (0.40-1.20 mmol) for 5 h. [b] Chiralcel OD-H column (1.0 mL/min, i-PrOH:*n*-heptane 5:95): (*R*) t_R = 15.1 min and (*S*) t_R = 17.0 min. [c] No MeCN. [d] The reaction was performed with *a*-methyl-styrene **1a** (24 mg, 0.20 mmol), catalyst **3a** (0.01 mmol), MeCN (1 mL), DME (2 mL), aqueous buffer solution (2 mL, pH=8.0, 0.1 M K_2CO_3-AcOH), Oxone (250 mg, 0.40 mmol) were dissolved in aqueous solution of EDTA disodium salt (1.3 mL, 5 x 10^{-4} M) and aqueous solution of K_2CO_3 (1.3 mL, 0.9 M) for 5 h.

General Procedure and Substrate Scope

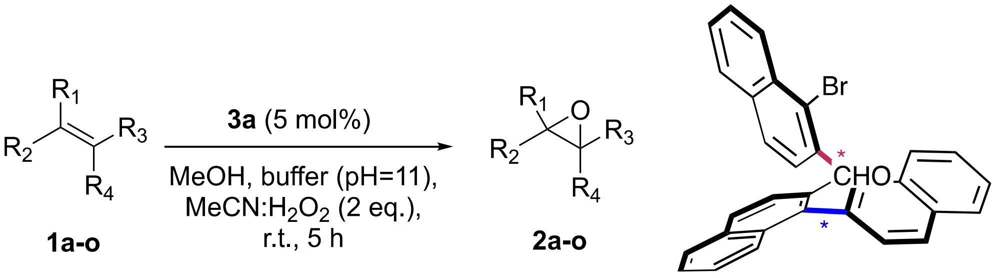

3a

Alkene (0.20 mmol) was placed in a round bottom flask followed by catalyst **3a** (4.9 mg, 0.01 mmol). Methanol (0.2 mL), aqueous buffer solution (0.2 mL, 0.6M K_2CO_3 – 4 x 10^{-4} M EDTA disodium salt), acetonitrile (0.02 mL, 0.40 mmol) and 30% aqueous H_2O_2 (0.04 mL, 0.40 mmol) were added consecutively. The reaction mixture was left stirring for 5-18 h depending on the substrate. The crude product was purified using flash column chromatography (10%-20% EtOAc in Pet. Ether) to afford the desired epoxide.

2-Methyl-2-phenyloxirane (2a)[3]

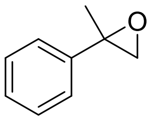

Colorless oil (99% yield); **^{1}H NMR** ($CDCl_3$) δ: 7.44-7.08 (5H, m, ArH), 2.98 (1H, d, J = 5.3 Hz, OC*H*H), 2.80 (1H, d, J = 5.3 Hz, OCH*H*), 1.73 (3H, s, CH_3); **^{13}C NMR** ($CDCl_3$) δ: 141.1, 128.2, 127.3, 125.2, 56.9, 56.6, 21.7; **MS (ESI) m/z (%)** 135 [M+H, 100%]$^+$.

2-Phenyloxirane (2b)[3]

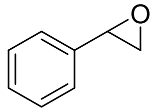

Colorless oil (97% yield); **^{1}H NMR** ($CDCl_3$) δ: 7.36-7.27 (5H, m, ArH), 3.87 (1H, dd, *J* = 4.1 and 2.6 Hz, OCH), 3.15 (1H, dd, *J* = 5.5 and 4.1 Hz, OC*H*H), 2.81 (1H, dd, *J* = 5.5 and 2.6 Hz, OCH*H*); **^{13}C NMR** (400 MHz, $CDCl_3$) δ: 137.6, 128.5, 128.2, 125.5, 52.3, 51.2; **MS (ESI) m/z (%)** 121 [M+H, 100%]$^+$.

2-(4-Methoxyphenyl)oxirane (2c)[4]

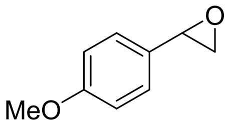

Colorless oil (99% yield); **^{1}H NMR** ($CDCl_3$) δ: 7.20 (2H, d, *J* = 8.7 Hz, ArH), 6.89 (2H, d, *J* = 8.7 Hz, ArH), 3.82 (1H, dd, *J* = 4.1 and 2.7 Hz, OCH), 3.80 (3H, s, OCH_3), 3.12 (1H, dd, *J* = 5.2 and 4.1 Hz, OC*H*H), 2.80 (1H, dd, *J* = 5.2 and 2.7 Hz, OCH*H*); **^{13}C NMR** (400 MHz, $CDCl_3$) δ: 159.6, 129.4, 126.8, 113.9, 55.2, 52.1, 50.9; **MS (ESI) m/z (%)** 151 [M+H, 100%]$^+$.

2-(4-Bromophenyl)oxirane (2d)[3]

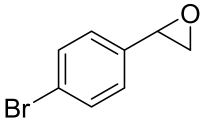

Colorless oil (32% yield); **^{1}H NMR** ($CDCl_3$) δ: 7.45 (2H, d, *J* = 8.4 Hz, ArH), 7.15 (2H, d, *J* = 8.4 Hz, ArH), 3.81 (1H, dd, *J* = 4.1 and 2.6 Hz, OCH), 3.12 (1H, dd, *J* = 5.5 and

4.1 Hz, OC*H*H), 2.73 (1H, dd, *J* = 5.5 and 2.6 Hz, OCH*H*); **^{13}C NMR** ($CDCl_3$) *δ*: 136.7, 131.6, 127.1, 122.0, 51.8, 51.2; **MS (ESI) m/z (%)** 199 [M+H, 100%]$^+$.

2-(*p*-Tolyl)oxirane (2e)[3]

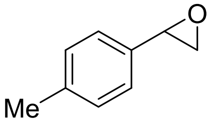

Colorless oil (31% yield); **^{1}H NMR** ($CDCl_3$) *δ*: 7.38-6.91 (4H, m, ArH), 3.82 (1H, dd, *J* = 4.1 and 2.5 Hz, OCH), 3.12 (1H, dd, *J* = 5.5 and 4.1 Hz, OC*H*H), 2.78 (1H, dd, *J* = 5.5 and 2.5 Hz, OCH*H*), 2.33 (3H, s, CH_3); **^{13}C NMR** ($CDCl_3$) *δ*: 138.0, 134.5, 129.2, 125.4, 52.3, 51.2, 21.2; **MS (ESI) m/z (%)** 135 [M+H, 100%]$^+$.

2-Phenethyloxirane (2f)[5]

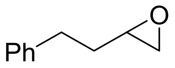

Colorless oil (64% yield); **^{1}H NMR** ($CDCl_3$) *δ*: 7.58-7.13 (5H, m, ArH), 3.00-2.92 (1H, m, OCH), 2.87-2.70 (3H, m, $PhCH_2$ and OC*H*H), 2.48 (1H, dd, *J* = 5.0 and 2.7 Hz, OCH*H*), 1.95-1.78 (2H, m, CH_2); **^{13}C NMR** ($CDCl_3$) *δ*: 141.1, 128.3, 128.2, 125.9, 51.6, 47.1, 34.2, 32.1; **MS (ESI) m/z (%)** 149 [M+H, 100%]$^+$.

2-Octyloxirane (2g)[3]

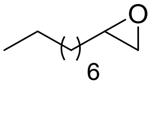

Colorless oil (69% yield); **^{1}H NMR** ($CDCl_3$) *δ*: 2.89-2.82 (1H, m, OCH), 2.71 (1H, m, OC*H*H), 2.42 (1H, dd, *J* = 5.0 and 2.7 Hz, OCH*H*), 1.63-1.06 (14H, m, 7 x CH_2), 0.84

(3H, t, J = 7.0 Hz, CH_3); **^{13}C NMR** ($CDCl_3$) δ: 52.4, 47.0, 32.4, 31.8, 29.4, 29.3, 29.1, 25.9, 22.6, 14.0; **MS (ESI) m/z (%)** 87 [M+H, 100%]$^+$.

2-Methyl-3-phenyloxirane (2h)[3]

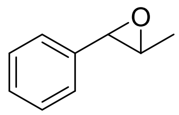

Colorless oil (99% yield); Mixture of *trans*:*cis* = 99:1, from olefin **1h** (*trans*:*cis* = 99:1); **^{1}H NMR** ($CDCl_3$) δ: 7.47-6.90 (5H, m, ArH), 3.58 (1H, d, J = 2.1 Hz, OCH), 3.04 (1H, qd, J = 5.1 and 2.1 Hz, OCH), 1.46 (3H, d, J = 5.1 Hz, CH_3); **^{13}C NMR** ($CDCl_3$) δ: 137.7, 128.4, 128.0, 125.5, 59.5, 59.0, 17.9; **MS (ESI) m/z (%)** 135 [M+H, 100%]$^+$.

(3-Phenyloxiran-2-yl)methanol (2i)[3]

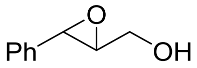

Colorless oil (96% yield); 100% *trans*, from olefin **1i** (100% *trans*); **^{1}H NMR** ($CDCl_3$) δ: 7.80-7.23 (5H, m, ArH), 4.02 (1H, dd, J = 12.8 and 2.4 Hz, OC*H*H), 3.91 (1H, d, J = 2.4 Hz, PhCH), 3.75 (1H, dd, J = 12.8 and 4.2 Hz, OCH*H*), 3.21 (1H, dt, J = 4.2 and 2.4 Hz, OCH), 2.42 (1H, br s, OH); **^{13}C NMR** ($CDCl_3$) δ: 136.5, 128.4, 128.2, 125.6, 62.5, 61.2, 55.6; **MS (ESI) m/z (%)** 151 [M+H, 100%]$^+$.

6-Oxabicyclo[3.1.0]hexane (2j)[6]

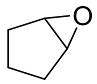

Colorless oil (96% yield); **^{1}H NMR** ($CDCl_3$) δ: 2.11-1.92 (2H, m, 2 x OCH), 1.68-1.21 (6H, m, 3 x CH_2); **^{13}C NMR** ($CDCl_3$) δ: 57.1, 29.1, 19.7; **MS (ESI) m/z (%)** 85 [M+H, 100%]$^+$.

7-Oxabicyclo[4.1.0]heptane (2k)[3]

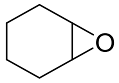

Colorless oil (97% yield); **^{1}H NMR** ($CDCl_3$) δ: 3.16-3.10 (2H, m, 2 x OCH), 1.97-1.77 (4H, m, 2 x CH_2), 1.48-1.35 (2H, m, CH_2), 1.28-1.07 (2H, m, CH_2); **^{13}C NMR** ($CDCl_3$) δ: 52.6, 24.9, 19.8; **MS (ESI) m/z (%)** 99 [M+H, 100%]$^+$.

8-Oxabicyclo[5.1.0]octane (2l)[3]

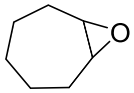

Colorless oil (77% yield); **^{1}H NMR** ($CDCl_3$) δ: 3.07-3.04 (2H, m, 2 x OCH), 1.93-1.87 (4H, m, 2 x CH_2), 1.58-132 (6H, m, 3 x CH_2); **^{13}C NMR** ($CDCl_3$) δ: 56.3, 31.3, 29.2, 24.7; **MS (ESI) m/z (%)** 113 [M+H, 100%]$^+$.

9-Oxabicyclo[6.1.0]nonane (2m)[3]

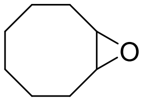

Colorless oil (72% yield); **^{1}H NMR** ($CDCl_3$) *δ*: 3.01-2.76 (2H, m, 2 x OCH), 2.33-1.96 (2H, m, CH_2), 1.69-1.02 (10H, m, 5 x CH_2); **^{13}C NMR** ($CDCl_3$) *δ*: 55.5, 26.4, 26.2, 25.5; **MS (ESI) m/z (%)** 127 [M+H, 100%]$^+$.

1-Methyl-4-(2-methyloxiran-2-yl)-7-oxabicyclo[4.1.0]heptane (2n)[3]

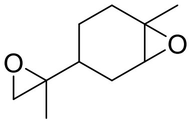

Colorless oil (68% yield); 1:1:1:1 mixture of diastereomers; **^{1}H NMR** ($CDCl_3$) *δ*: 3.05-2.93 (1H, m, OCH), 2.64-2.45 (2H, m, OCH_2), 2.14-1.39 (7H, m, 3 x CH_2 and CH), 1.27 (3H, s, CH_3), 1.19-1.14 (3H, m, CH_3); **^{13}C NMR** ($CDCl_3$) *δ*: 60.3, 59.9, 59.0, 58.7, 58.6, 58.5, 57.7, 57.6, 57.3, 57.2, 53.3, 53.1, 52.8, 52.6, 39.8, 39.2, 36.2, 35.3, 34.7, 30.1, 30.0, 28.6, 28.5, 28.3, 27.6, 26.5, 26.3, 24.3, 24.2, 23.5, 23.2, 22.9, 22.8, 21.2, 21.1, 18.6, 18.1, 18.0, 17.3; **MS (ESI) m/z (%)** 169 [M+H, 100%]$^+$.

1-Phenyl-7-oxabicyclo[4.1.0]heptane (2o)[3]

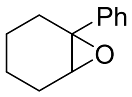

Colorless oil (46% yield); **^{1}H NMR** ($CDCl_3$) *δ*: 7.55-7.15 (5H, m, ArH), 3.09-3.08 (1H, m, OCH), 2.49-1.89 (4H, m, 2 x CH_2), 1.70-1.25 (4H, m, 2 x CH_2); **^{13}C NMR** ($CDCl_3$) *δ*: 142.3, 128.0, 127.0, 125.1, 61.7, 60.0, 28.6, 24.5, 19.9, 19.6; **MS (ESI) m/z (%)** 175 [M+H, 100%]$^+$.

Control Experiments and Radical Clock Experiment

Control Experiments

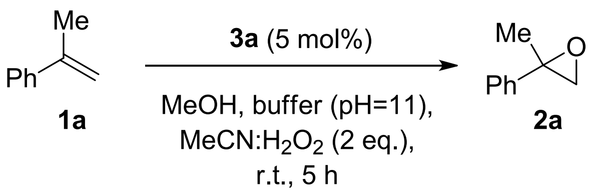

Deviation from Standart Conditions	Yield (%)
none	99%
No O_2 (under Ar)	11%
No O_2 + 2 mol% mCPBA	42%
open air	96%
under O_2 (1 Atm)	95%
TEMPO (1 equiv)	0%
BHT (1 equiv)	8%
in the dark	75%
acid of **3a** instead of **3a**	12%
PhCOOH instead of **3a**	11%

Standard Conditions: Alkene **1a** (24 mg, 0.20 mmol) was placed in a round bottom flask followed by catalyst **3a** (4.9 mg, 0.01 mmol). Methanol (0.2 mL), aqueous buffer solution (0.2 mL, 0.6M K_2CO_3 – 4 x 10^{-4} M EDTA disodium salt), acetonitrile (0.02 mL, 0.40 mmol) and 30% aqueous H_2O_2 (0.04 mL, 0.40 mmol) were added consecutively. The reaction mixture was left stirring for 5 h.

No O_2 (under Ar): All reagents and solvents were bubbling with N_2 for 20 minutes, before reaction set-up. After set-up of the reaction, the reaction mixture was degassed and a balloon of Ar was used.

No O_2 + 2 mol% mCPBA: All reagents and solvents were bubbling with N_2 for 20 minutes, before reaction set-up. After set-up of the reaction, the reaction mixture was degassed and a balloon of Ar was used. In the reaction mixture, *m*CPBA (2 mol%) was added. The same reaction was performed utilizing 2-bromobenzaldehyde as the catalyst and the desired epoxide was formed in 37% yield (yield was determined by ^{1}H-NMR using internal standard).

Open air: The reaction mixture was left stirring without a cap.

under O_2: After set-up of the reaction, the reaction mixture was degassed and a balloon of O_2 was used.

TEMPO: In the reaction mixture, 2,2,6,6-tetramethylpiperidinooxy (31.2 mg, 0.20 mmol) was added.

BHT: In the reaction mixture, 2,6-di-*tert*-butyl-4-methylphenol (44 mg, 0.20 mmol) was added.

In the dark: The reaction mixture was left stirring for 5 h in the dark.

Acid of **3a** instead of **3a**: The acid was **3a** was used as the catalyst, instead of **3a**.

PhCOOH instead of **3a**: PhCOOH was used as the catalyst, instead of **3a**.

Radical Clock Experiment

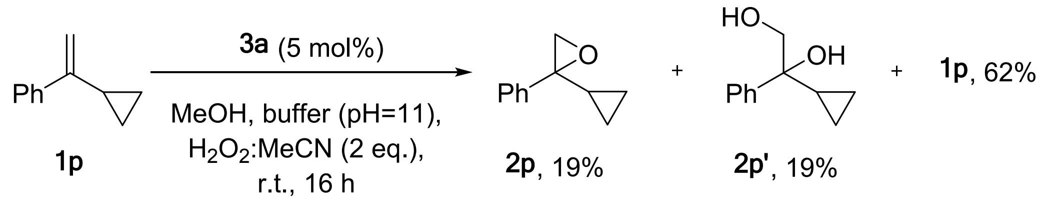

Alkene **1p** (29 mg, 0.20 mmol) was placed in a round bottom flask followed by catalyst **3a** (4.9 mg, 0.01 mmol). Methanol (0.2 mL), aqueous buffer solution (0.2 mL, 0.6M K_2CO_3 – 4 x 10^{-4} M EDTA disodium salt), acetonitrile (0.02 mL, 0.40 mmol) and 30% aqueous H_2O_2 (0.04 mL, 0.40 mmol) were added consecutively. The reaction mixture was left stirring for 5 h at room temperature. The reaction mixture was filled with chloroform (5 mL), dried over Na_2SO_4, filtered and the solvent was removed *in vacuo*. The ^{1}H-NMR spectrum of the crude reaction mixture is presented below (**2p**: 2.87 ppm,

1H, d and 2.75 ppm, 1H, d, *J* = 5.4 Hz, **2p'**: 3.96 ppm, 1H, d and 3.80 ppm, 1H, d, *J* = 11.2 Hz, **1p**: 5.27 ppm, 1H, s and 4.93 ppm, 1H, s).[7]

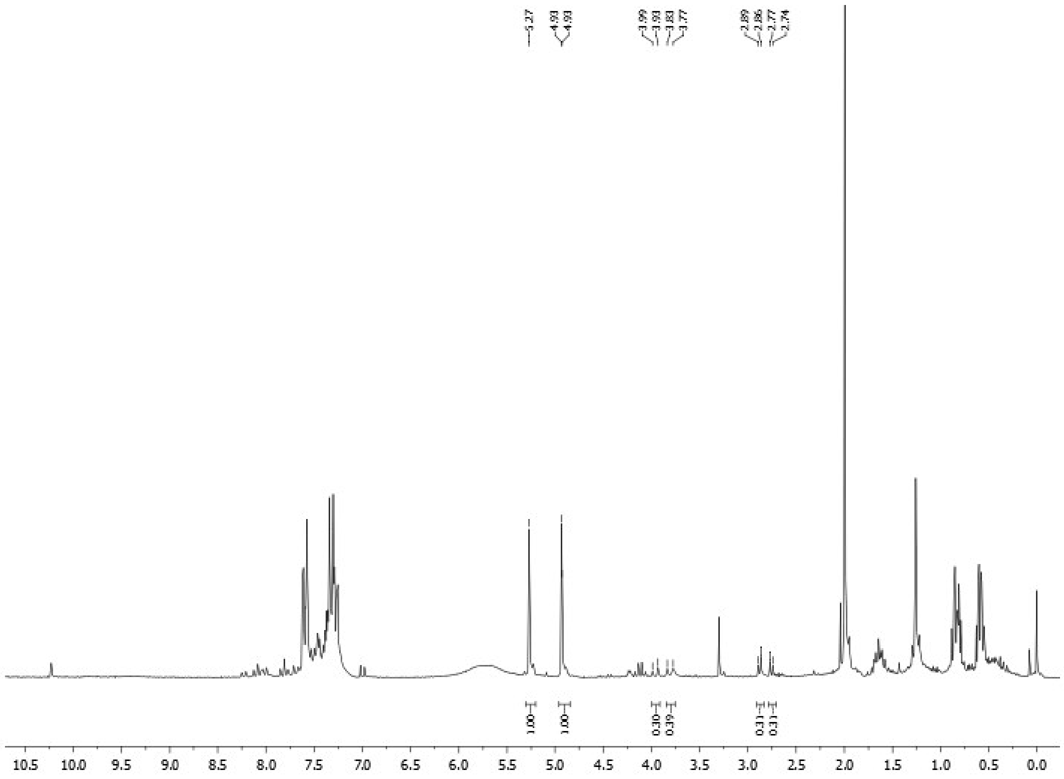

High Resolution Mass Spectrometry Studies

Instrumentation

The High Resolution Mass Spectra were recorded with a Q-TOF (Time of Flight Mass Spectrometry) Bruker Maxis Impact with ESI source coupled with U-HPLC Thermo Dionex Ultimate 3000 pump and autosampler. N_2 was used as collision gas and electrospray ionization (ESI) – positive or negative mode - was used for the MS experiments. The data acquisition was carried out with Data Analysis from Bruker Daltonics (version 4.1). For the MS experiments, a solution approximately of 10 mg/L in acetonitrile from the reaction mixture was used. Acetonitrile LC-MS gradient was obtained from Carlo Erba Reagents (Chaussée du Vexin, France). (Source conditions: End plate offset 500V, Capillary 4500V, Nebulizer 0.4 bar, dry gas 4.0 L/min, dry temperature 180 °C and Quadrupole conditions: Ion energy 5 eV, Collision energy 10 eV, Transfer time 143 μs, Collision ion RF 3500 vpp, Pre pulse storage 1μs).

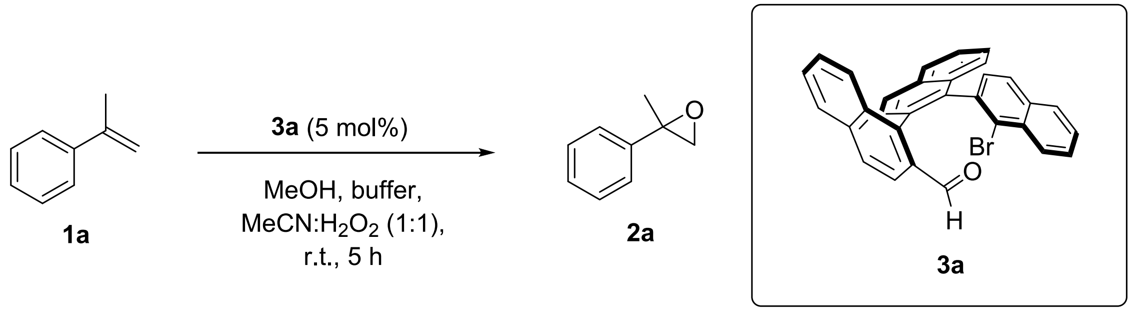

a-Methyl-styrene (24 mg, 0.20 mmol) was placed in a round bottom flask followed by catalyst **3a** (4.9 mg, 0.01 mmol). Methanol (0.2 mL), aqueous buffer solution (0.2 mL, 0.6M K_2CO_3 – 4 x 10^{-4} M EDTA disodium salt), acetonitrile (0.02 mL, 0.40 mmol) and 30% aqueous H_2O_2 (0.04 mL, 0.40 mmol) were added consecutively. Following the reaction by HRMS and focusing on the possible mechanistic pathway, initially, full scan spectra in both negative and positive mode are presented below:

Negative ESI Mode

0 min

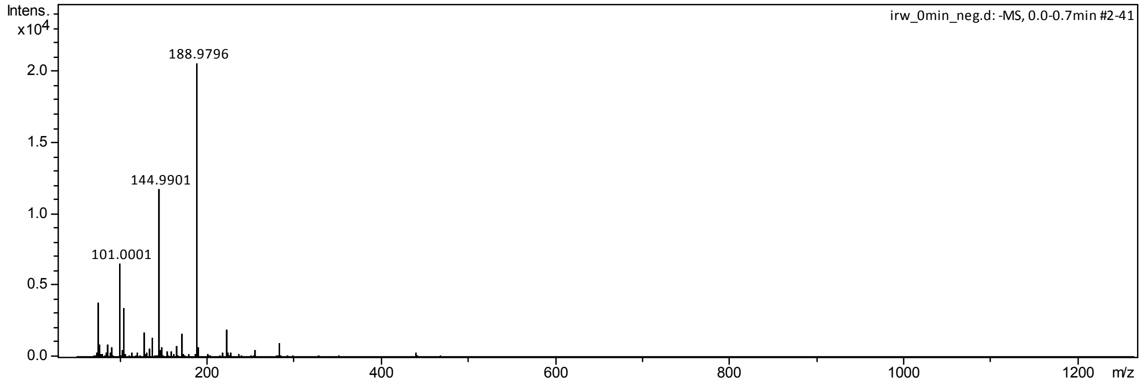

20 min

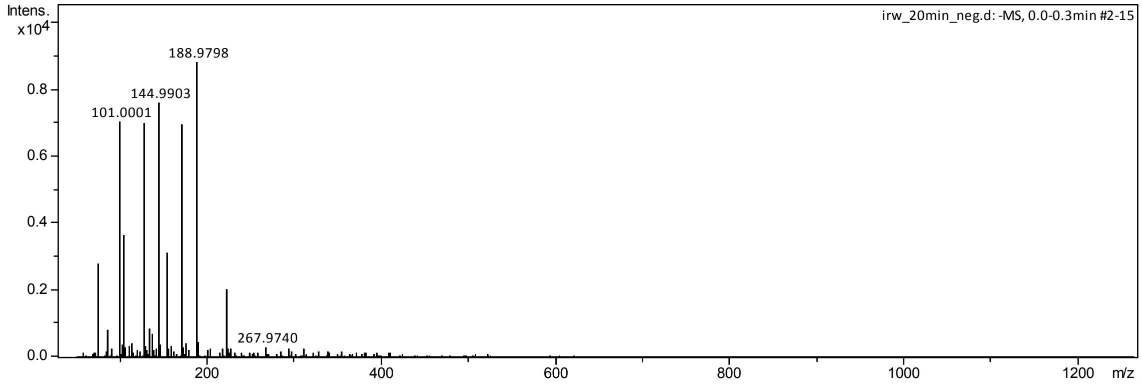

40 min

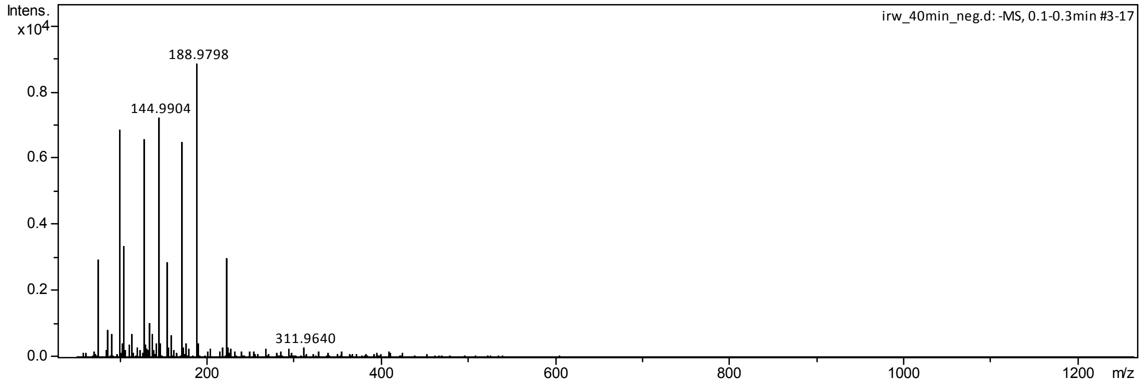

1 h

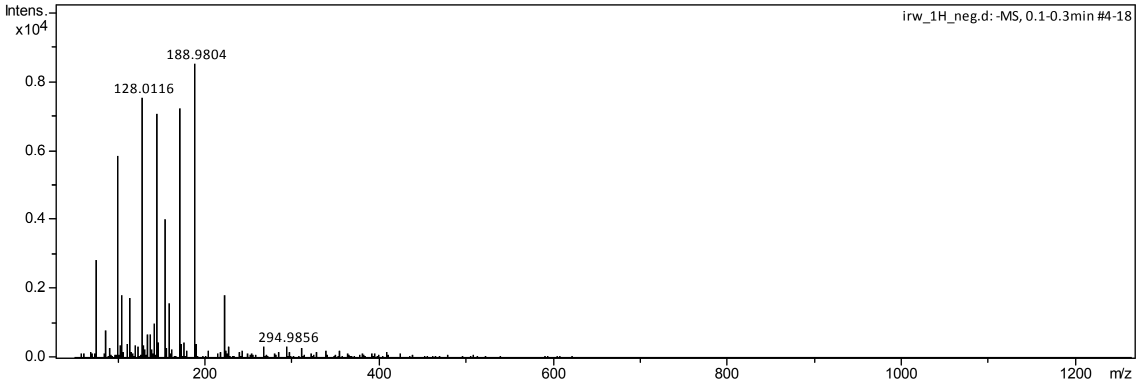
Intens.
x10^4
irw_1H_neg.d: -MS, 0.1-0.3min #4-18
188.9804
128.0116
294.9856
0.8
0.6
0.4
0.2
0.0
200
400
600
800
1000
1200
m/z

2 h

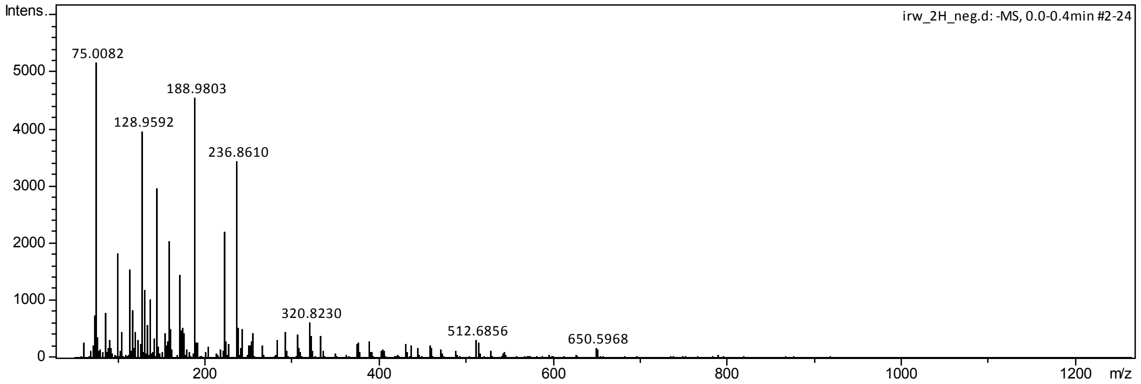
Intens.
irw_2H_neg.d: -MS, 0.0-0.4min #2-24
75.0082
188.9803
128.9592
236.8610
320.8230
512.6856
650.5968
5000
4000
3000
2000
1000
0
200
400
600
800
1000
1200
m/z

3 h

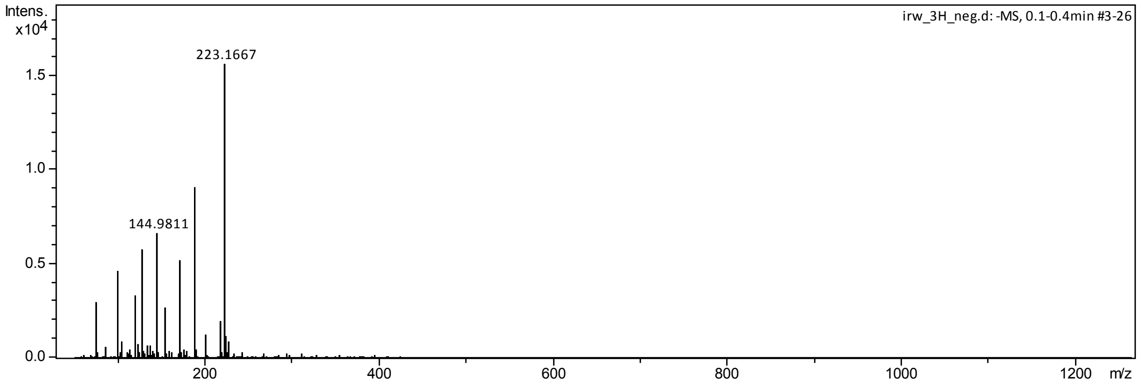
Intens.
x10^4
irw_3H_neg.d: -MS, 0.1-0.4min #3-26
223.1667
144.9811
1.5
1.0
0.5
0.0
200
400
600
800
1000
1200
m/z

4 h

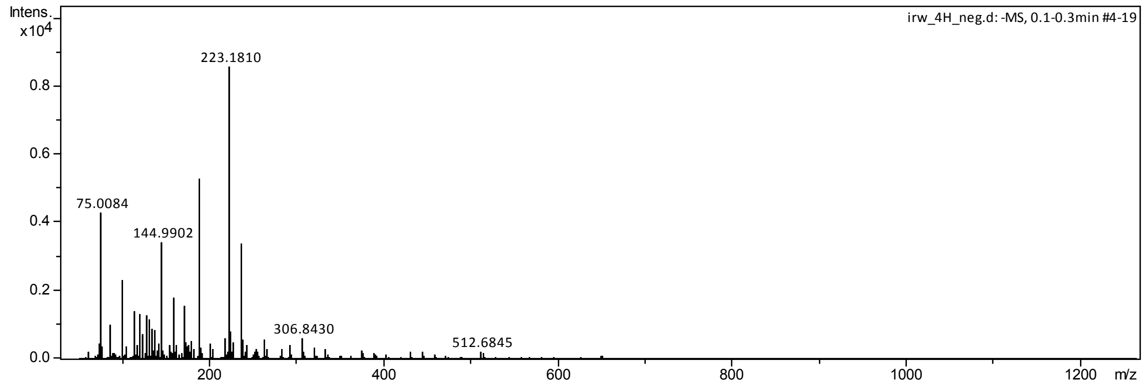
Intens.
x10^4
irw_4H_neg.d: -MS, 0.1-0.3min #4-19
223.1810
75.0084
144.9902
306.8430
512.6845
0.8
0.6
0.4
0.2
0.0
200
400
600
800
1000
1200
m/z

5 h

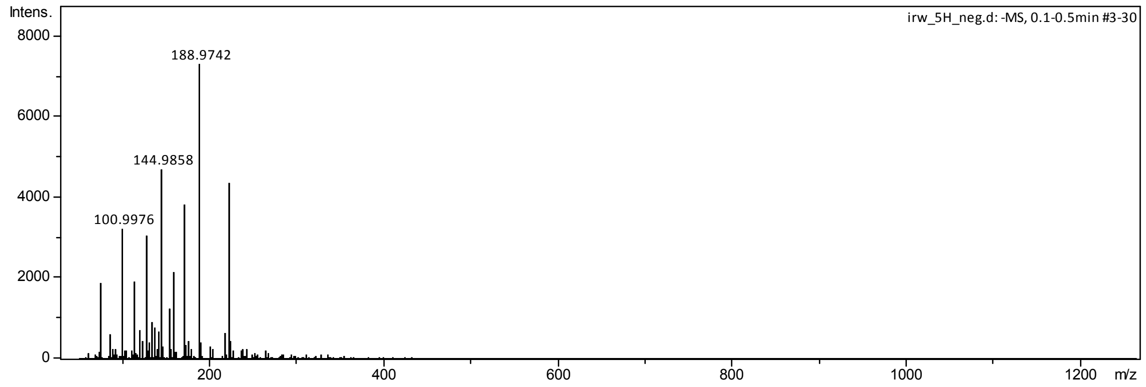
Intens.
irw_5H_neg.d: -MS, 0.1-0.5min #3-30
188.9742
144.9858
100.9976
8000
6000
4000
2000
0
200
400
600
800
1000
1200
m/z

Positive ESI Mode

0 min

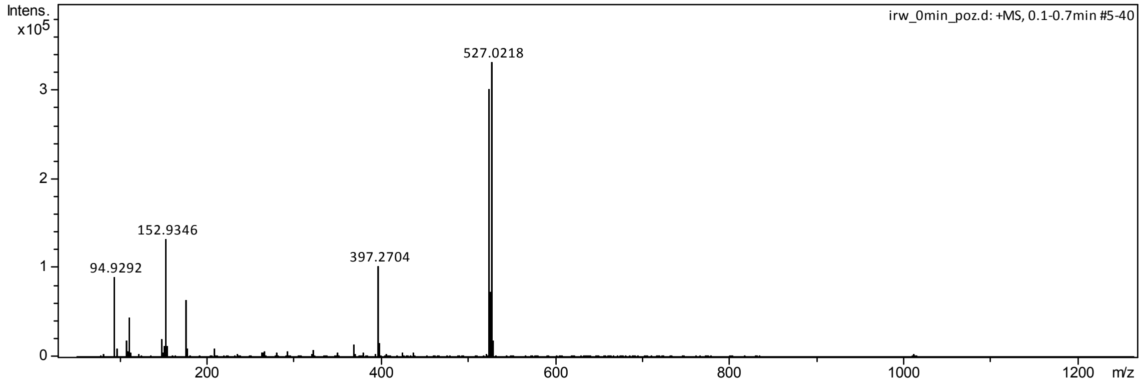

20 min

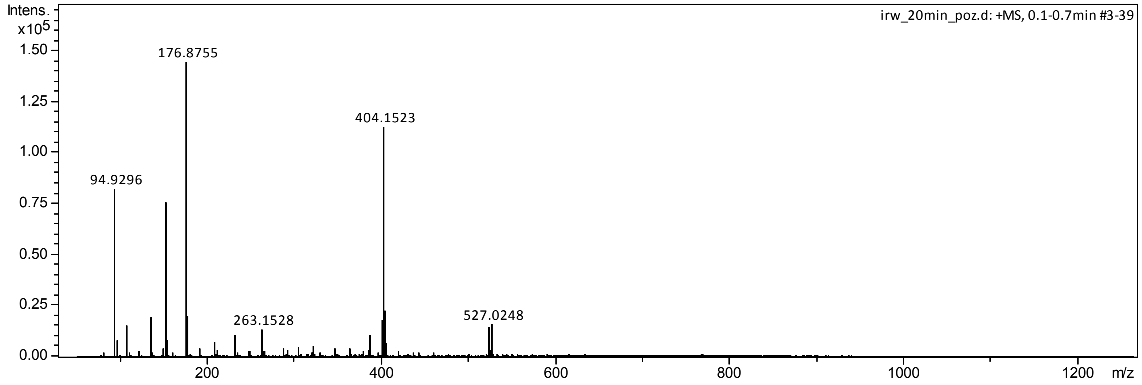

40 min

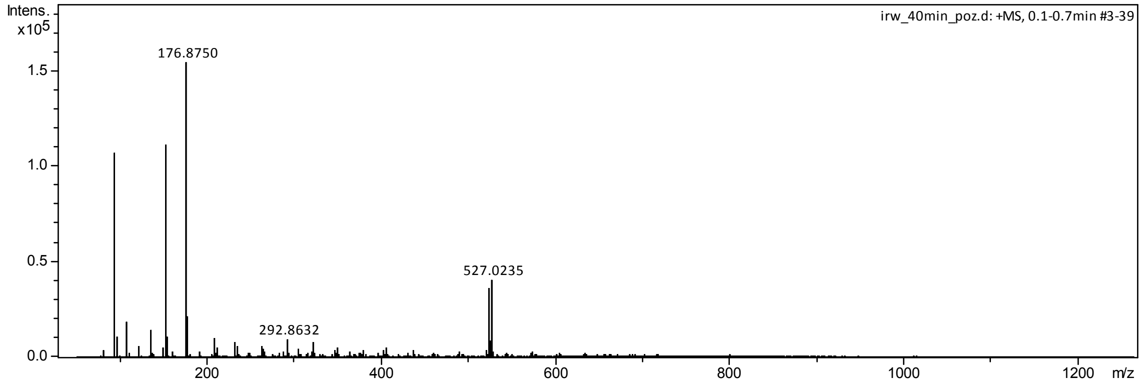

1 h

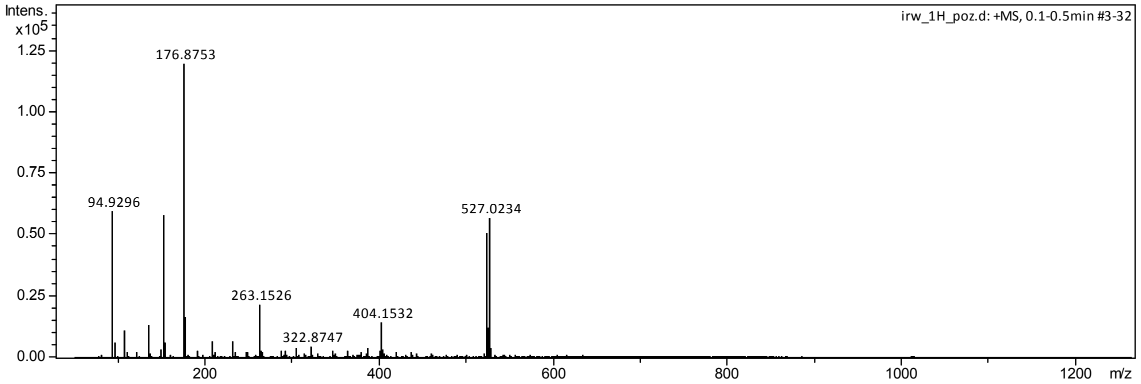
irw_1H_poz.d: +MS, 0.1-0.5min #3-32
Intens. x10^5
176.8753
94.9296
527.0234
263.1526
404.1532
322.8747
m/z

2 h

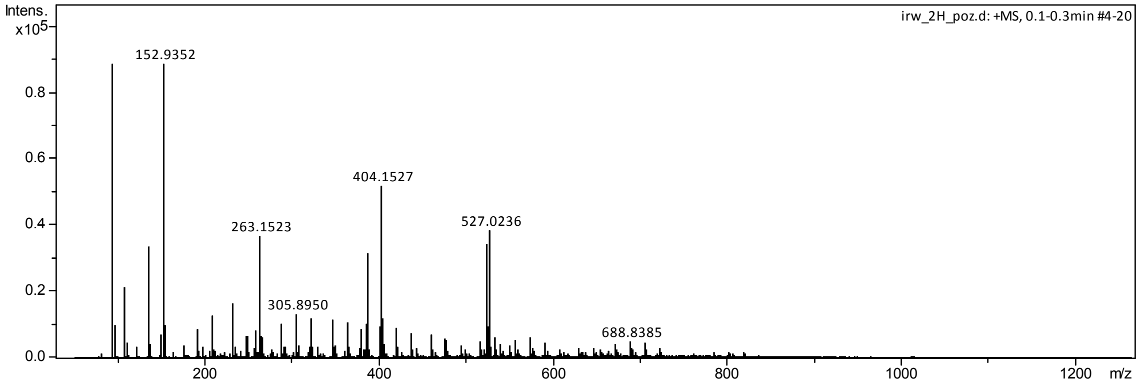
irw_2H_poz.d: +MS, 0.1-0.3min #4-20
Intens. x10^5
152.9352
404.1527
263.1523
527.0236
305.8950
688.8385
m/z

3 h

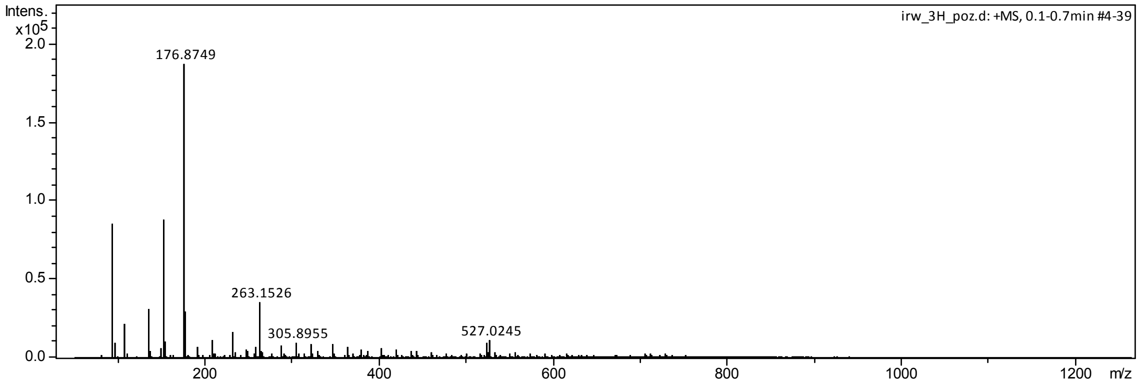
irw_3H_poz.d: +MS, 0.1-0.7min #4-39
Intens. x10^5
176.8749
263.1526
305.8955
527.0245
m/z

4 h

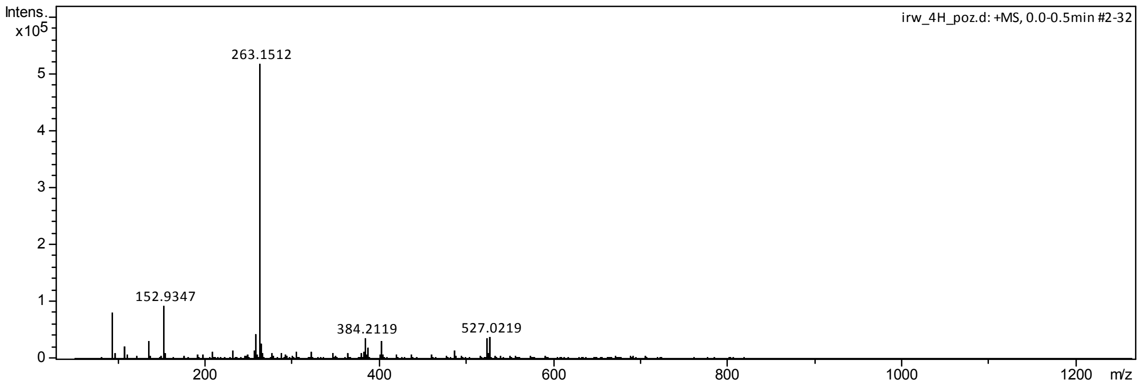

5 h

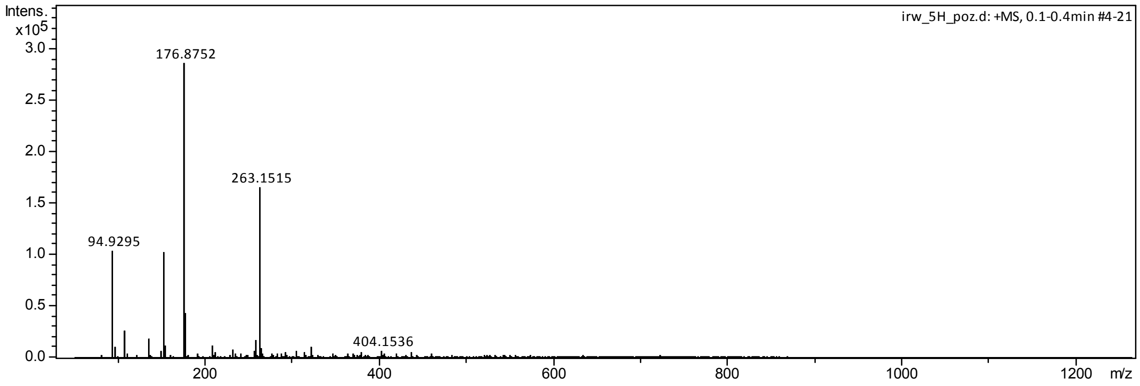

In the following part, the proposed mechanistic pathways of the reaction are presented. Intermediates that were detected are presented below. For the radical intermediates, the corresponding neutral species were also sought, but were also not identified.

Mechanistic Pathway 1

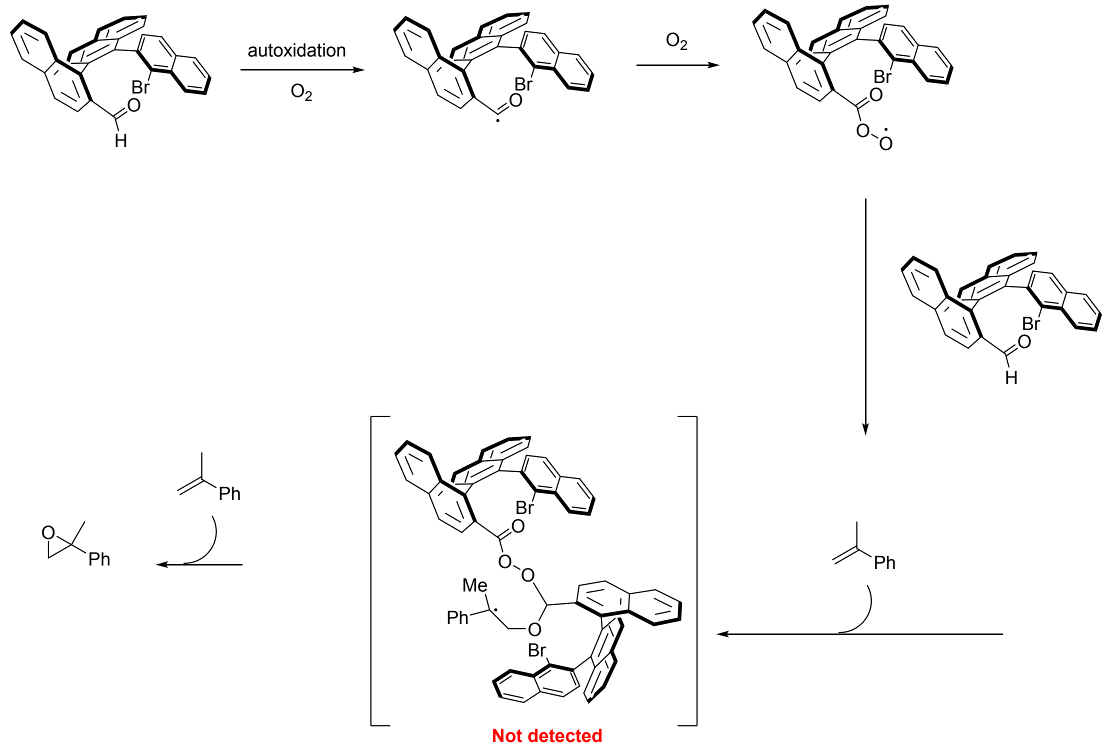

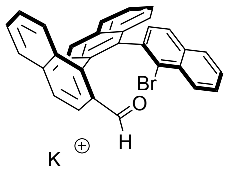

Chemical Formula: $C_{31}H_{19}BrKO^{+}$
Exact Mass: 525,0251

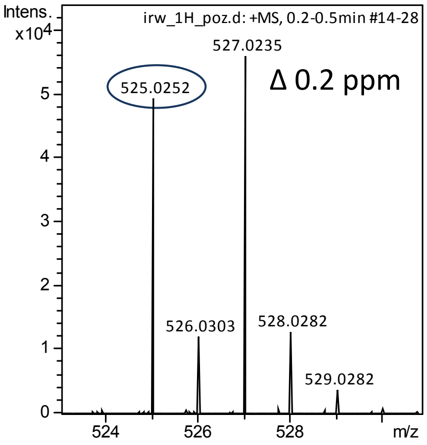

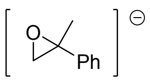

Chemical Formula: $C_9H_9O^{-}$
Exact Mass: 133,0659

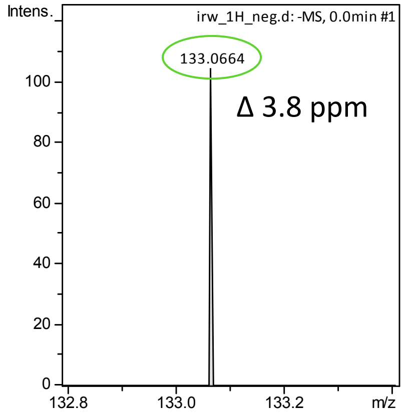

Mechanistic Pathway 2

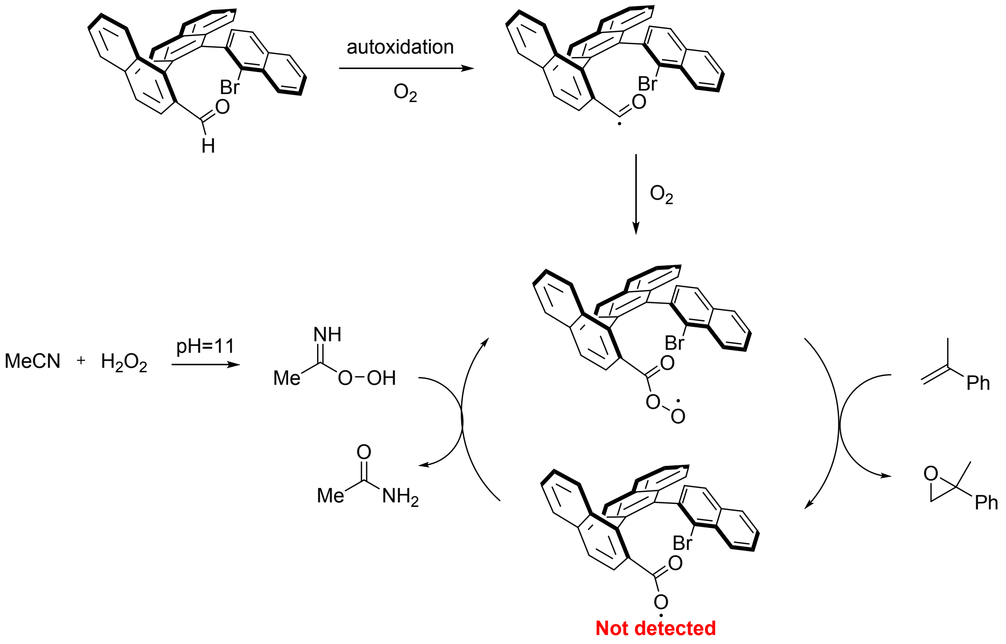

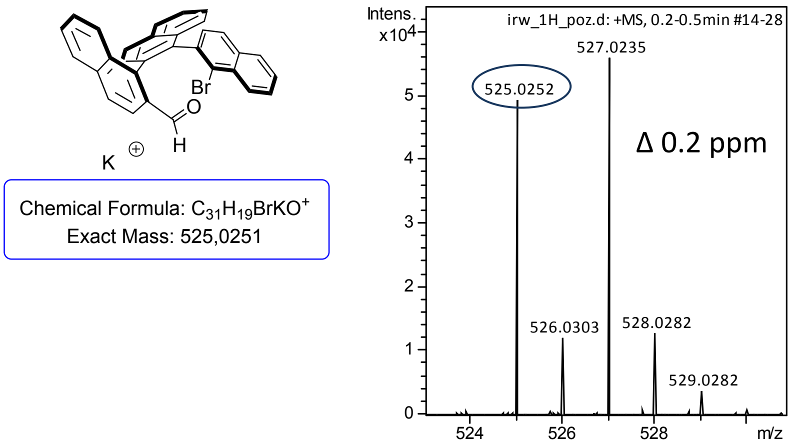

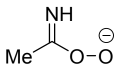

Chemical Formula: $C_2H_4NO_2^-$
Exact Mass: 74,0248

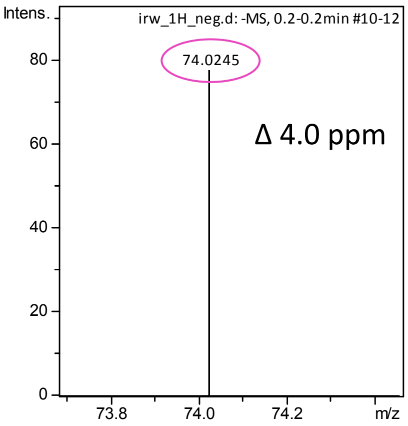

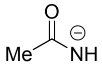

Chemical Formula: $C_2H_4NO^-$
Exact Mass: 58,0298

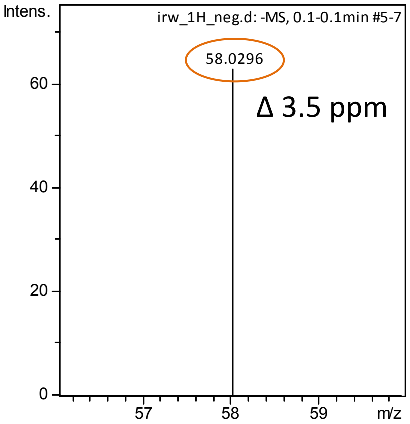

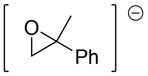

Chemical Formula: $C_9H_9O^-$
Exact Mass: 133,0659

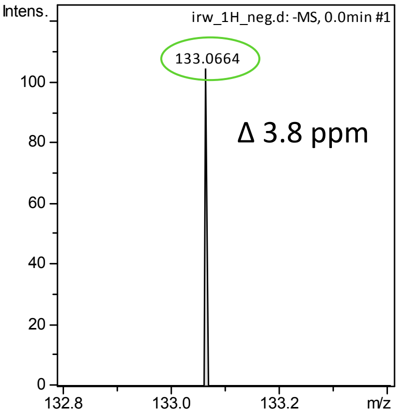

Mechanistic Pathway 3

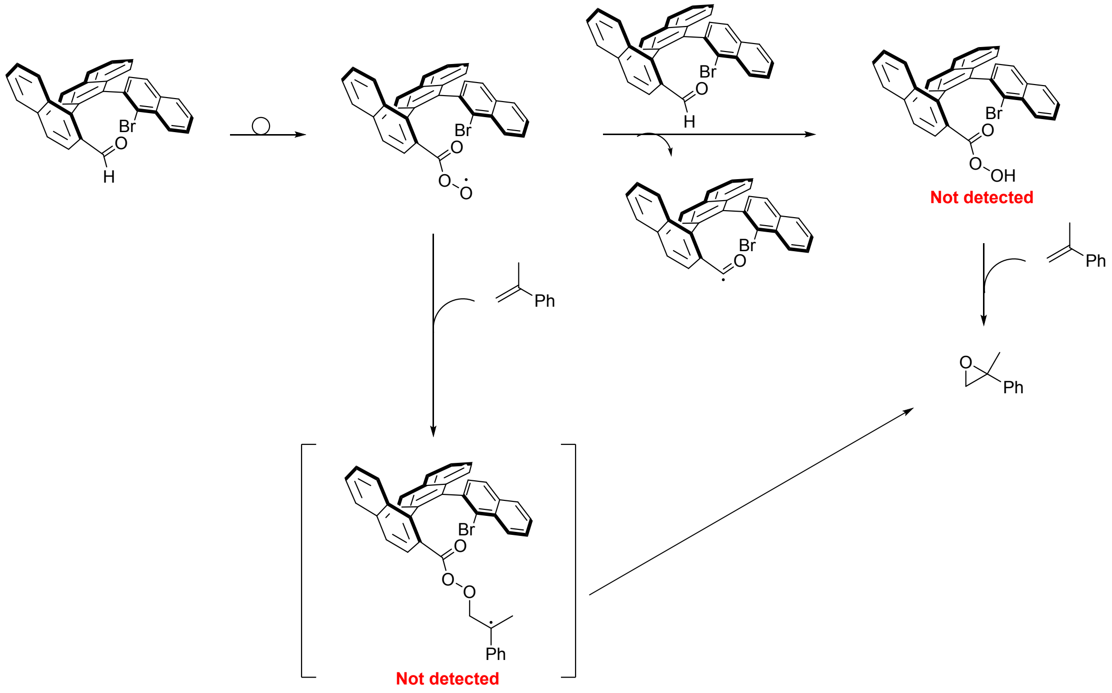

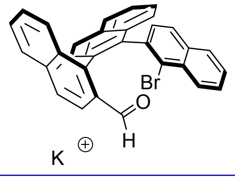

Chemical Formula: $C_{31}H_{19}BrKO^{+}$
Exact Mass: 525,0251

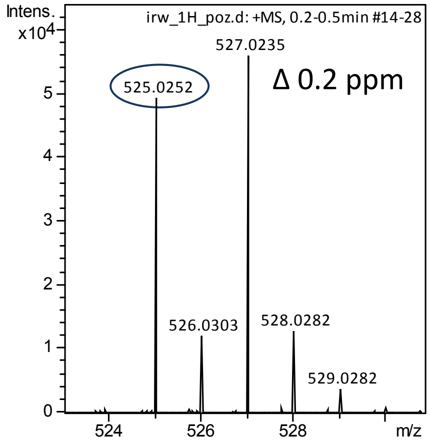

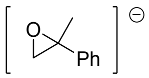

Chemical Formula: $C_9H_9O^-$
Exact Mass: 133,0659

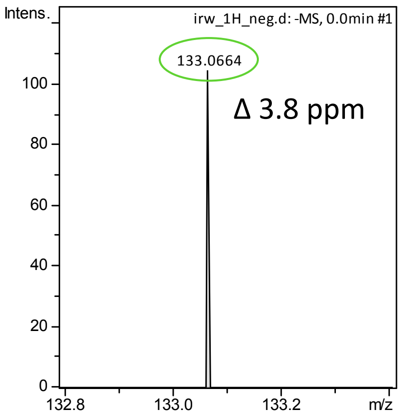

Mechanistic Pathway 4

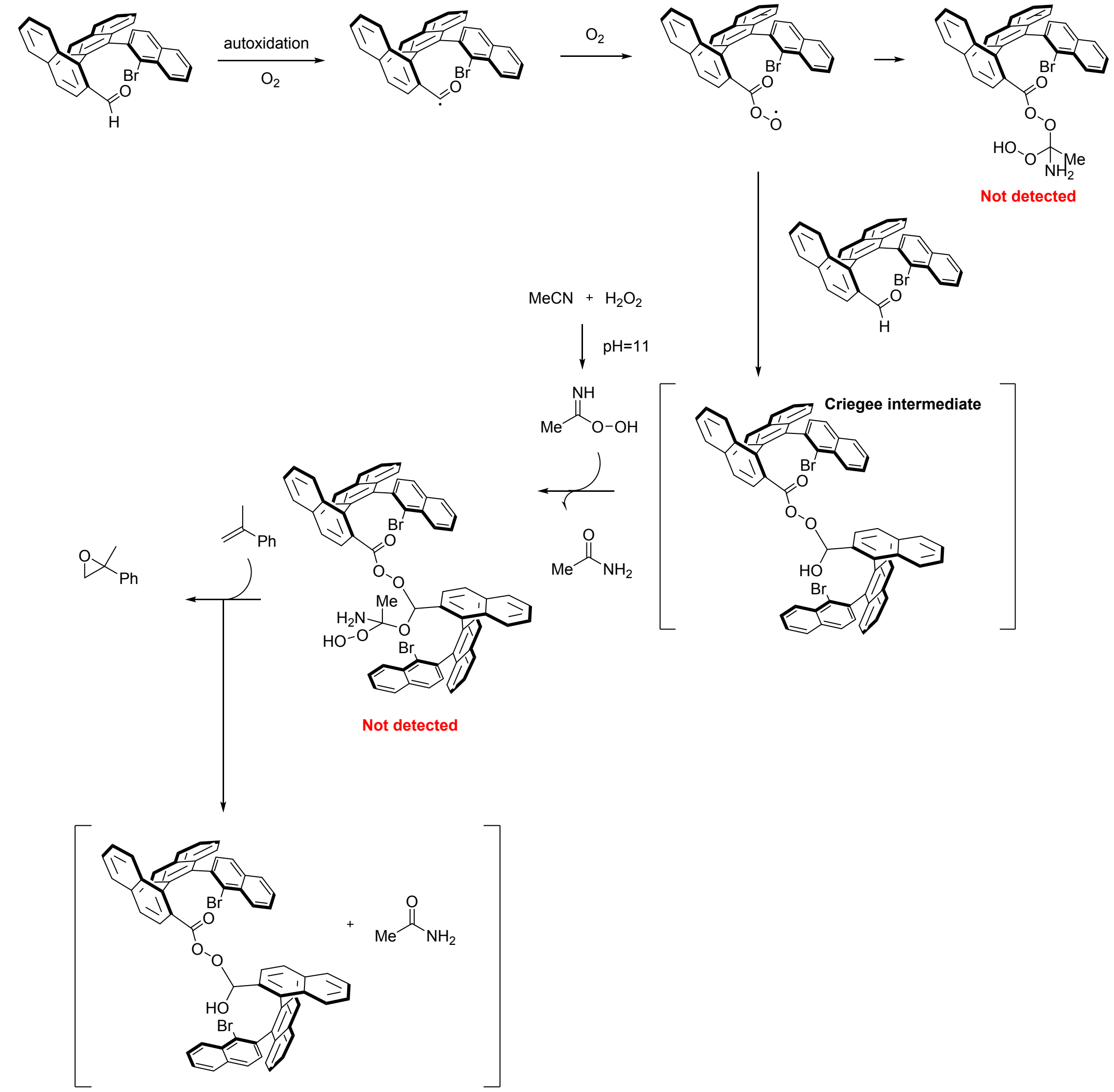

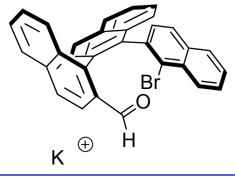

Chemical Formula: $C_{31}H_{19}BrKO^{+}$
Exact Mass: 525,0251

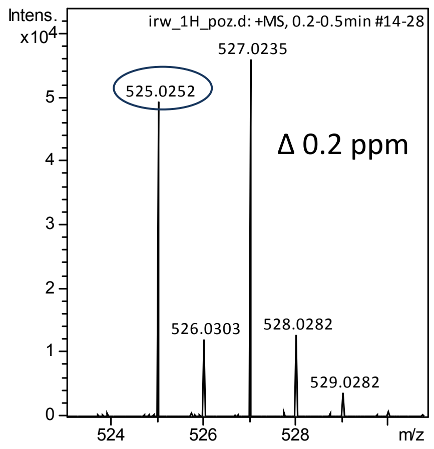

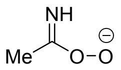

Chemical Formula: $C_2H_4NO_2^-$
Exact Mass: 74,0248

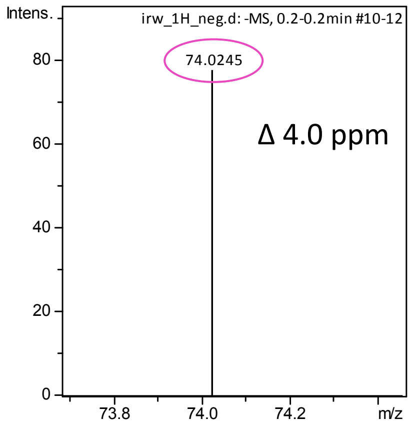

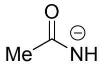

Chemical Formula: $C_2H_4NO^-$
Exact Mass: 58,0298

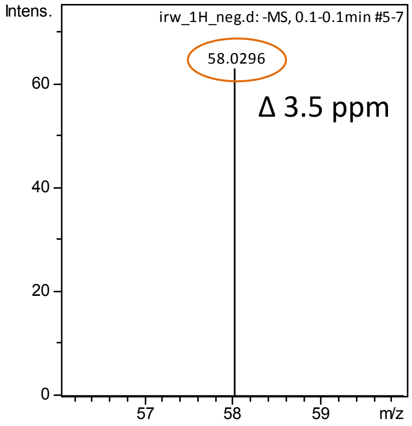

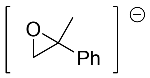

Chemical Formula: $C_9H_9O^-$
Exact Mass: 133,0659

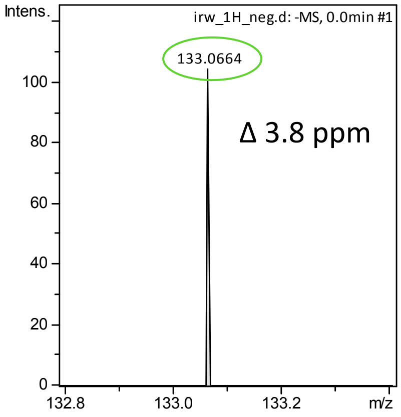

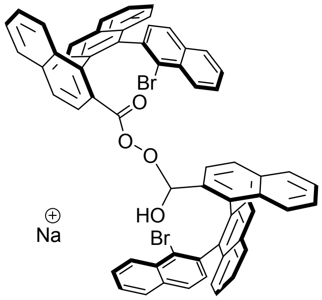

Chemical Formula: $C_{62}H_{38}Br_2NaO_4^+$
Exact Mass: 1027,1029

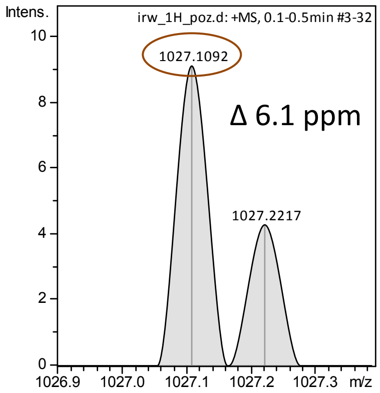

Mechanistic Pathway 5

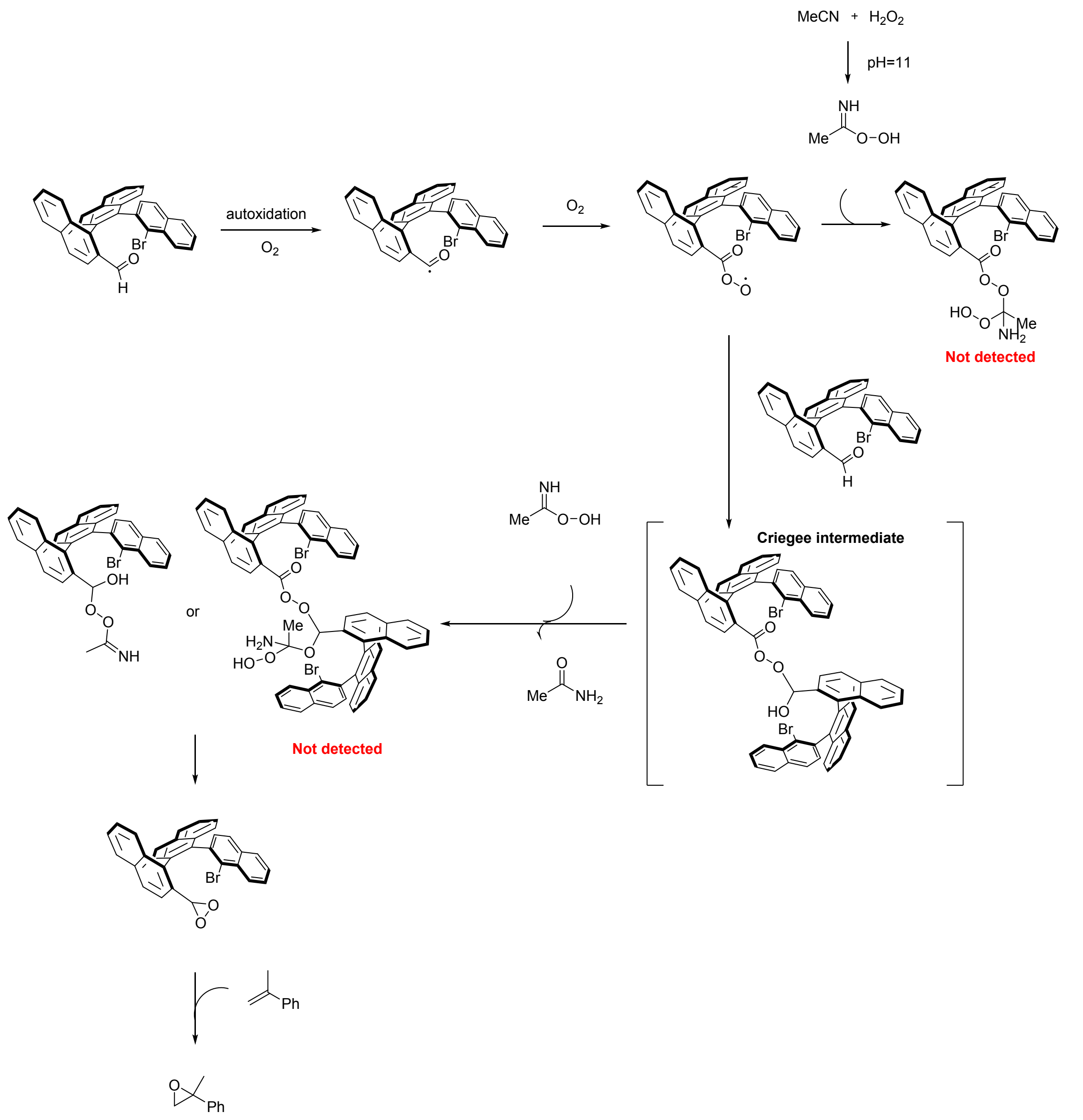

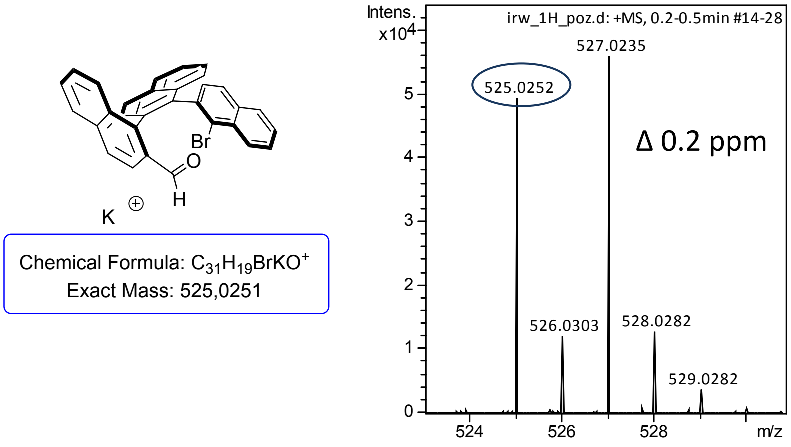

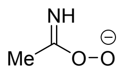

Chemical Formula: $C_2H_4NO_2^-$
Exact Mass: 74,0248

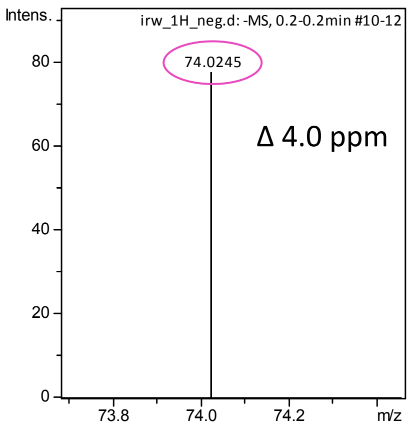

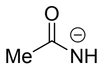

Chemical Formula: $C_2H_4NO^-$
Exact Mass: 58,0298

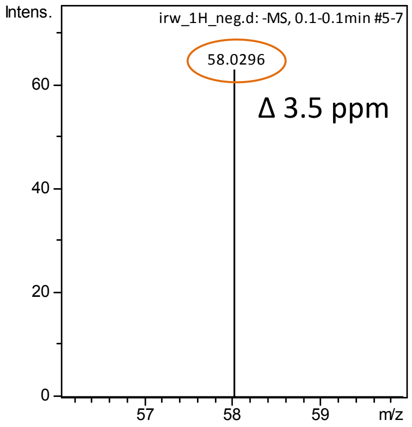

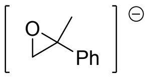

Chemical Formula: $C_9H_9O^-$
Exact Mass: 133,0659

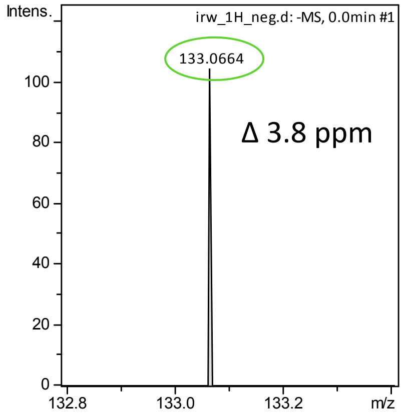

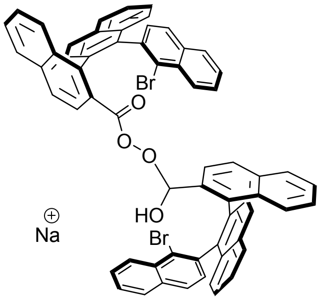

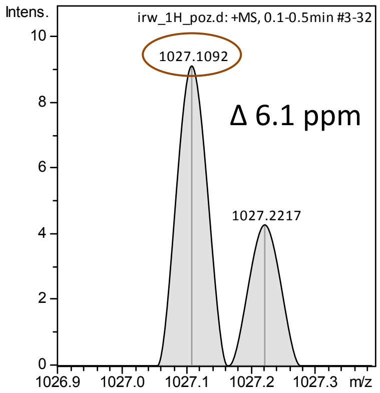

Chemical Formula: $C_{62}H_{38}Br_2NaO_4^+$
Exact Mass: 1027,1029

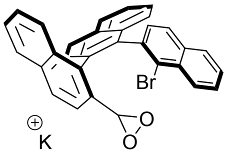

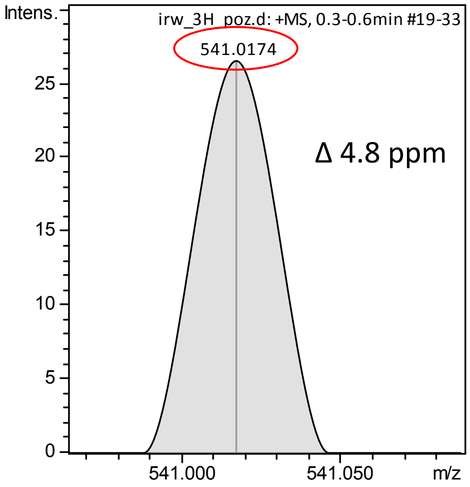

Chemical Formula: $C_{31}H_{19}BrKO_2^+$
Exact Mass: 541,0200

In order to rule out any potential artifacts and be sure about the proposed mechanistic pathway, we repeated the procedure utilizing 2-bromobenzaldehyde (**3k**) as 'catalyst-witness' and 1-phenyl-cyclohexene (**1o**) as the substrate.

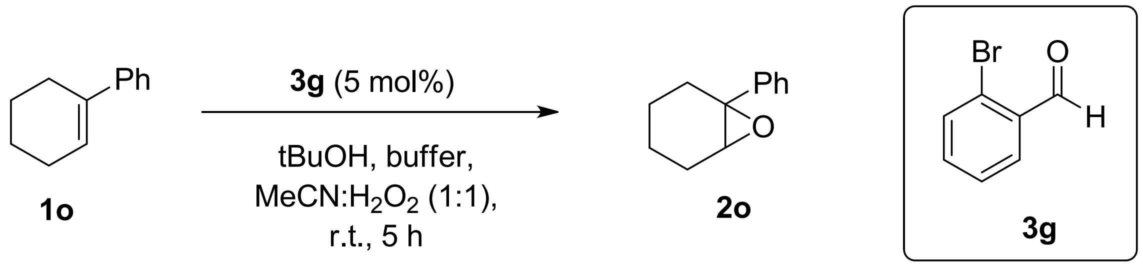

1-Phenyl-cyclohexene (32 mg, 0.20 mmol) was placed in a round bottom flask followed by catalyst **3k** (3 mg, 0.01 mmol). *tert*-Butanol (0.2 mL), aqueous buffer solution (0.2 mL, 0.6M K_2CO_3 – 4 x 10^{-4} M EDTA disodium salt), acetonitrile (0.02 mL, 0.40 mmol) and 30% aqueous H_2O_2 (0.04 mL, 0.40 mmol) were added consecutively. Following the reaction by HRMS and focusing on the possible mechanistic pathway, initially, full scan spectra in both negative and positive mode are presented below:

Negative ESI Mode

0 min

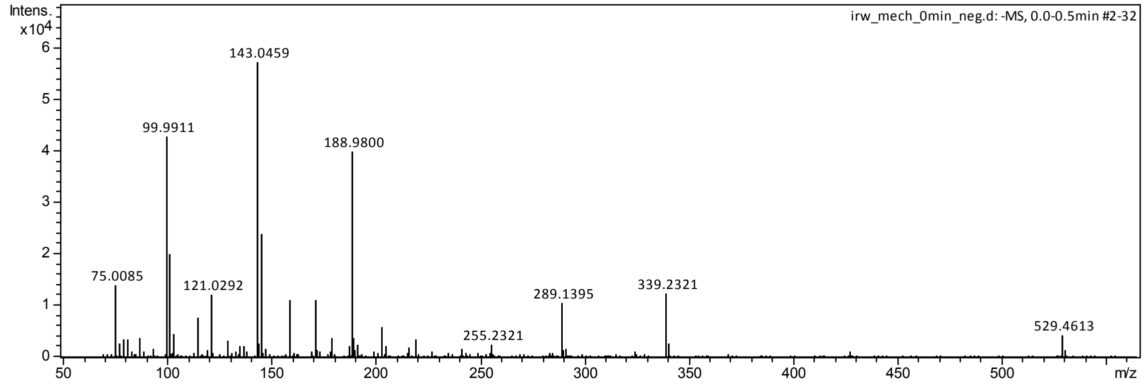

20 min

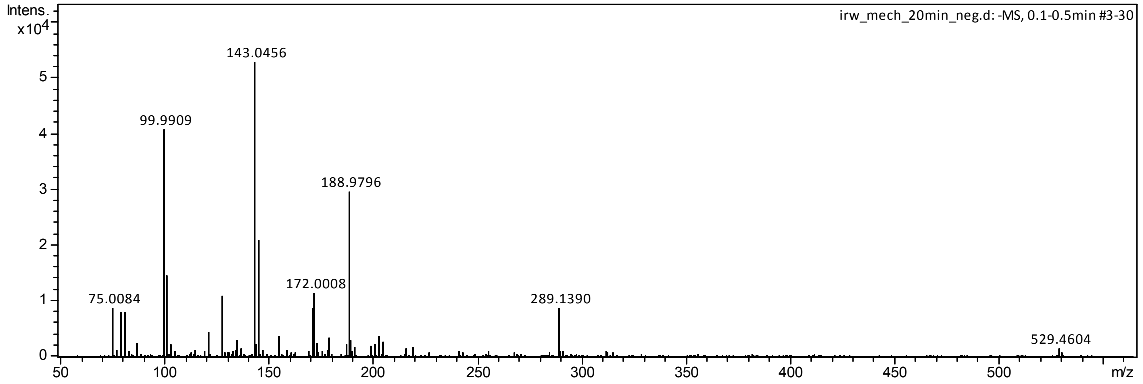

40 min

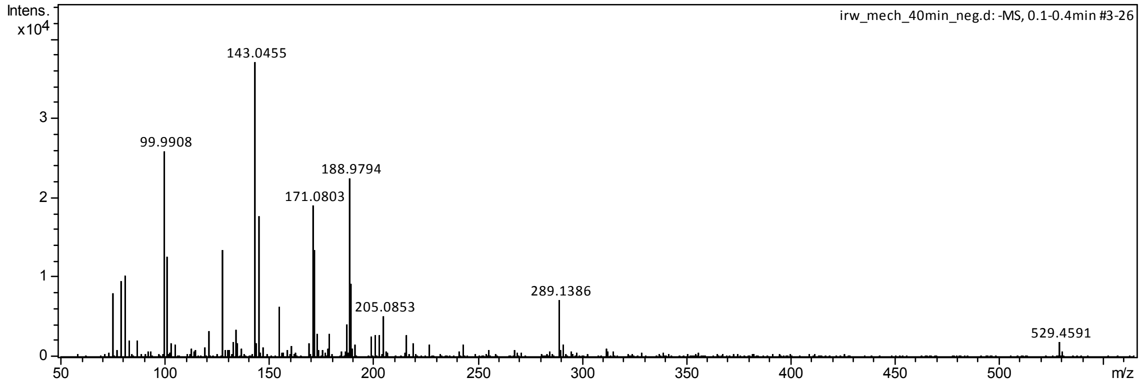

1 h

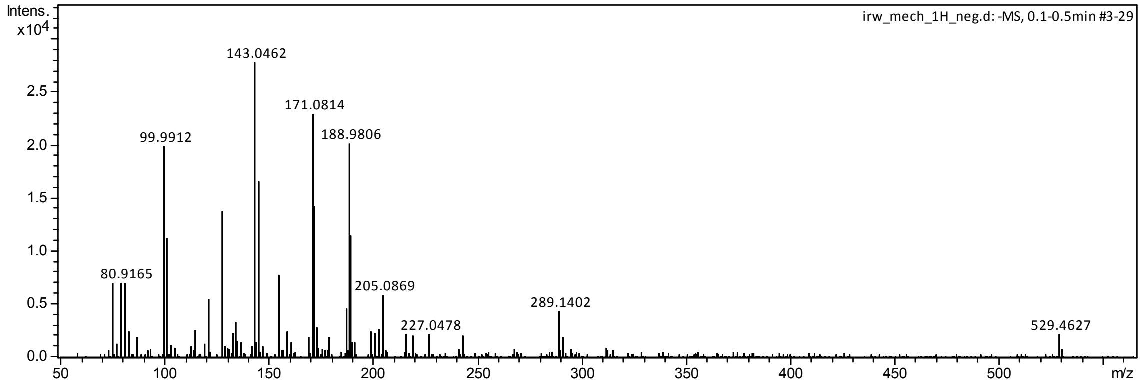

2 h

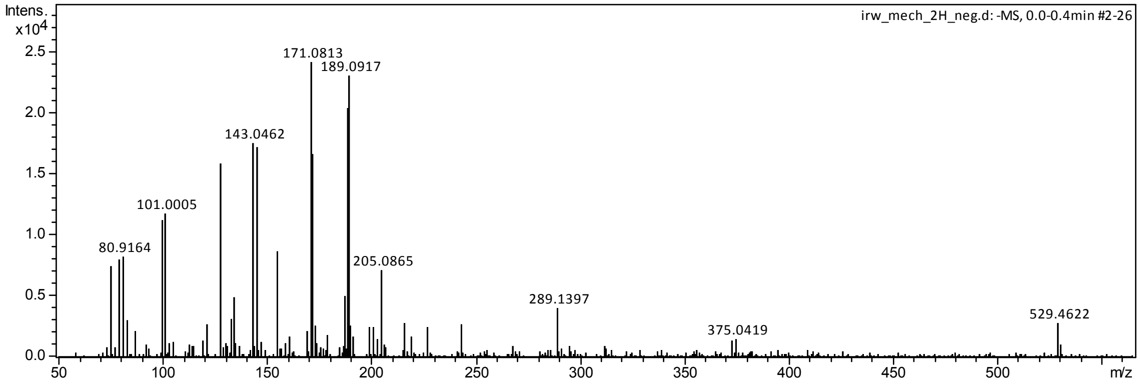
Intens.
x10^4
irw_mech_2H_neg.d: -MS, 0.0-0.4min #2-26
171.0813
189.0917
143.0462
101.0005
80.9164
205.0865
289.1397
375.0419
529.4622
m/z

3 h

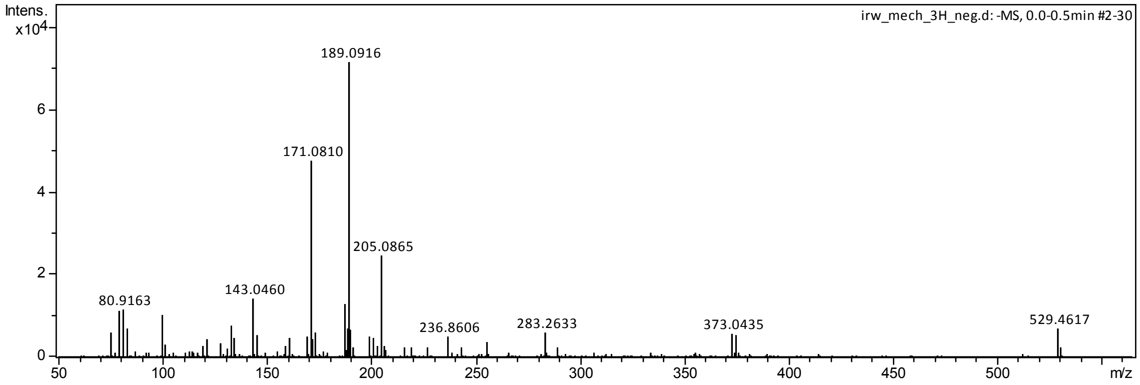
Intens.
x10^4
irw_mech_3H_neg.d: -MS, 0.0-0.5min #2-30
189.0916
171.0810
205.0865
80.9163
143.0460
236.8606
283.2633
373.0435
529.4617
m/z

Positive ESI Mode

0 min

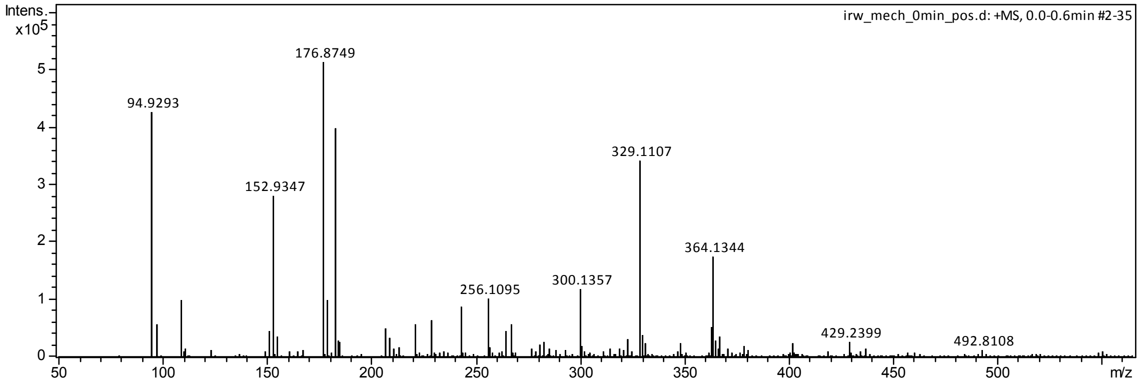

20 min

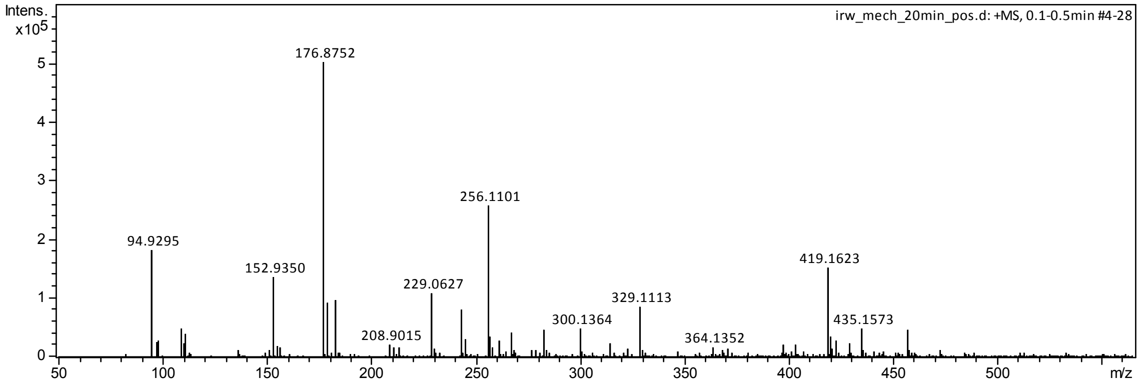

40 min

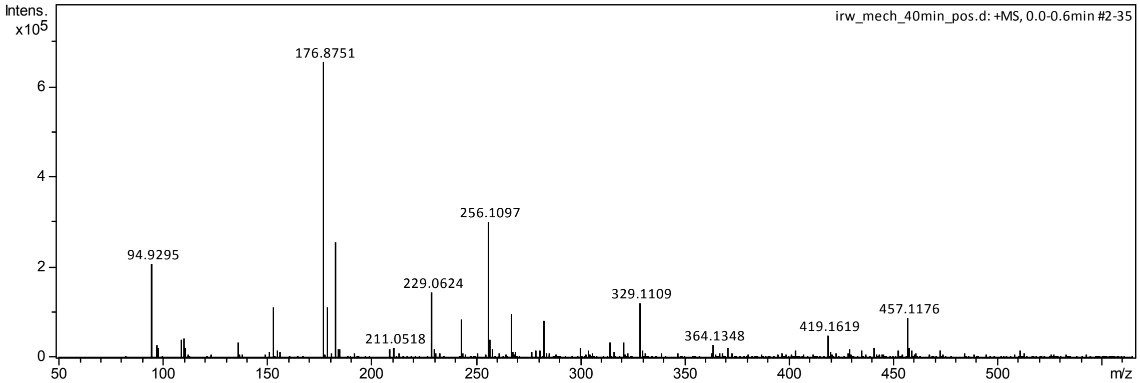

1 h

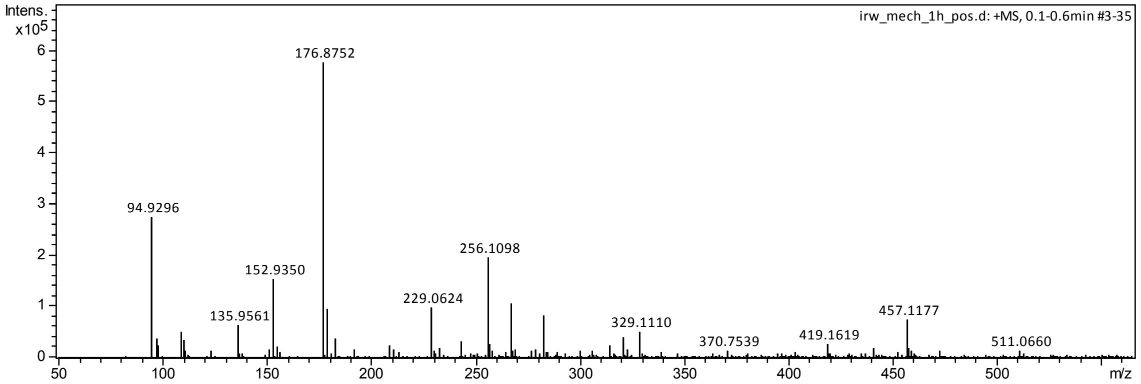

2 h

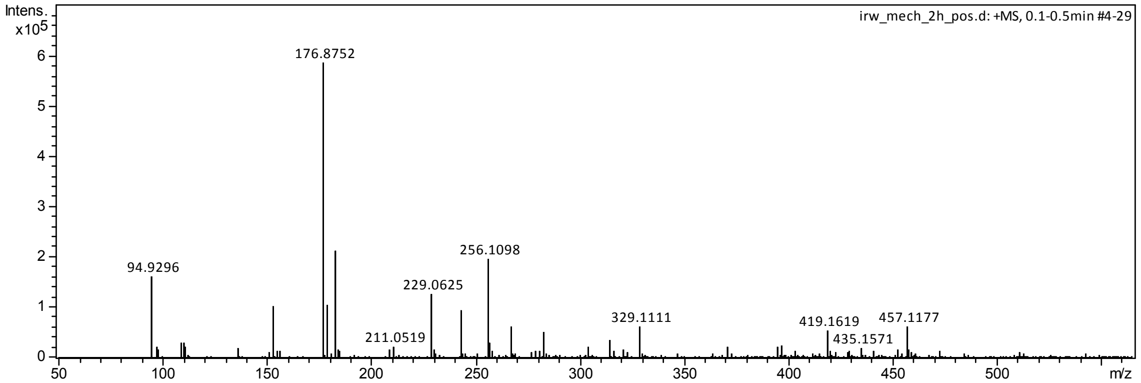

3 h

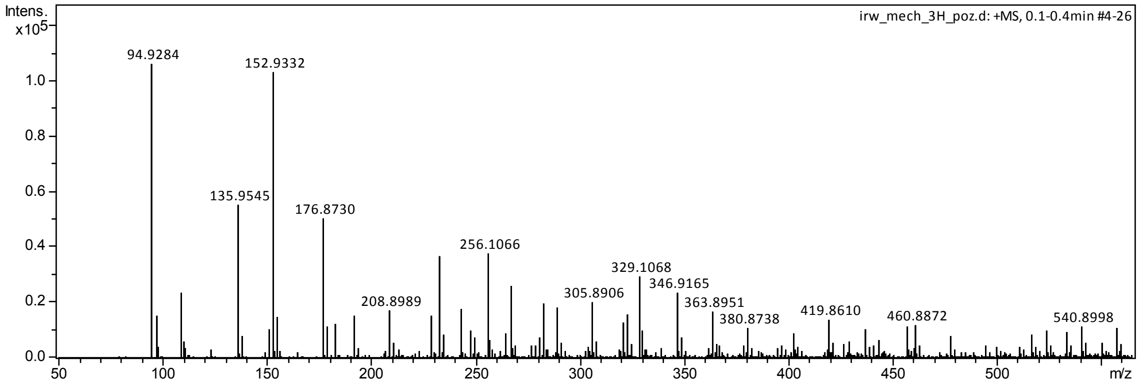

In the following part, the proposed mechanistic pathways of the reaction are presented. Intermediates that were detected are presented below.

Mechanistic Pathway 1

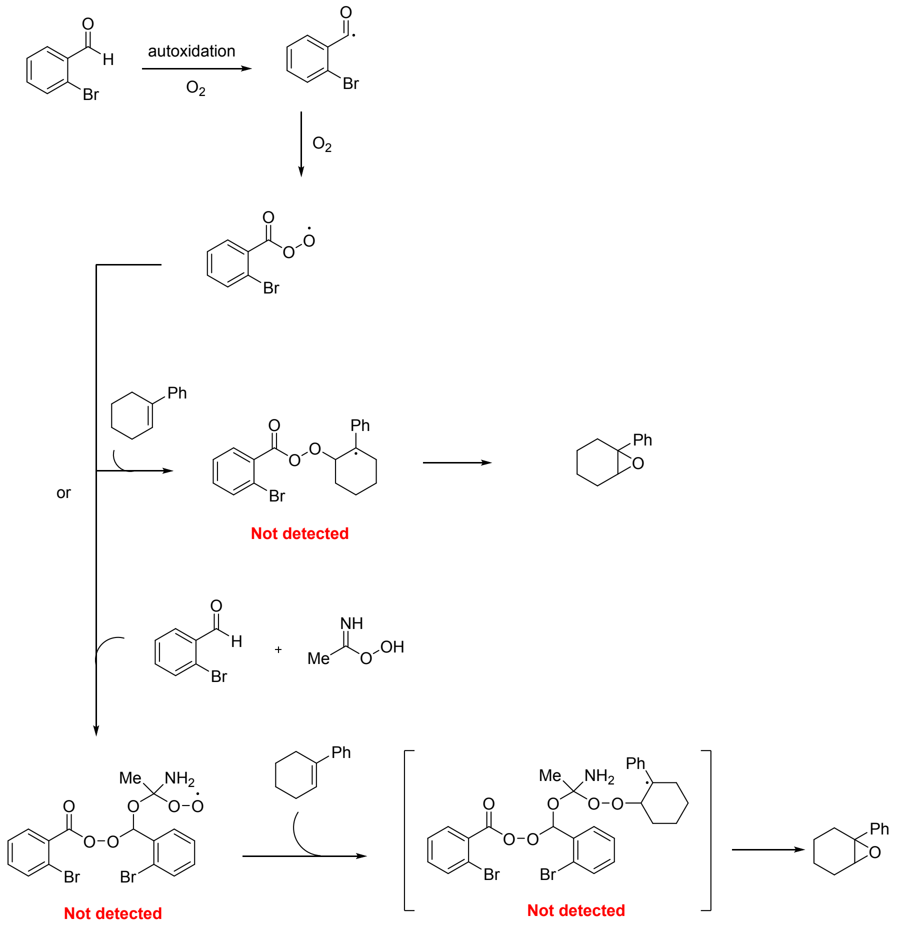

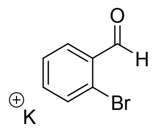

Chemical Formula: $C_7H_5BrKO^+$
Exact Mass: 222,9155

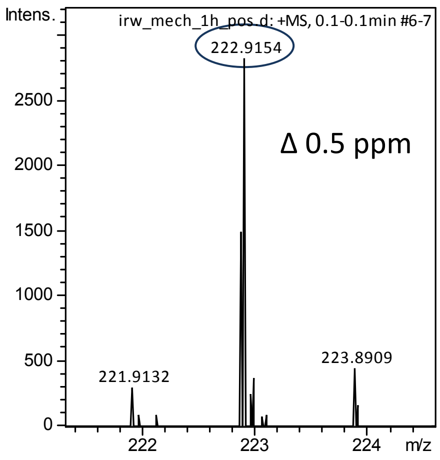

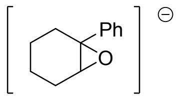

Chemical Formula: $C_{12}H_{13}O^-$
Exact Mass: 173,0972

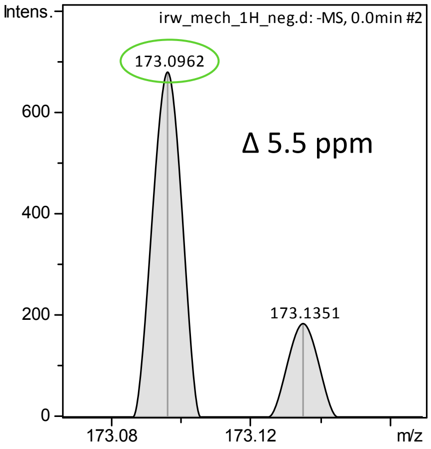

Mechanistic Pathway 2

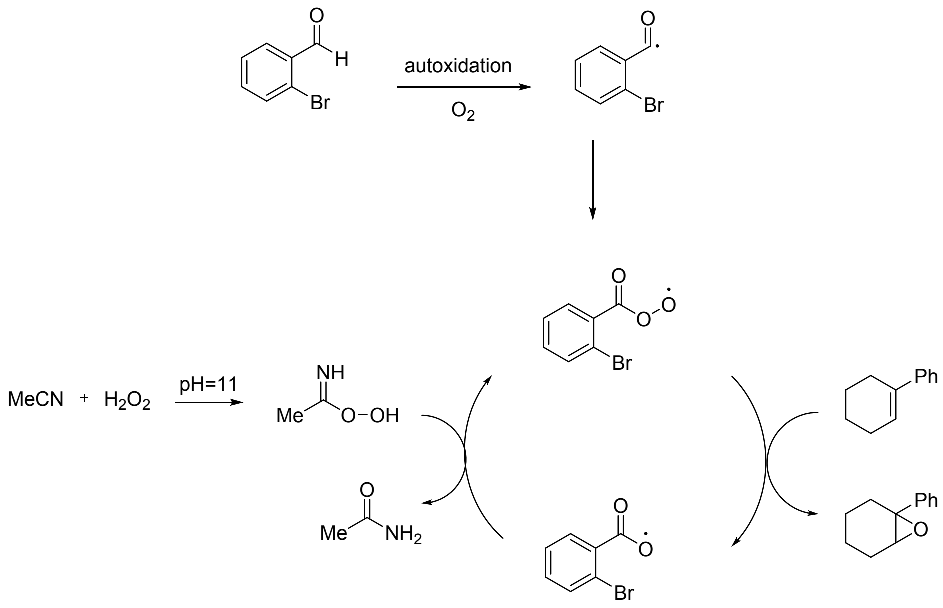

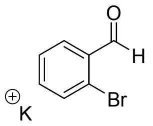

Chemical Formula: $C_7H_5BrKO^+$
Exact Mass: 222,9155

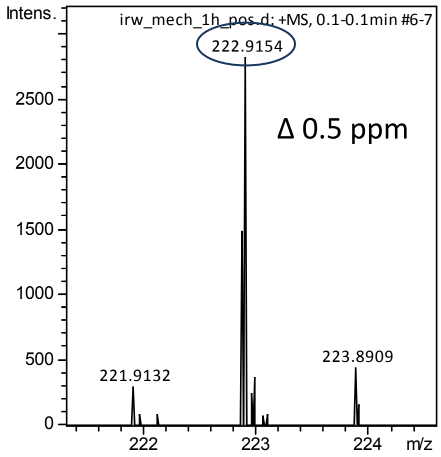

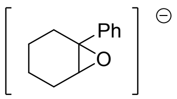

Chemical Formula: $C_{12}H_{13}O^-$
Exact Mass: 173,0972

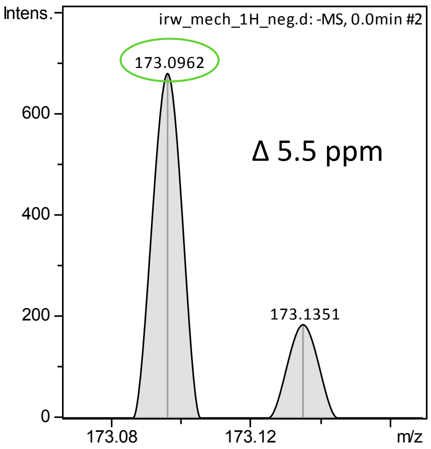

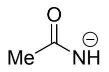

Chemical Formula: $C_2H_4NO^-$
Exact Mass: 58,0298

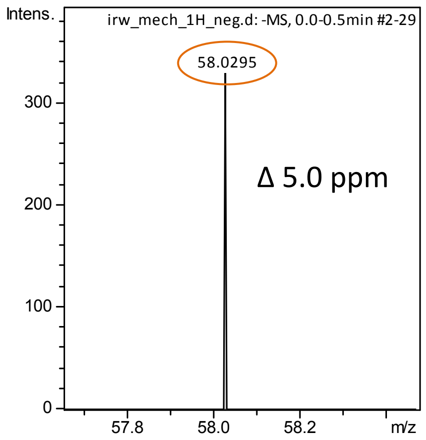

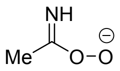

Chemical Formula: $C_2H_4NO_2^-$
Exact Mass: 74,0248

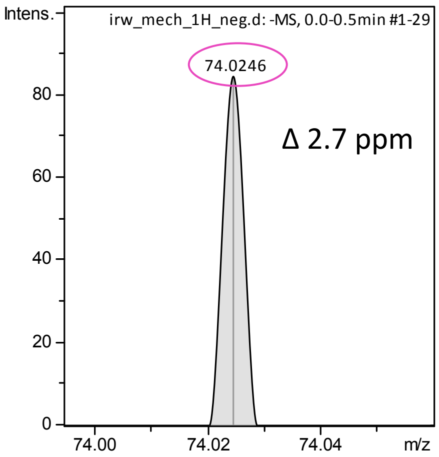

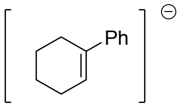

Chemical Formula: $C_{12}H_{13}^-$
Exact Mass: 157,1023

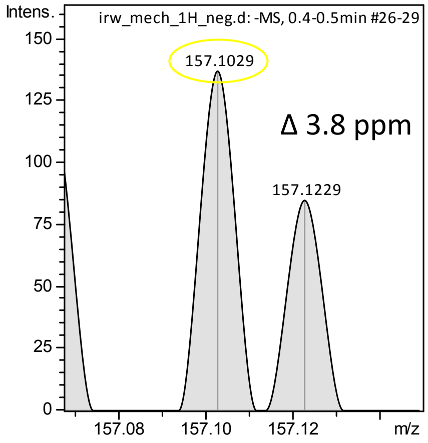

Mechanistic Pathway 3

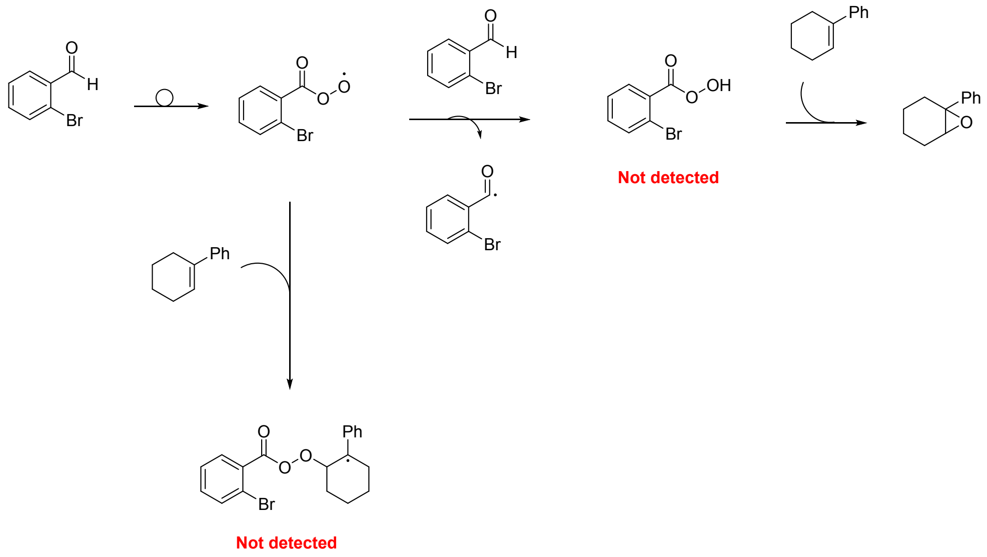

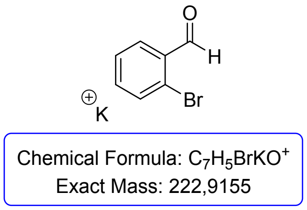

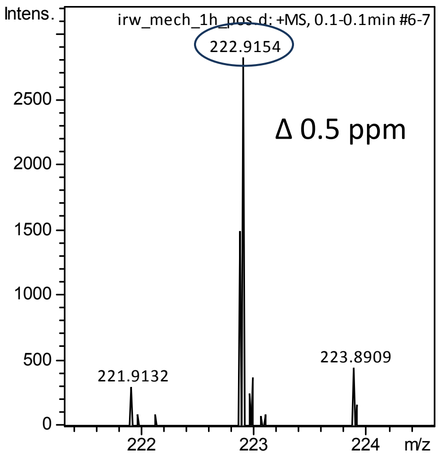

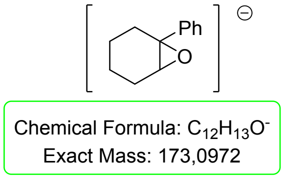

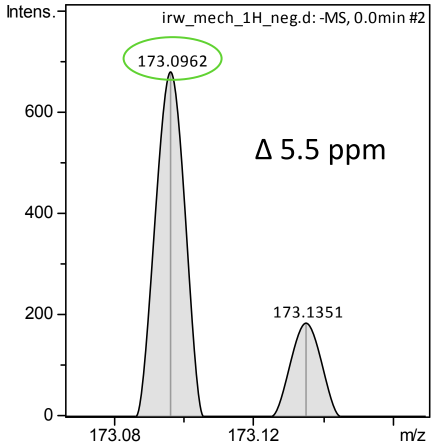

Mechanistic Pathway 4

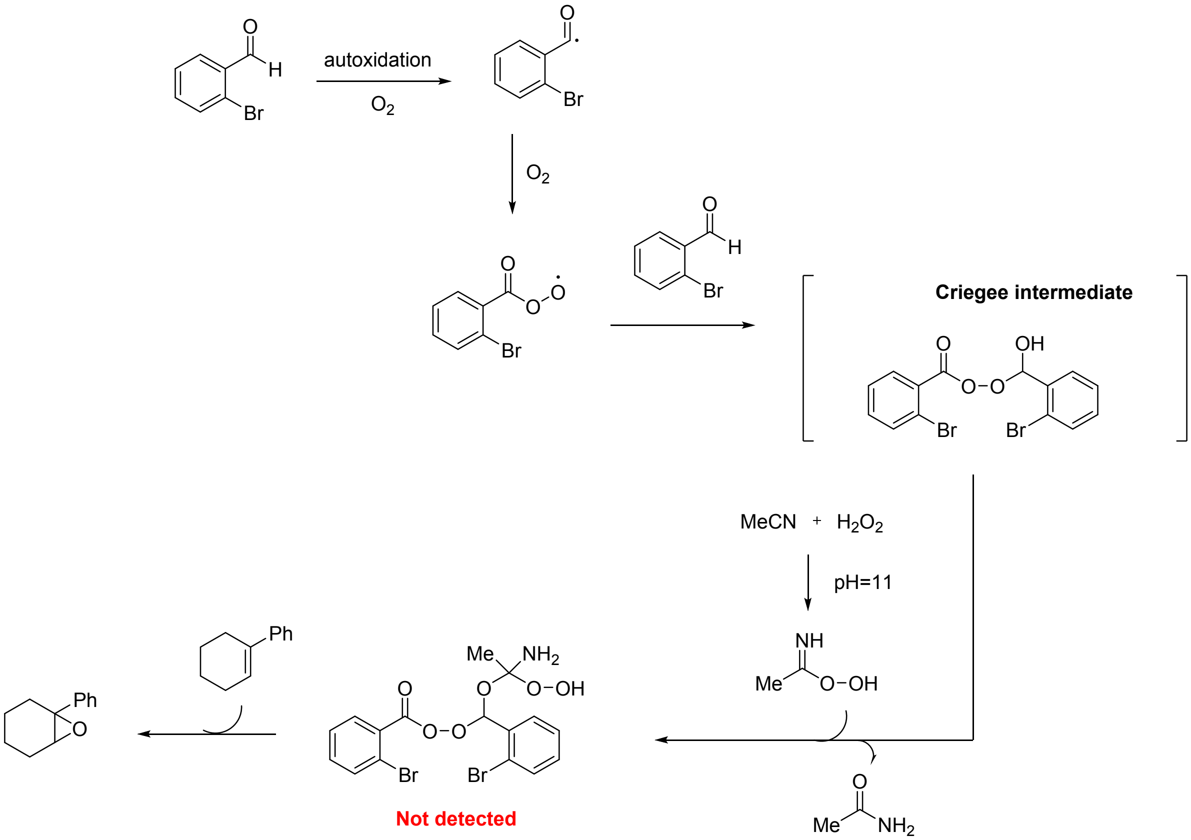

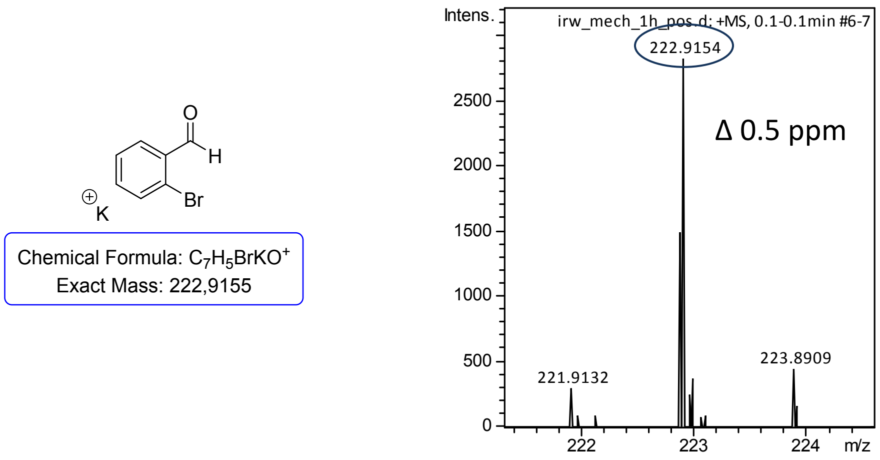

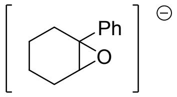

Chemical Formula: $C_{12}H_{13}O^-$
Exact Mass: 173,0972

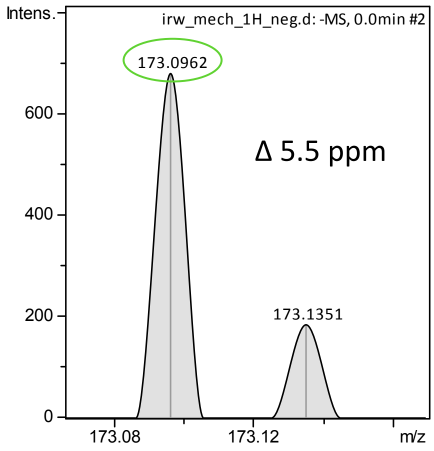

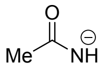

Chemical Formula: $C_2H_4NO^-$
Exact Mass: 58,0298

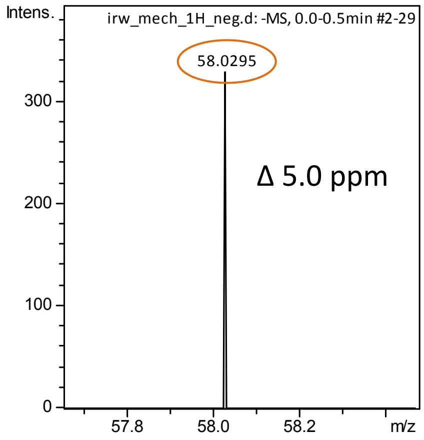

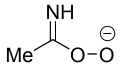

Chemical Formula: $C_2H_4NO_2^-$
Exact Mass: 74,0248

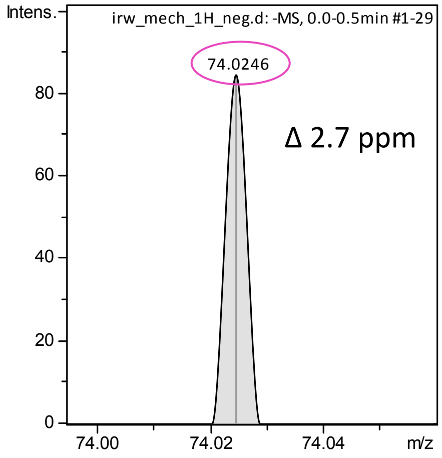

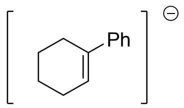

Chemical Formula: $C_{12}H_{13}^-$
Exact Mass: 157,1023

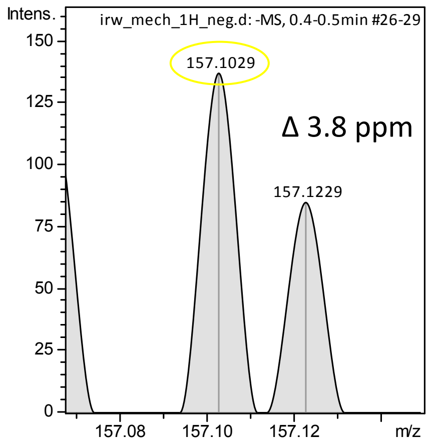

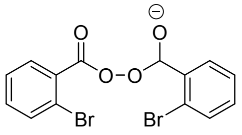

Chemical Formula: $C_{14}H_9Br_2O_4^-$
Exact Mass: 398,8873

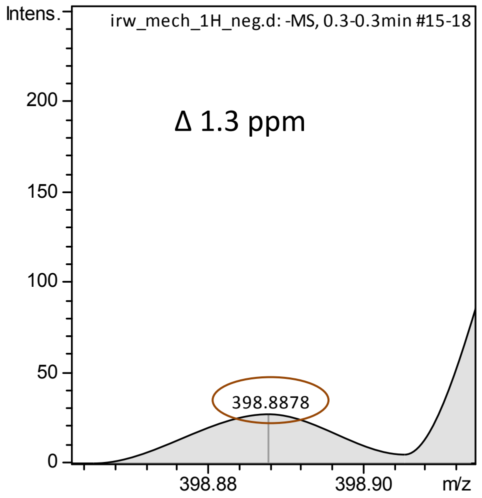

Mechanistic Pathway 5

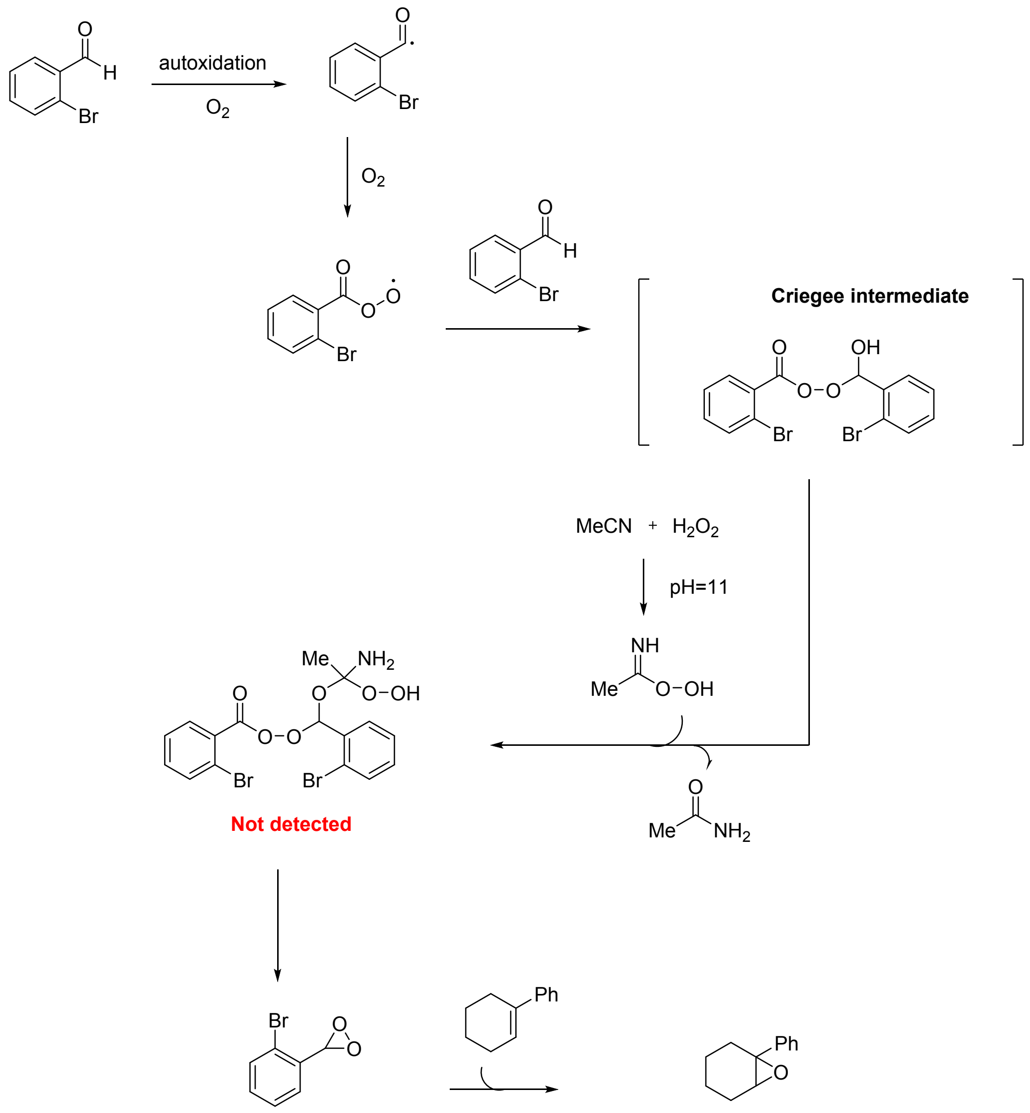

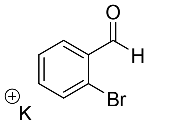

Chemical Formula: $C_7H_5BrKO^+$
Exact Mass: 222,9155

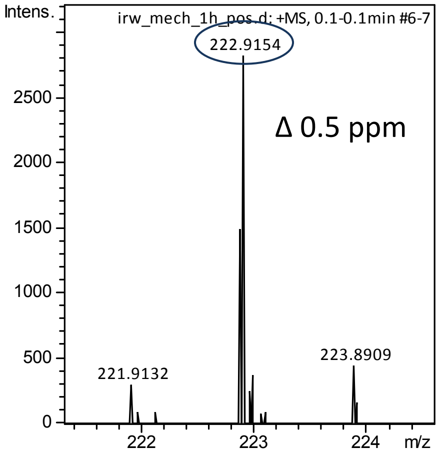

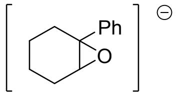

Chemical Formula: $C_{12}H_{13}O^{-}$
Exact Mass: 173,0972

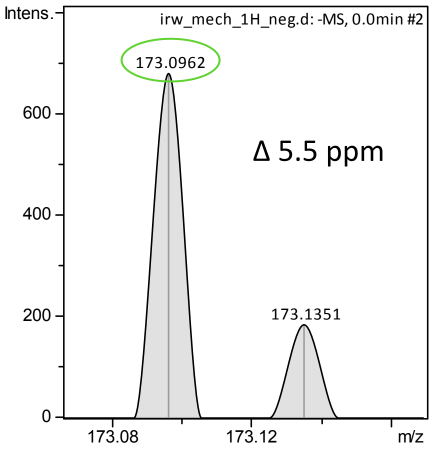

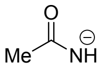

Chemical Formula: $C_2H_4NO^{-}$
Exact Mass: 58,0298

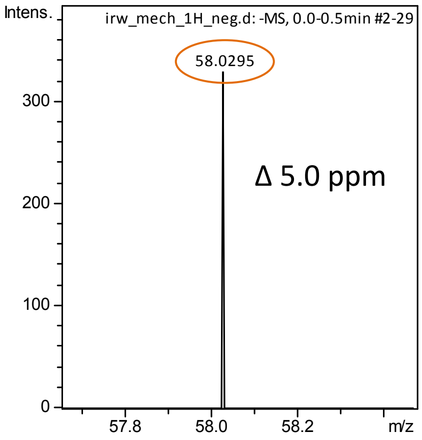

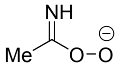

Chemical Formula: $C_2H_4NO_2^{-}$
Exact Mass: 74,0248

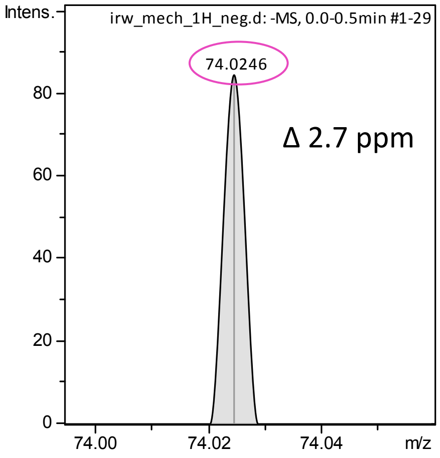

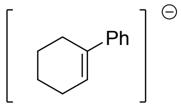

Chemical Formula: $C_{12}H_{13}^{-}$
Exact Mass: 157,1023

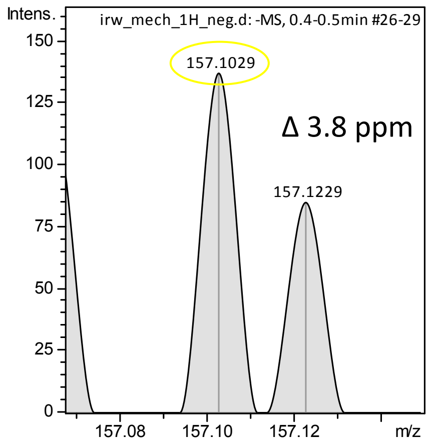

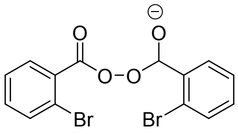

Chemical Formula: $C_{14}H_{9}Br_{2}O_{4}^{-}$
Exact Mass: 398,8873

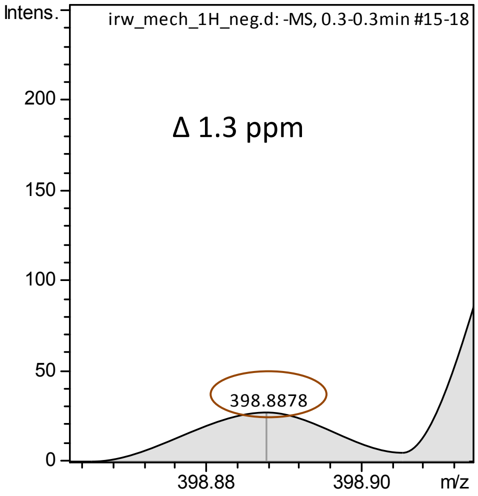

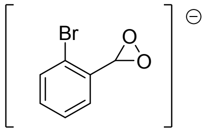

Chemical Formula: $C_{7}H_{4}BrO_{2}^{-}$
Exact Mass: 198,9400

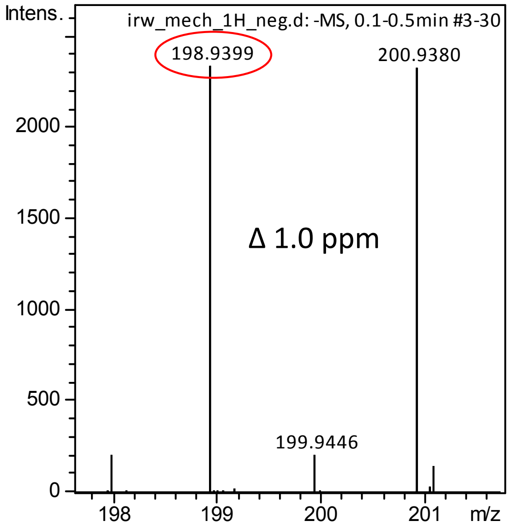

In order to detect the desired intermediates with acetonitrile (CH_3CN), it was replaced by deuterated acetonitrile (CD_3CN). The presence of deuterated Payne's intermediate was detected.

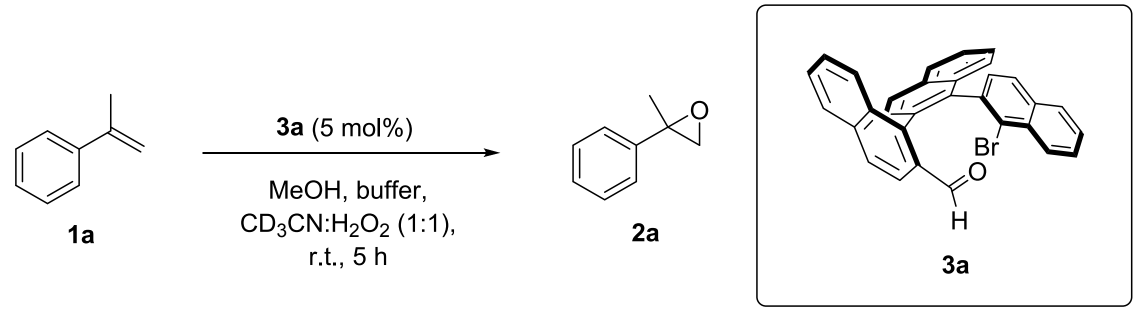

a-Methyl-styrene (24.0 mg, 0.20 mmol) was placed in a round bottom flask followed by catalyst **3a** (4.9 mg, 0.01 mmol). Methanol (0.2 mL), aqueous buffer solution (0.2 mL, 0.6M K_2CO_3 – 4 x 10^{-4} M EDTA disodium salt), deuterated acetonitrile (0.02 mL, 0.40 mmol) and 30% aqueous H_2O_2 (0.04 mL, 0.40 mmol) were added consecutively.

Negative ESI Mode

0 min

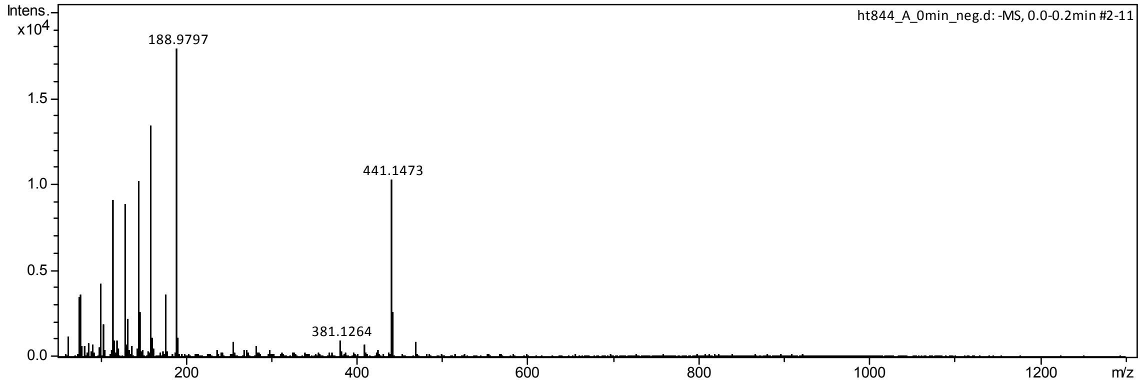

30 min

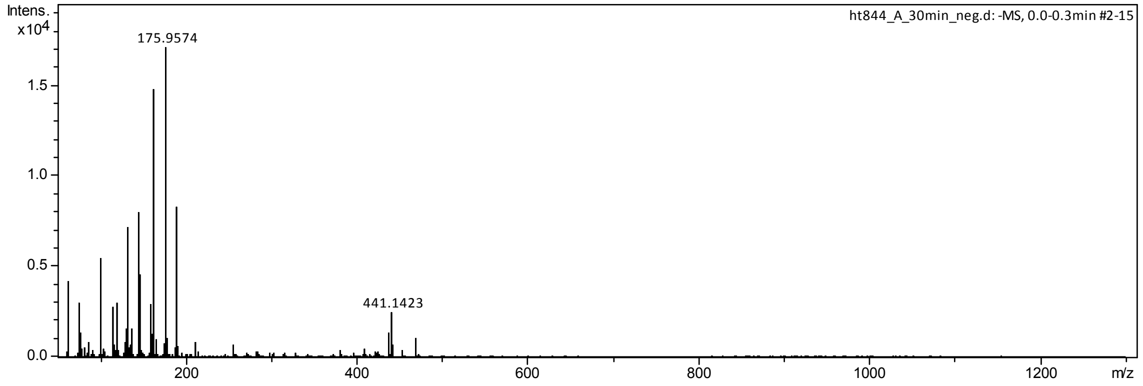

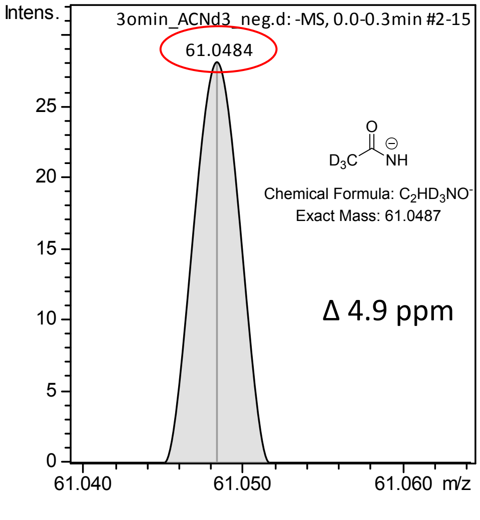

Positive ESI Mode

4 h

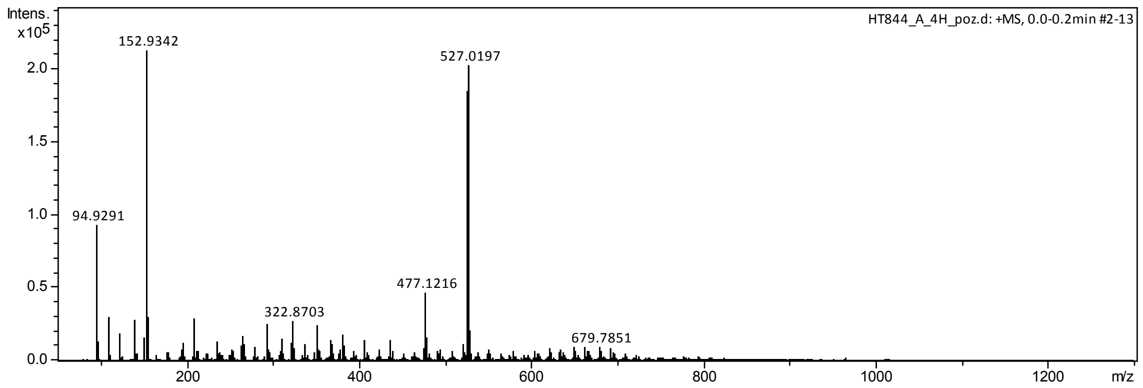

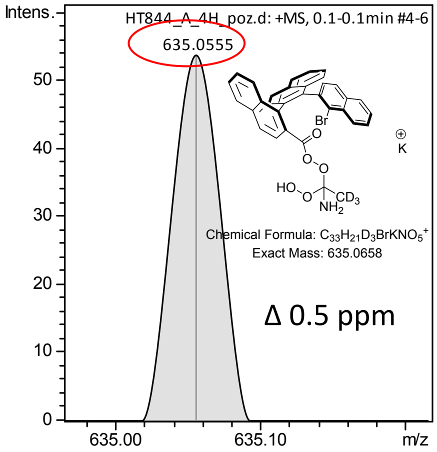

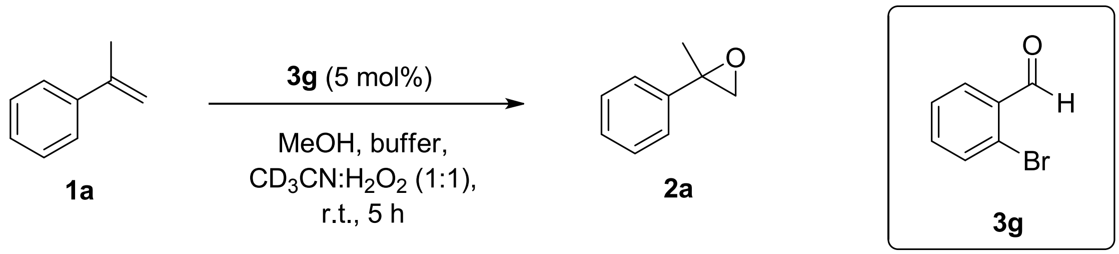

a-Methyl-styrene (24.0 mg, 0.20 mmol) was placed in a round bottom flask followed by catalyst **3k** (2.7 mg, 0.01 mmol). Methanol (0.2 mL), aqueous buffer solution (0.2 mL, 0.6M K_2CO_3 – 4 x 10^{-4} M EDTA disodium salt), deuterated acetonitrile (0.02 mL, 0.40 mmol) and 30% aqueous H_2O_2 (0.04 mL, 0.40 mmol) were added consecutively.

Negative ESI Mode

1 h

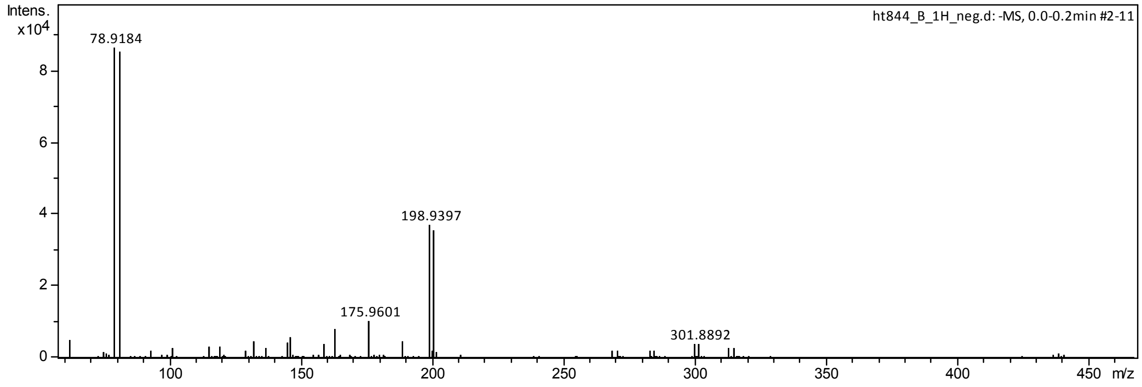

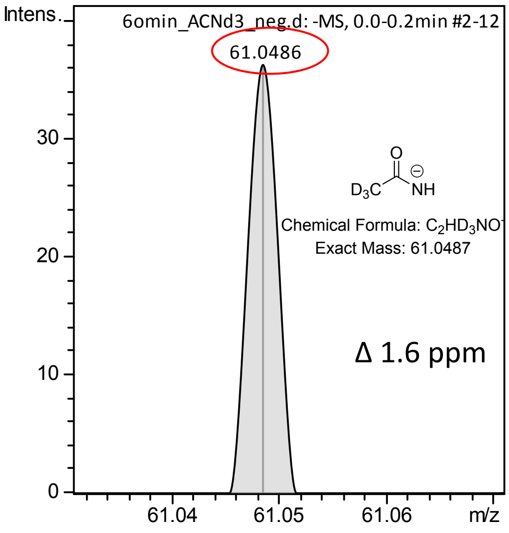

Positive ESI Mode

0 min

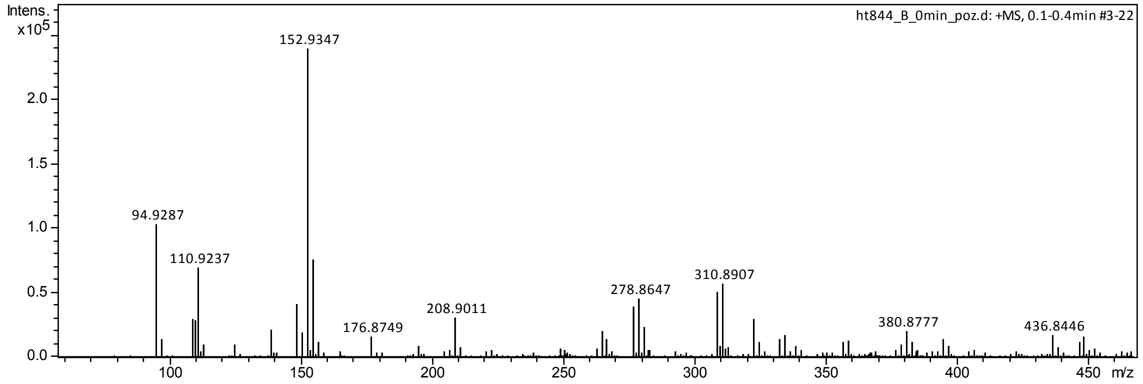

30 min

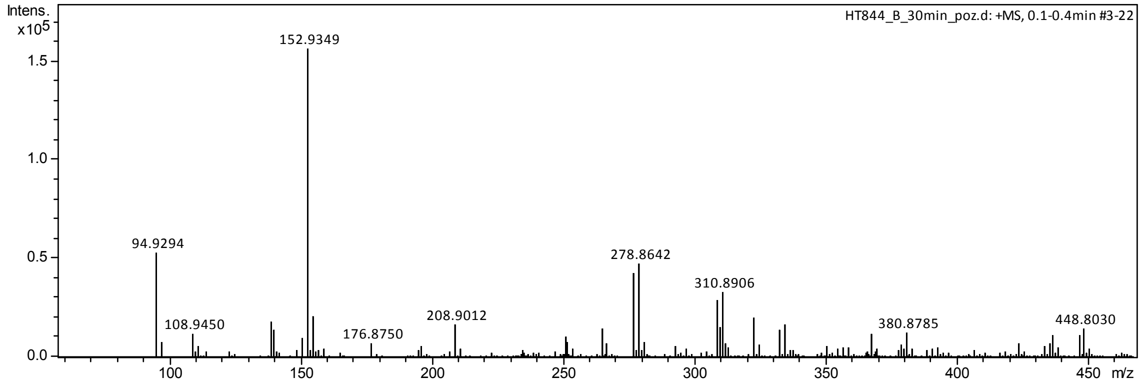

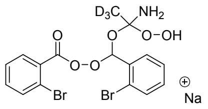

Chemical Formula: $C_{16}H_{12}D_3Br_2NNaO_6^+$
Exact Mass: 500,9347

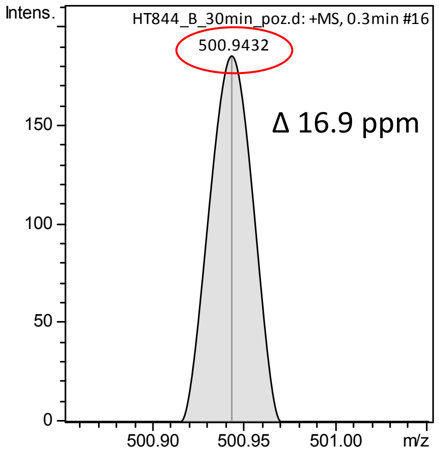

1 h

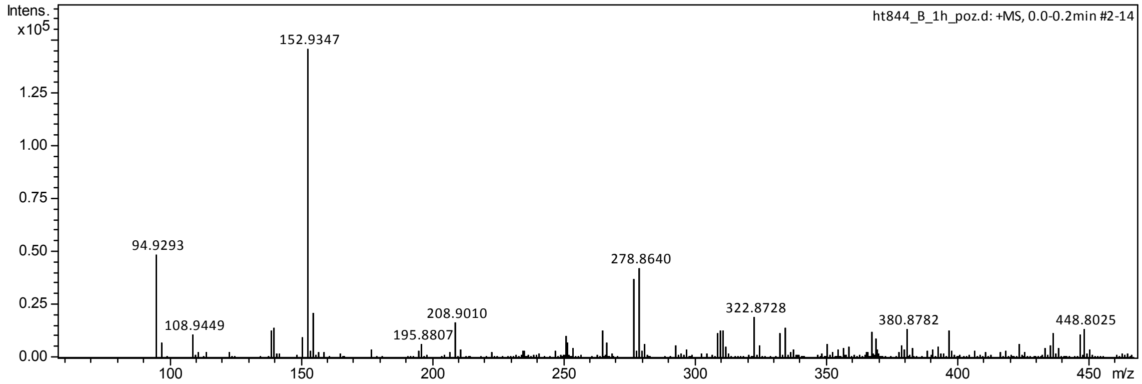
ht844_B_1h_poz.d: +MS, 0.0-0.2min #2-14
Intens. x10^5
152.9347
94.9293
108.9449
195.8807
208.9010
278.8640
322.8728
380.8782
448.8025
m/z

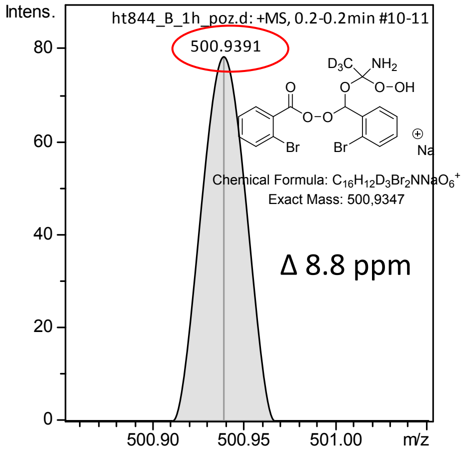
ht844_B_1h_poz.d: +MS, 0.2-0.2min #10-11
Intens.
500.9391
Chemical Formula: C16H12D3Br2NNaO6+
Exact Mass: 500,9347
Δ 8.8 ppm
m/z

3 h

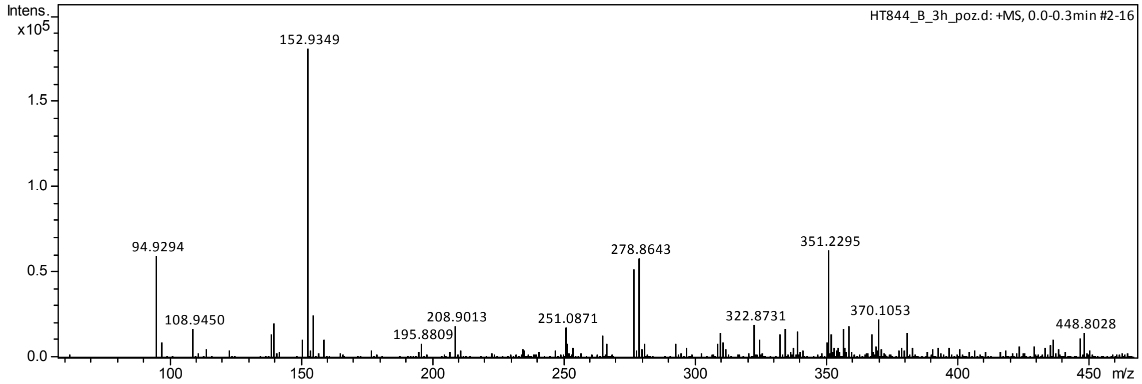
HT844_B_3h_poz.d: +MS, 0.0-0.3min #2-16
Intens. x10^5
152.9349
94.9294
108.9450
195.8809
208.9013
251.0871
278.8643
322.8731
351.2295
370.1053
448.8028
m/z

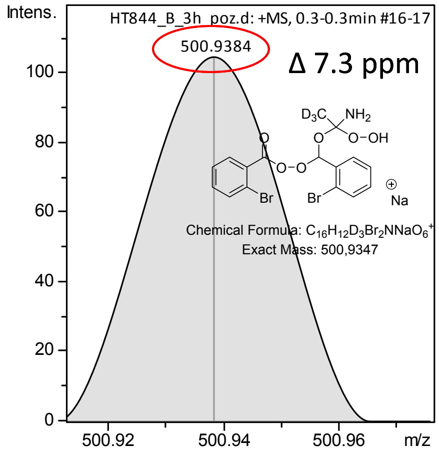
HT844_B_3h_poz.d: +MS, 0.3-0.3min #16-17
Intens.
500.9384
Δ 7.3 ppm
Chemical Formula: C16H12D3Br2NNaO6+
Exact Mass: 500,9347
m/z

4 h

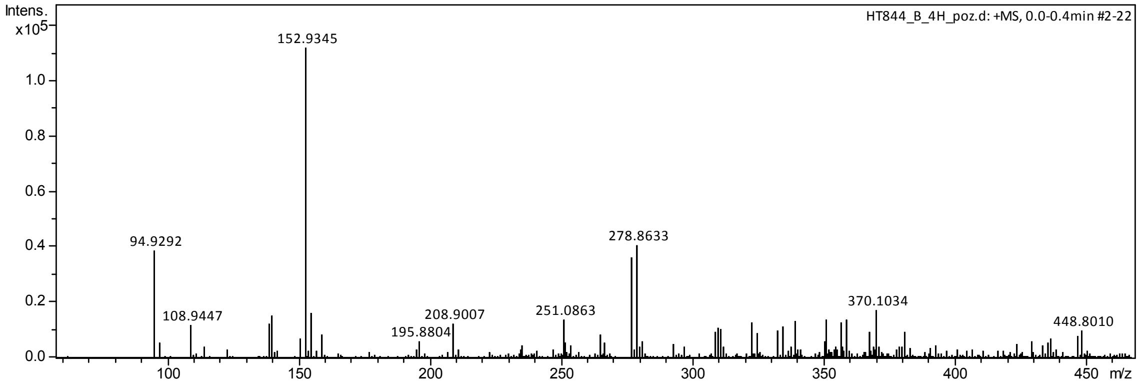
Intens.
x10^5
HT844_B_4H_poz.d: +MS, 0.0-0.4min #2-22
152.9345
94.9292
108.9447
195.8804
208.9007
251.0863
278.8633
370.1034
448.8010
m/z

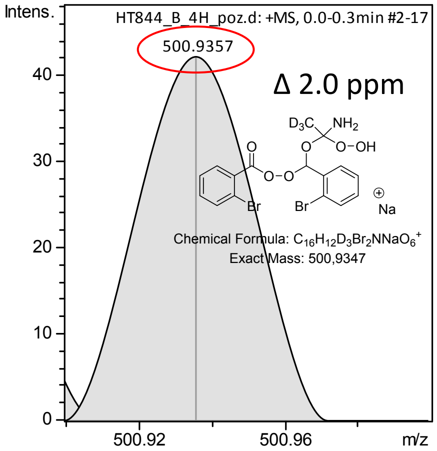
Intens.
HT844_B_4H_poz.d: +MS, 0.0-0.3min #2-17
500.9357
Δ 2.0 ppm
Chemical Formula: C16H12D3Br2NNaO6+
Exact Mass: 500,9347
500.92
500.96
m/z

In order to detect the desired radical intermediates **I** and **II**, proving the radical character of the mechanistic pathway, TEMPO (1 eq.) was added in the reaction mixture. The desired intermediates were detected as TEMPO-trapped molecules.

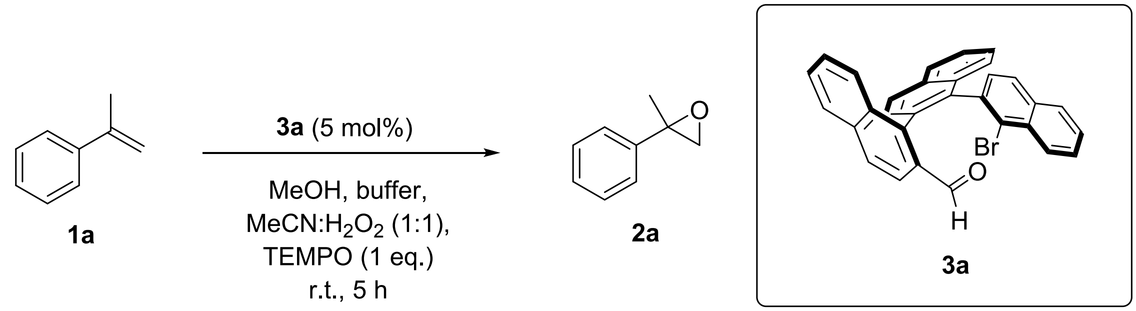

a-Methyl-styrene (24.0 mg, 0.20 mmol) was placed in a round bottom flask followed by catalyst **3a** (4.9 mg, 0.01 mmol). Methanol (0.2 mL), aqueous buffer solution (0.2 mL, 0.6M K_2CO_3 – 4 x 10^{-4} M EDTA disodium salt), acetonitrile (0.02 mL, 0.40 mmol) and 30% aqueous H_2O_2 (0.04 mL, 0.40 mmol) were added consecutively. 2,2,6,6-Tetramethylpiperidinooxy (31.2 mg, 0.20 mmol) was added and the reaction mixture was left stirring at room temperature.

Negative ESI Mode

0 min

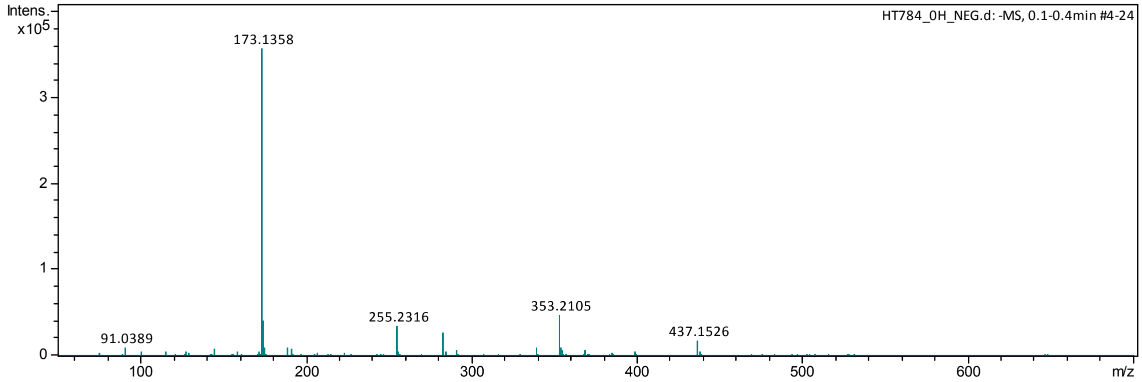

1 h

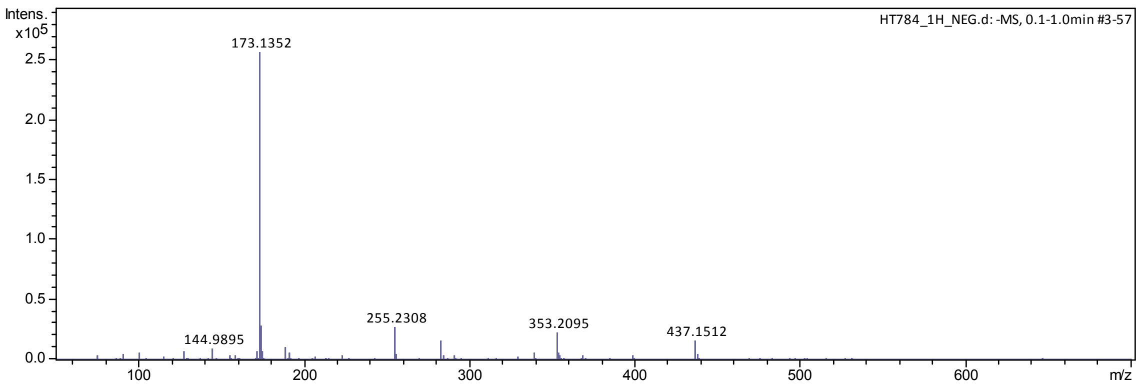
Intens.
x10^5
HT784_1H_NEG.d: -MS, 0.1-1.0min #3-57
173.1352
144.9895
255.2308
353.2095
437.1512
m/z

2 h

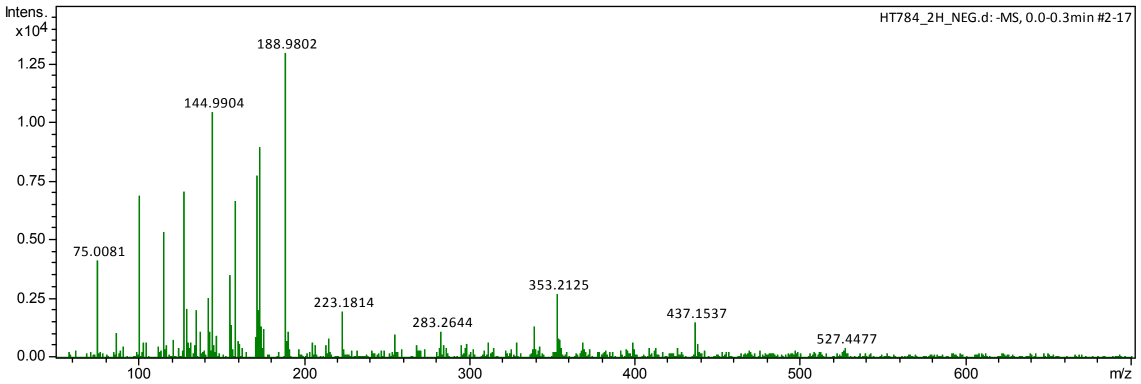
Intens.
x10^4
HT784_2H_NEG.d: -MS, 0.0-0.3min #2-17
188.9802
144.9904
75.0081
223.1814
283.2644
353.2125
437.1537
527.4477
m/z

3 h

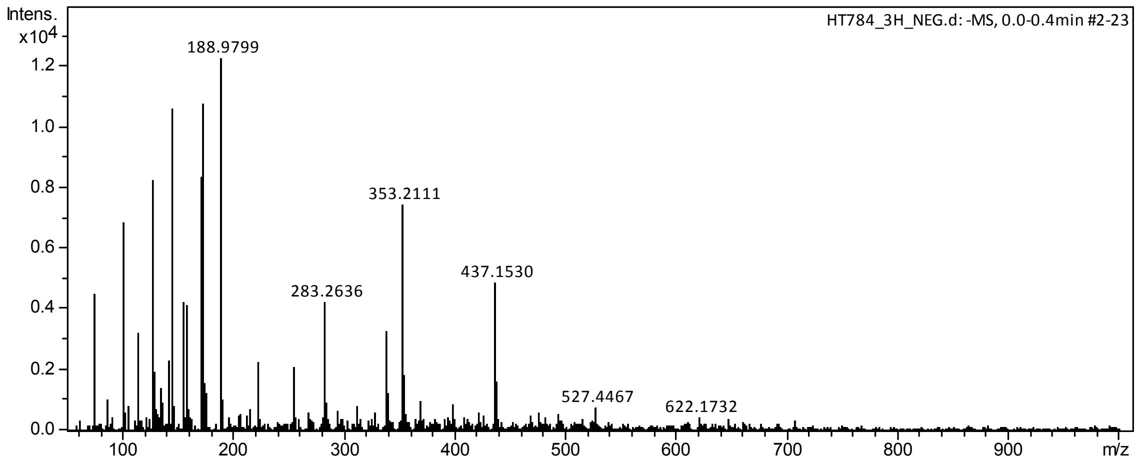
Intens.
x10^4
HT784_3H_NEG.d: -MS, 0.0-0.4min #2-23
188.9799
353.2111
437.1530
283.2636
527.4467
622.1732
m/z

4 h

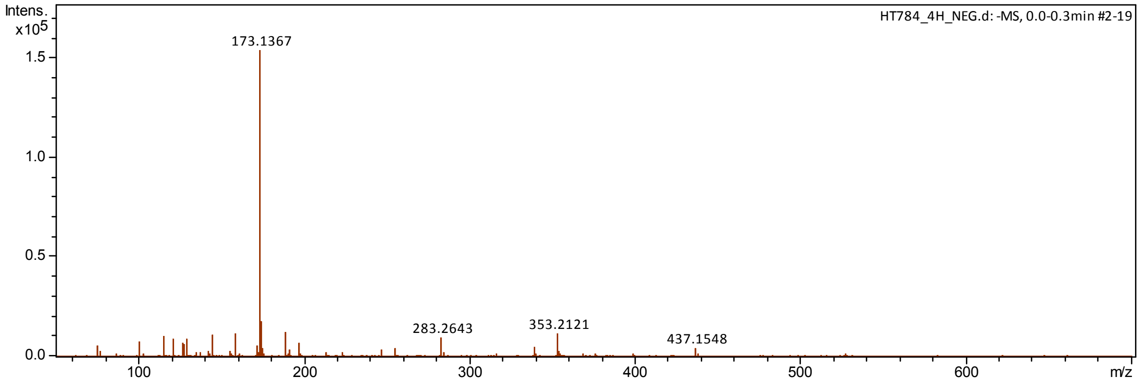
Intens.
x10^5
HT784_4H_NEG.d: -MS, 0.0-0.3min #2-19
173.1367
283.2643
353.2121
437.1548
1.5
1.0
0.5
0.0
100
200
300
400
500
600
m/z

5 h

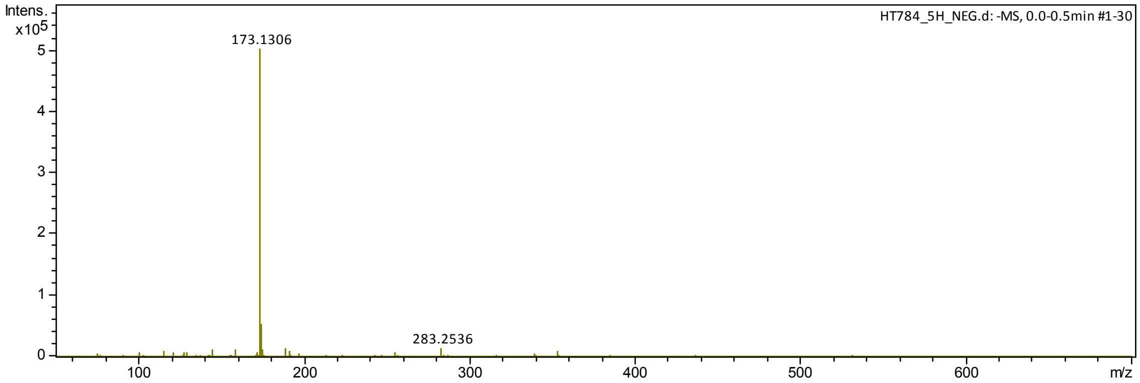
Intens.
x10^5
HT784_5H_NEG.d: -MS, 0.0-0.5min #1-30
173.1306
283.2536
5
4
3
2
1
0
100
200
300
400
500
600
m/z

Positive ESI Mode

0 min

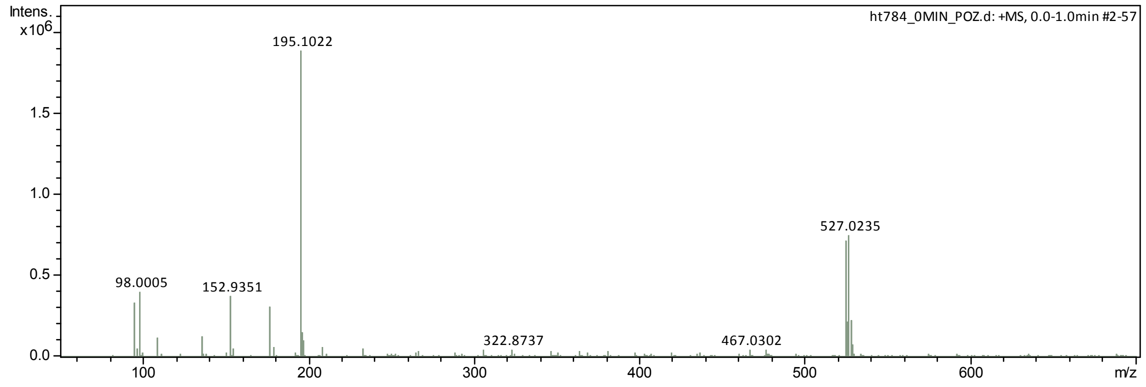

1 h

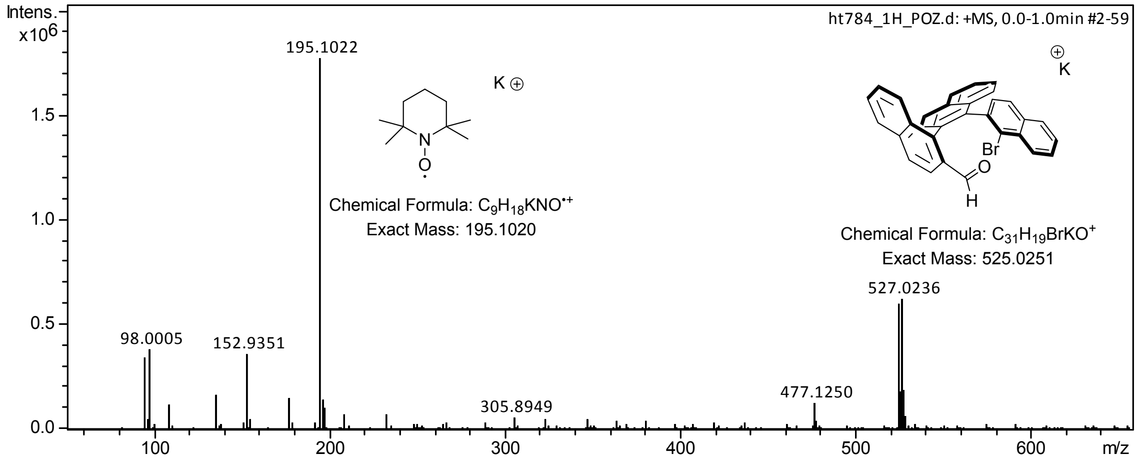

2 h

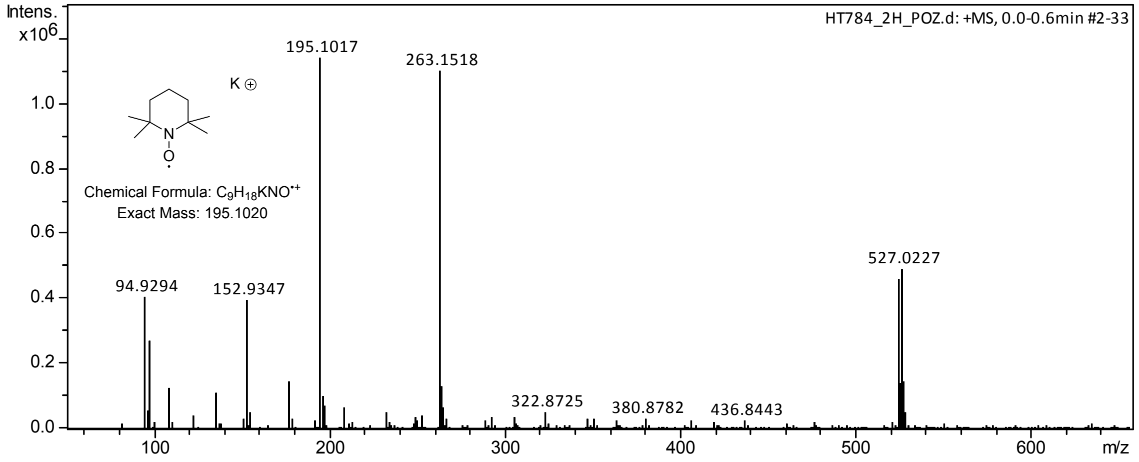

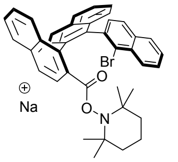

Chemical Formula: $C_{40}H_{36}BrNNaO_2^+$
Exact Mass: 664,1822

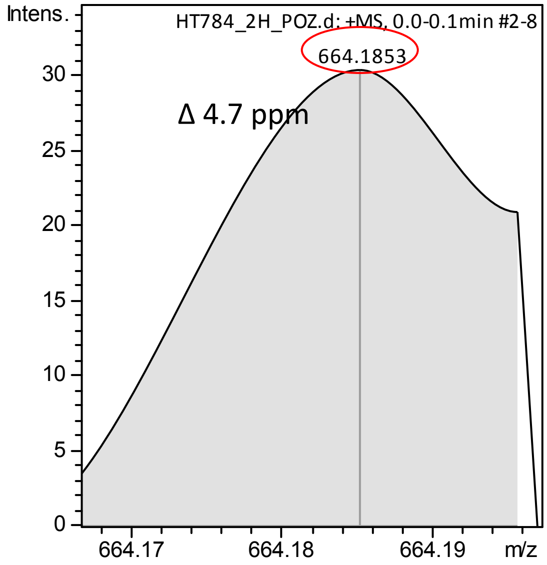

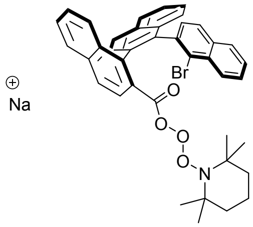

Chemical Formula: $C_{40}H_{36}BrNNaO_4^+$
Exact Mass: 696.1720

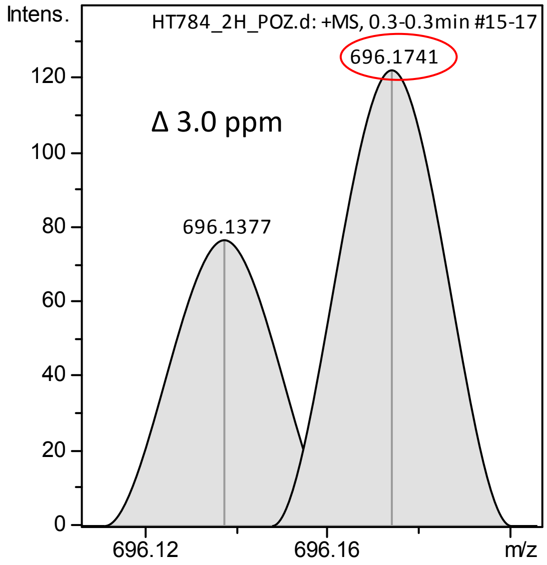

3 h

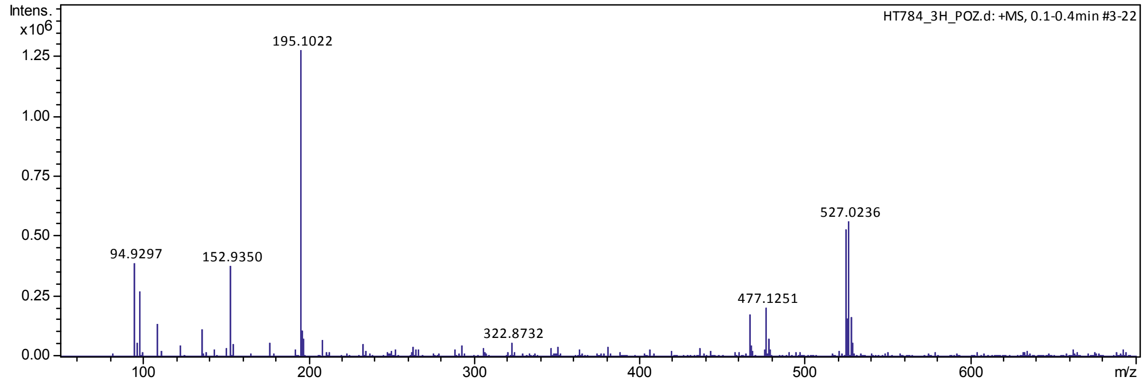
Intens.
x10^6
HT784_3H_POZ.d: +MS, 0.1-0.4min #3-22
195.1022
527.0236
94.9297
152.9350
477.1251
322.8732
m/z

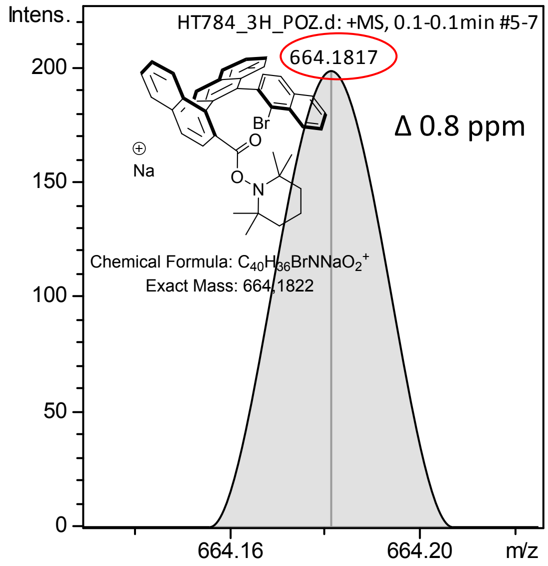
Intens.
HT784_3H_POZ.d: +MS, 0.1-0.1min #5-7
664.1817
Δ 0.8 ppm
Br
O
O
N
Na
Chemical Formula: C40H36BrNNaO2+
Exact Mass: 664.1822
664.16
664.20
m/z

4 h

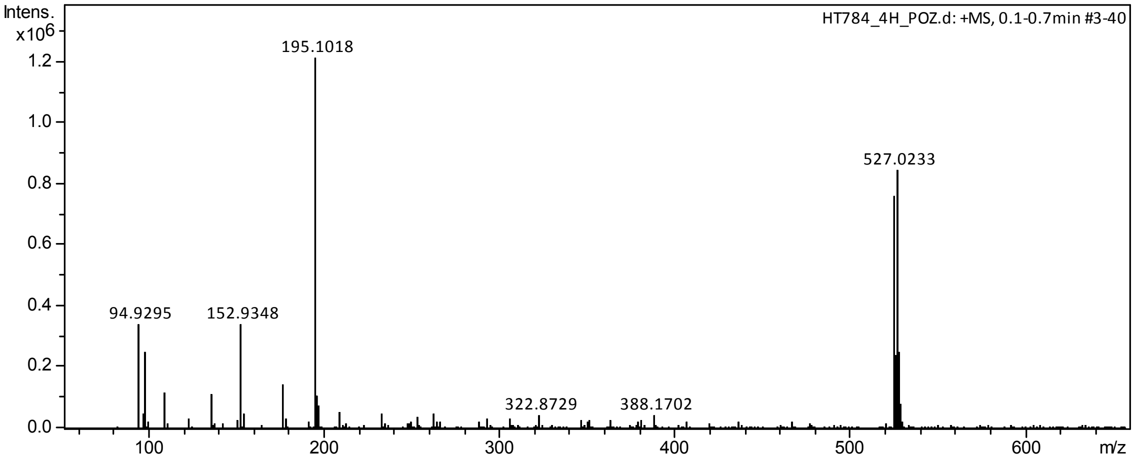
Intens.
x10^6
HT784_4H_POZ.d: +MS, 0.1-0.7min #3-40
195.1018
527.0233
94.9295
152.9348
322.8729
388.1702
m/z

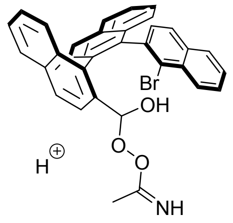

Chemical Formula: $C_{33}H_{25}BrNO_3^+$
Exact Mass: 562.1012

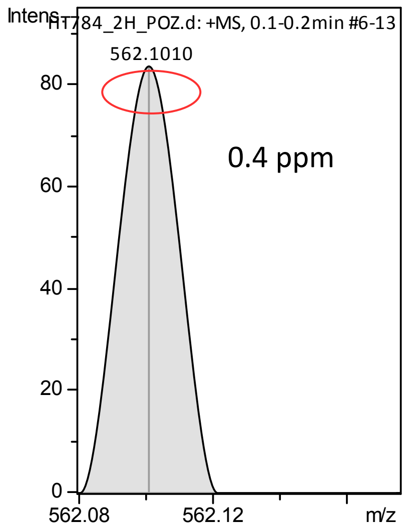

5 h

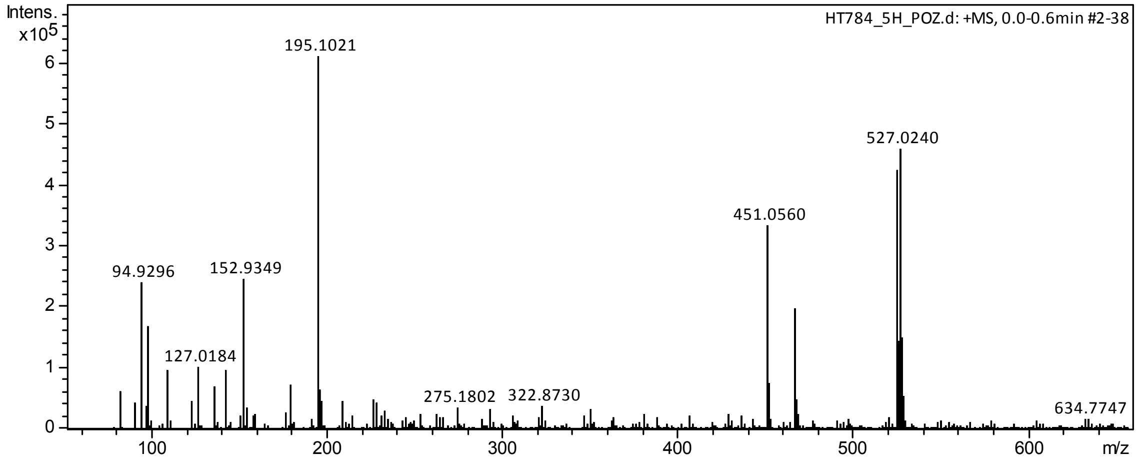

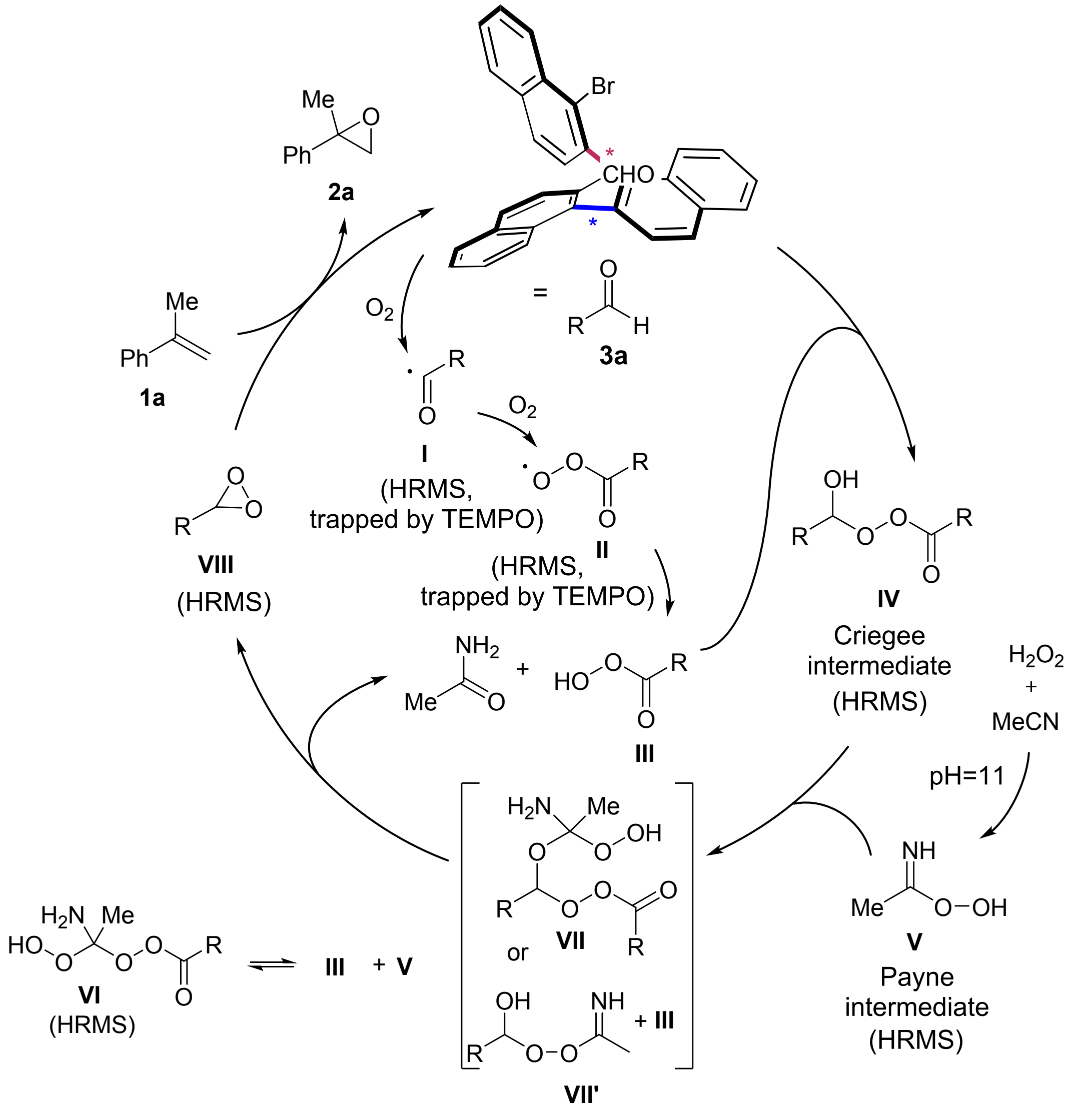

Summarizing the above mechanistic studies, we conclude that the proposed mechanism is presented above. Aerobic autooxidation of the atropoisomeric aldehyde **3a** (detected by HRMS) leads to acyl radical **I** (detected by HRMS, after trapping with TEMPO). Reaction with air (oxygen) leads to peroxy radical intermediate **II** (detected by HRMS, after trapping with TEMPO). Reaction of **II** with another molecule of **3a** leads to Criegee intermediate **IV** (detected by HRMS). Intermediate **IV** reacts with Payne's intermediate **V**, leading to (probably short-lived) postulated species **VII** (detected by HRMS only in the case when 2-bromobenzaldehyde with CD_3CN were employed), which breaks up to form the key dioxirane intermediate **VIII** (detected by HRMS), the acetamide byproduct and regenerates peracid **III**. Another plausible pathway is the transformation of

intermediate **IV** and **V**, to form **III** and **VII'** (detected by HRMS only in the case when **3a** was used as the catalyst in the presence of TEMPO), which can also give rise to dioxirane **VIII** and acetamide. The dioxirane intermediate **VIII** as the active oxidant then reacts with unactivated alkene **1a** to form epoxide **2a**, regenerating aldehyde **3a** to close the catalytic cycle.

References

1. S. K. Kristensen, S. L. R. Laursen, E. Taarning, T. Skrydstrup, *Angew. Chem. Int. Ed.*, **2018**, *57*, 13887-13891.
2. D. Lotter, A. Castrogiovanni, M. Neuburger, C. Sparr, *ACS Cent. Sci.* **2018**, *4*, 656-660.
3. D. Limnios, C. G. Kokotos, *J. Org. Chem.* **2014**, *79*, 10, 4270-4276.
4. Z. Li, M. K. Gupta, T. S. Snowden, *Eur. J. Org. Chem.* **2015**, 7009-7019.
5. K. Yamada, Y. Igarashi, T. Betsuyaku, M. Kitamura, K. Hirata, K. Hioki, M. Kunishima, *Org. Lett.* **2018**, *20*, 2015-2019.
6. L. Bo, Y. Hu, Z. Zhang, M. Balanay, H. Fan, J. Tong, *Polyhedron* **2018**, *154*, 182-188.
7. J. A. Donnelly, J. G. Hoey, *J. Chem. Soc., Perkin Trans. 1*, **1975**, 2364-2368.
